# Behaviour of the XH-*-π and YX-*-π interactions (X, Y = F, Cl, Br and I) in the coronene π-system, as elucidated by QTAIM dual functional analysis with QC calculations†

**DOI:** 10.1039/c8ra01862f

**Published:** 2018-05-03

**Authors:** Satoko Hayashi, Yuji Sugibayashi, Waro Nakanishi

**Affiliations:** Faculty of Systems Engineering, Wakayama University 930 Sakaedani Wakayama 640-8510 Japan hayashi3@sys.wakayama-u.ac.jp nakanisi@sys.wakayama-u.ac.jp +81 73 457 8253 +81 73 457 8252

## Abstract

The dynamic and static nature of XH-*-π and YX-*-π in the coronene π-system (π(C_24_H_12_)) is elucidated by QTAIM dual functional analysis, where * emphasizes the presence of bond critical points (BCPs) in the interactions. The nature of the interactions is elucidated by analysing the plots of the total electron energy densities *H*_b_(*r*_c_) *versus H*_b_(*r*_c_) − *V*_b_(*r*_c_)/2 [=(*ħ*^2^/8*m*)∇^2^*ρ*_b_(*r*_c_)] for the interactions at BCPs, where *V*_b_(*r*_c_) are the potential energy densities at the BCPs. The data for the perturbed structures around the fully optimized structures are employed for the plots in addition to those of the fully optimized structures. The plots are analysed using the polar coordinate of (*R*, *θ*) for the data of the fully optimized structures, while those containing the perturbed structures are analysed using (*θ*_p_, *κ*_p_), where *θ*_p_ corresponds to the tangent line of each plot and *κ*_p_ is the curvature. Whereas (*R*, *θ*) show the static nature, (*θ*_p_, *κ*_p_) represent the dynamic nature of the interactions. All interactions in X–H-*-π(C_24_H_12_) (X = F, Cl, Br and I) and Y–X-*-π(C_24_H_12_) (Y–X = F–F, Cl–Cl, Br–Br, I–I, F–Cl, F–Br and F–I) are classified by pure CS (closed shell) interactions and are characterized as having the vdW nature, except for X–H = F–H and Y–X = F–Cl, F–Br and F–I, which show the typical-HB nature without covalency. The structural features of the complexes are also discussed.

## Abstract 2

The XH-*-π(C_24_H_12_) interactions appear on the outside ring of C_24_H_12_, while YX-*-π(C_24_H_12_) do both on the inside and outside rings.

## Graphical abstract

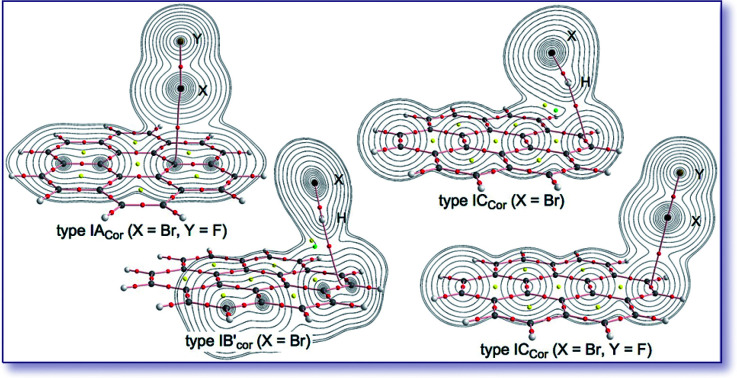

## Introduction

Hydrogen bonds (HBs) and halogen bonds (XBs) are of current and continuous interest. HBs and XBs are fundamentally important for their ability to give rise to molecular association caused by the energy stabilization of the system.^1–11^ The direction-control through the formation of HBs plays a crucial role in all fields of chemical and biological sciences. The opening and closing of the duplex DNA structure in active proliferation at around room temperature is a typical example of the effect of HBs.^12^ HBs also play an important role in the very specific conformation of hormones with the HBs of the dimers controlling the characteristic biological properties.^13^ Conventional HBs of the shared proton interaction type^4^ are formed with atoms of the main group elements, which are usually not very strong in the neutral form (≤ approximately 40 kJ mol^−1^),^1,5^ albeit usually stronger than the van der Waals (vdW) interactions. Contributions from the charge transfer (CT) interaction become more important as the strength of HBs increases in addition to the vdW interactions, where attractive electrostatic interactions and the dispersion force mainly contribute to form the vdW adducts. Conversely, the attractive interactions, between the electrophilic σ*-orbitals of halogen or inter-halogen molecules with the non-bonding orbitals (n-orbitals), must be the driving force for the formation of typical XBs. The nature of XBs has been discussed based on the theoretical background of the molecular orbital description for the bonding and the σ-hole developed on the halogen atoms together with the stability based on the structural aspects.^14^ XBs are applicable to a wide variety of fields in chemical and biological sciences, such as crystal engineering, supramolecular soft matter and nanoparticles.

π-orbitals also give rise to similar HBs and XBs with hydrogen halides and halogen or inter-halogen molecules, respectively. Similar to the case of n-orbitals, π-orbitals act as electron donors to form such adducts. The π-electron systems usually construct planar molecules. Benzene and coronene^15^ are the typical examples of the planar π-systems, together with graphene. Graphene shows unique physical properties. Graphene-based carbon allotropes, such as graphene, graphite, fullerenes^16^ and carbon nanotubes, have attracted considerable attention owing their many potential applications in nanotechnology, including nanoelectronics, energy storage and biosensing.^17–19^ Coronene, a typical planar molecule, is often employed as a model of graphene in the study of adsorption phenomena, even though it is suggested that coronene may, in certain cases, not be a good model of graphene due to the larger HOMO–LUMO gap in coronene.

We recently investigated the dynamic and static behaviour of the XH-*-π and/or YX-*-π interactions (π-HBs and/or π-XBs, respectively) (X, Y = F, Cl, Br and I) in the π-systems of benzene, π(C_6_H_6_),^20,21^ naphthalene, π(C_10_H_8_)^22^ and anthracene, π(C_14_H_10_).^23^ What is the behaviour of the π-HBs and π-XBs interactions in the coronene π-system, π(C_24_H_12_)? What are the differences and similarities in the interactions between π(C_24_H_12_) and π(C_6_H_6_), π(C_10_H_8_) and π(C_14_H_10_)? The nature of the interactions should be elucidated to obtain a better understanding of the chemistry arising from the interactions. The π-HB and π-XB interactions with the planer π(C_24_H_12_) system will supply an important starting point for the interactions with the bent π-systems, such as fullerenes and carbon nanotubes, and the circulene molecules, together with the non-covalent functionalization based on the interactions.^24^

**Scheme 1:**
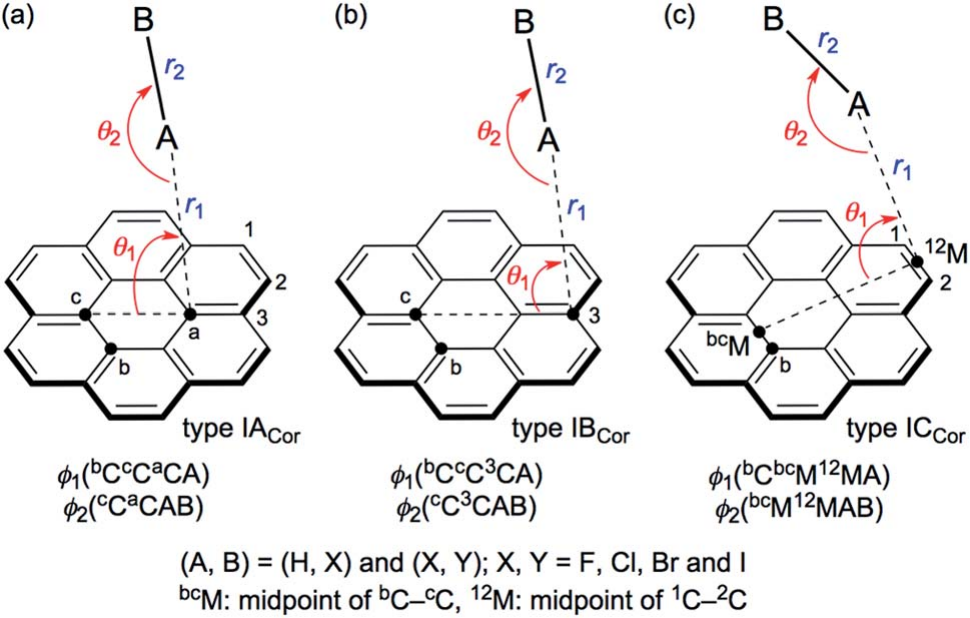
Structures of X–H⋯π(C_24_H_12_) and Y–X⋯π(C_24_H_12_) to be clarified with the definition of structural parameters and types, where A–B = X–H = F–H, Cl–H, Br–H and I–H and A–B = Y–X = F–F, Cl–Cl, Br–Br, I–I, F–Cl, F–Br and F–I.

**Scheme 2:**
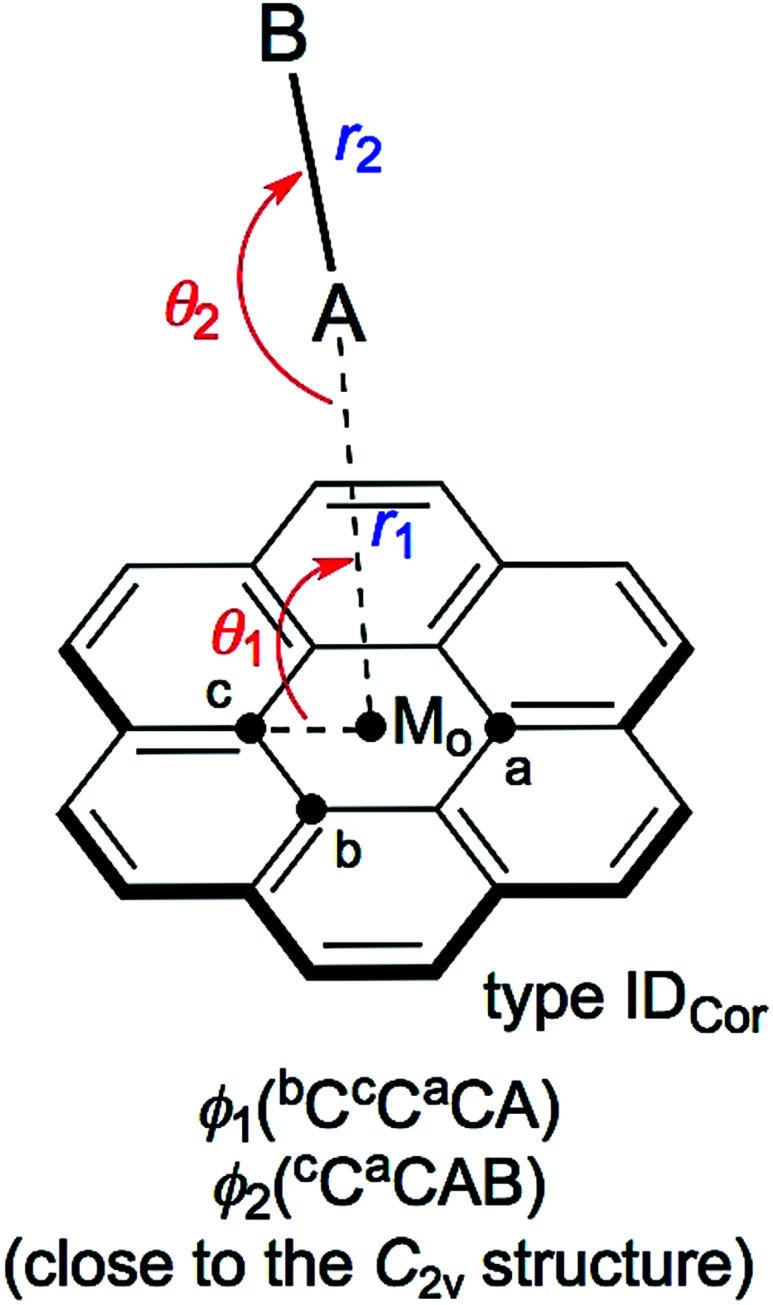
Structures of X–H⋯π(C_24_H_12_) and Y–X⋯π(C_24_H_12_) (A–B = X–H or Y–X: X, Y = F, Cl, Br and I). The structural parameters are defined, together with the types, where M_o_ is the centre point of C_24_H_12_.

Scheme 1 illustrates the structures of X–H⋯π(C_24_H_12_) (X = F, Cl, Br and I) and Y–X⋯π(C_24_H_12_) (Y–X = F–F, Cl–Cl, Br–Br, I–I, F–Cl, F–Br and F–I) to be elucidated in this work.^25^ The scope of the properties in the Y–X⋯π interactions have been demonstrated to be covered by those with Y–X = F–F, F–Cl, F–Br and F–I.^10^ The structural parameters are defined in Scheme 1 together with the types. The structures of the adducts will be called type I_Cor_, if X–H or Y–X appears to interact with the coronene π-system through only a single site of X–H or Y–X. Namely, X–H or Y–X should be placed almost parallel to the normal line of the coronene molecular plane. Type I_Cor_ will be called type IA_Cor_, if X–H or Y–X interacts with a carbon atom in the central ring of coronene. On the other hand, the structure will be type IB_Cor_, when X–H or Y–X is expected to interact with a carbon atom bearing no hydrogen atom in the outside ring of coronene, whereas it will be type IC_Cor_ when X–H or Y–X appears to interact with the midpoint between the adjacent carbon atoms bearing the hydrogen atoms of the outside ring of coronene. Type ID_Cor_ in Scheme 2 is discussed later.

The QTAIM (quantum theory of atoms-in-molecules) approach, introduced by Bader,^26,27^ enables us to analyse the nature of chemical bonds and interactions.^26–30^ Interactions are defined by the corresponding bond paths (BPs), but we must be careful to use the correct terminology with this concept.^31^ The bond critical point (BCP) is an important concept in QTAIM and is a point along the BP at the interatomic surface where the charge density, *ρ*(*r*), reaches a minimum.^32^ This point is denoted by *ρ*_b_(*r*_c_), as are the other QTAIM functions at BCPs, such as the Laplacians of *ρ*_b_(*r*_c_) (∇^2^*ρ*_b_(*r*_c_)), total electron energy densities *H*_b_(*r*_c_), potential energy densities *V*_b_(*r*_c_), kinetic energy densities *G*_b_(*r*_c_) and *k*_b_(*r*_c_) (= *V*_b_(*r*_c_)/*G*_b_(*r*_c_)).^33^

In QTAIM, chemical bonds and interactions are classified by the signs of ∇^2^*ρ*_b_(*r*_c_) and *H*_b_(*r*_c_). Indeed, *H*_b_(*r*_c_) − *V*_b_(*r*_c_)/2 = 0 (∇^2^*ρ*_b_(*r*_c_) = 0) corresponds to the borderline between the classical covalent bonds of shard shell (SS) interactions and the noncovalent closed shell (CS) interactions, but *H*_b_(*r*_c_) = 0 appears to be buried in the noncovalent interactions of CS. (See eqn (S2) of the ESI† for the relation, (*ħ*^2^/8*m*)∇^2^*ρ*_b_(*r*_c_) = *H*_b_(*r*_c_) − *V*_b_(*r*_c_)/2.) Therefore, it seems difficult to characterize the CS interactions, such as van der Waals (vdW) interactions,^34,35^ typical hydrogen bonds (t-HBs),^2,3,36,37^ interactions in molecular complexes formed through charge transfer (CT-MCs),^38^ trihalide ions (X_3_^−^)^38^ and interactions in trigonal bipyramidal adducts formed through CT (CT-TBPs).^38^ Then, we proposed employing the signs of the first derivatives of *H*_b_(*r*_c_) − *V*_b_(*r*_c_)/2 and *H*_b_(*r*_c_) (d(*H*_b_(*r*_c_) − *V*_b_(*r*_c_)/2)/d*r* and d*H*_b_(*r*_c_)/d*r*, respectively) to characterize these interactions. The borderline between CT-MC and CT-TBP (containing X_3_^−^) is defined by d(*H*_b_(*r*_c_) − *V*_b_(*r*_c_)/2)/d*r* = 0, while that between vdW and t-HB is by d*H*_b_(*r*_c_)/d*r* = 0, as shown by the experimental results, with the presumption that the CS interactions are reasonably characterized as expected. The proposed definitions for the classification of interactions are summarized in Table S1 of the ESI,† together with those tentatively proposed,^39^ for convenience of discussion.

Recently, we proposed QTAIM dual functional analysis (QTAIM-DFA),^40–43^ according to QTAIM.^26–29,44,45^ QTAIM-DFA provides an excellent approach for evaluating, classifying and understanding weak to strong interactions in a unified form.^40–43^ In QTAIM-DFA, *H*_b_(*r*_c_) are plotted *versus H*_b_(*r*_c_) − *V*_b_(*r*_c_)/2 [= (*ħ*^2^/8*m*) ∇^2^*ρ*_b_(*r*_c_)]. In our treatment, data for perturbed structures around fully optimized structures are employed for the plots, in addition to those from the fully optimized structures.^40–43^ QTAIM-DFA can incorporate the classification of interactions by the signs of ∇^2^*ρ*_b_(*r*_c_), *H*_b_(*r*_c_), d(*H*_b_(*r*_c_) − *V*_b_(*r*_c_)/2)/d*r* and d*H*_b_(*r*_c_)/d*r* with the definitions, tentatively proposed.^46^ We have proposed the concept of “the dynamic nature of interactions” which originates from the data containing the perturbed structures.^40*a*,41–43^ Data from the fully optimized structures correspond to the static nature of interactions. QTAIM-DFA is applied to typical chemical bonds and interactions and rough criteria are established. The rough criteria can distinguish the chemical bonds and interactions in question from other types of interactions. QTAIM-DFA and these criteria are explained in the ESI using Schemes S1 and S2, Fig. S1 and eqn (S1)–(S6).† The basic concept of the QTAIM approach is also surveyed.

We consider QTAIM-DFA to be well-suited to elucidate the dynamic and static nature of the π-HBs and π-XBs interactions in π(C_24_H_12_), even though static behaviour of π-HBs in π(C_24_H_12_) has been discussed.^47,48^ In this study, we present the results of the investigations on the nature of the interactions. The interactions are classified and characterized based on the above criteria.

## Methodological details in calculations

The structures were optimized using the Gaussian 09 programme package.^49^ The basis set system (BSS) from the Sapporo Basis Set Factory^50^ (BSS-S) was employed for the calculations. In the calculations with BSS-SA, the (7433211/743111/7411/2 + 1s1p) type was employed for I, the (743211/74111/721/2 + 1s1p) type for Br, the (63211/6111/31/2 + 1s1p) type for Cl and the (6211/311/21/2 + 1s1p) type for F with the (6211/311/21/2 + 1s1p) type for C and the (411/21/2 + 1s1p) type for H. BSS-SA was applied for the calculations at the M06-2X (M06-2X/BSS-SA) level of density functional theory (DFT). Optimized structures were confirmed by the frequency analysis. QTAIM functions were similarly calculated using the Gaussian 09 programme package^49^ with the same method of the optimizations and the data were analysed with the AIM2000 ^51^ and AIMAll^52^ programmes. The results obtained at the M06-2X/BSS-SA level of theory will be mainly discussed in the text.

For BSS-SB, the (743321/74321/742 + 1s1p) type was employed for I, the (74321/7421/72 + 1s1p) type for Br, the (6321/621/3 + 1s1p) type for Cl and the (621/31/2 + 1s1p) type for F with the (621/31/2 + 1s1p) type for C and the (31/3 + 1s1p) type for H. The calculations were also performed at the M06-2X/BSS-SB level of theory to search for the potential energy surface minima as the pre-optimizations, when necessary. M06-2X/BSS-SB is also employed to confirm the minima and BPs with BCPs around the interactions in question, if they are not obtained satisfactorily with M06-2X/BSS-SA.

The results obtained using M06-2X/BSS-SB are discussed in Tables S1 and S2 of the ESI† and/or the text, if necessary. M06-2X/BSS-SA was also applied to the benzene π-system for convenience of comparison. The calculations were similarly performed using MP2/6-311G(d,p)^53,54^ for the convenience of comparison. The results are collected in the ESI.†

Normal coordinates of internal vibrations (NIV) obtained by the frequency analysis were employed to generate the perturbed structures.^41,42^ This method is explained by eqn (1). A *k*-th perturbed structure (**S**_*kw*_) was generated by the addition of the normal coordinates of the *k*-th internal vibration (**N**_*k*_) to the standard orientation of the fully optimized structure (**S**_o_) in the matrix representation.^55^ The coefficient *f*_*kw*_ in eqn (1) controls the difference in the structures between **S**_*kw*_ and **S**_o_: *f*_*kw*_ are determined to satisfy eqn (1) for the interaction in question, where *r* and *r*_o_ show the distances under investigation in the perturbed and fully optimized structures, respectively, and *a*_o_ is the Bohr radius (0.52918 Å).^56^ Namely, the perturbed structures with NIV correspond to those with *r* being elongated or shortened by 0.05*a*_o_ or 0.1*a*_o_, relative to *r*_o_. **N**_*k*_ of five digits are used to predict **S**_*kw*_. We refer to this method to generate the perturbed structures as NIV.

1 **S**_*kw*_ = **S**_o_ + *f*_*kw*_ × **N**_*k*_

2 *r* = *r*_o_*+ wa*_o_ (*w* = (0), ±0.05 and ±0.1; *a*_o_ = 0.52918 Å)

3 *y* = *a*_o_ + *a*_1_*x* + *a*_2_*x*^2^ + *a*_3_*x*^3^

In the QTAIM-DFA treatment, *H*_b_(*r*_c_) are plotted *versus H*_b_(*r*_c_) − *V*_b_(*r*_c_)/2 for five data points of *w* = 0, ±0.05 and ±0.1 in eqn (2). Each plot is analysed using a regression curve of the cubic function as shown in eqn (3), where (*x*, *y*) are (*H*_b_(*r*_c_) − *V*_b_(*r*_c_)/2, *H*_b_(*r*_c_)) (*R*_*c*_^2^ (square of correlation coefficient) > 0.99999 in usual).^43^

## Results and discussion

### Optimizations of X–H⋯π(C_24_H_12_) and Y–X⋯π(C_24_H_12_)

The structures were optimized for X–H⋯π(C_24_H_12_) and Y–X⋯π(C_24_H_12_). The optimizations were initially performed with M06-2X/BSS-SB, assuming the *C*_1_ symmetry. The X–H and Y–X components were placed in close proximity to types IA_Cor_, IB_Cor_ and IC_Cor_ together with type ID_Cor_ (see Schemes 1 and 2) in the optimization processes, but the systematic search was not performed. Each adduct finally converged to a structure with the *C*_1_ symmetry. The structures were optimized again with M06-2X/BSS-SA. The optimized structures are confirmed by all positive frequencies after the frequency analysis. Then, the *C*_1_ structures with all positive frequencies were further optimized, assuming the *C*_s_ symmetry in the cases where the *C*_1_ structures appeared to be very close to the *C*_s_ symmetry. The frequency analysis was also performed on the *C*_s_ structures. The IB_Cor_ and IC_Cor_ types were predicted for X–H⋯π(C_24_H_12_), while the IA_Cor_ and IC_Cor_ types were used for Y–X⋯π(C_24_H_12_), when optimized with M06-2X/BSS-SA.

**Fig. 1:**
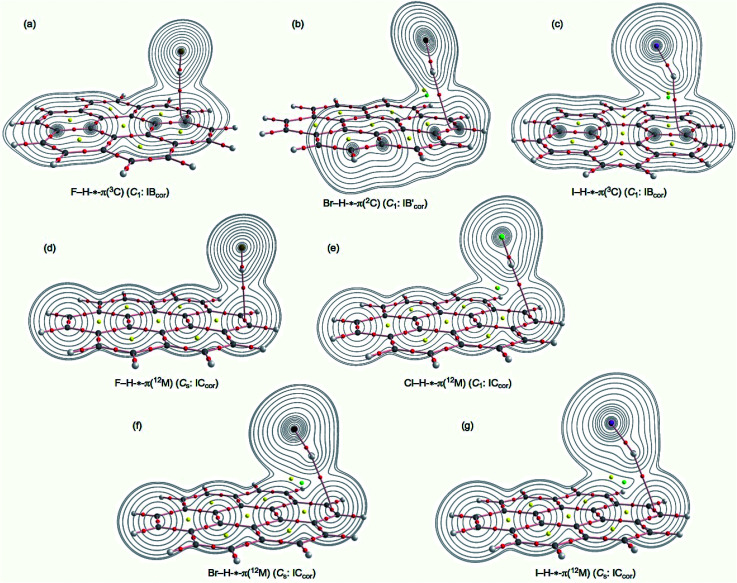
Molecular graphs for F–H-*-π(C_24_H_12_) (*C*_1_: IB_Cor_) (a), Br–H-*-π(C_24_H_12_) (*C*_1_: 
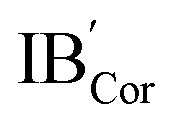
) (b), I–H-*-π(C_24_H_12_) (*C*_1_: IB_Cor_) (c), F–H-*-π(C_24_H_12_) (*C*_s_: IC_Cor_) (d), Cl–H-*-π(C_24_H_12_) (*C*_1_: IC_Cor_) (e), Br–H-*-π(C_24_H_12_) (*C*_s_: IC_Cor_) (f) and I–H-*-π(C_24_H_12_) (*C*_s_: IC_Cor_) (g), calculated with M06-2X/BSS-SA. BCPs are denoted by red dots, RCPs by yellow dots, CCPs by green dots and BPs by pink lines. Carbon atoms are in black and hydrogen atoms are in grey, with fluorine, chlorine, bromine and iodine atoms in dark yellow, green, dark purple and purple, respectively. The contour plot of *ρ*(*r*) is also drawn for each on the plane containing the H-*-^3^C(C_24_H_12_) moiety for type IB_Cor_ with the H-*-^2^C(C_24_H_12_) moiety for type 
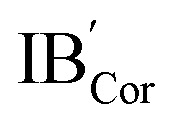
 or on the plane of the H-*-^12^M(C_24_H_12_) moiety for type IC_Cor_, where the contour plot is drawn on each plane.

**Fig. 2:**
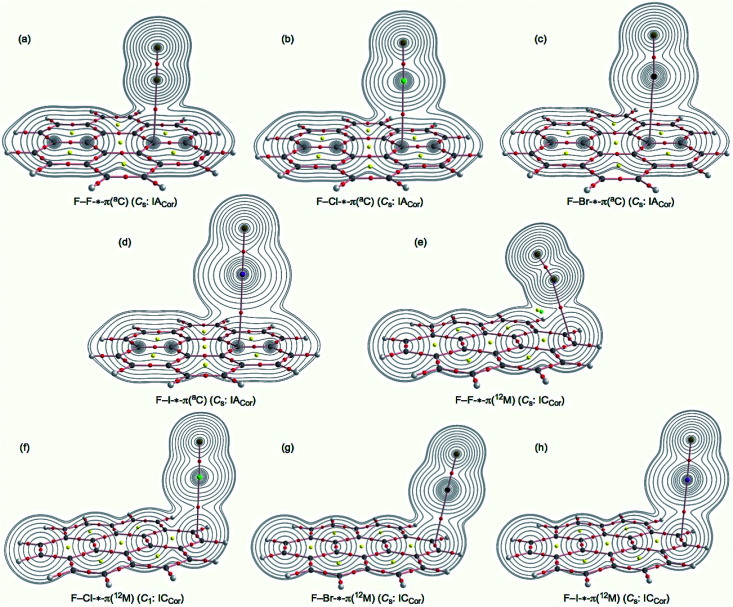
Molecular graphs for F–F-*-π(C_24_H_12_) (*C*_s_: IA_Cor_) (a), F–Cl-*-π(C_24_H_12_) (*C*_s_: IA_Cor_) (b), F–Br-*-π(C_24_H_12_) (*C*_s_: IA_Cor_) (c), F–I-*-π(C_24_H_12_) (*C*_s_: IA_Cor_) (d), F–F-*-π(C_24_H_12_) (*C*_s_: IC_Cor_) (e), F–Cl-*-π(C_24_H_12_) (*C*_1_: IC_Cor_) (f), F–Br-*-π(C_24_H_12_) (*C*_s_: IC_Cor_) (g) and F–I-*-π(C_24_H_12_) (*C*_s_: IC_Cor_) (h), calculated with M06-2X/BSS-SA. BCPs are denoted by red dots, RCPs by yellow dots and BPs by pink lines. Carbon atoms are in black and hydrogen atoms are in grey, with fluorine, chlorine, bromine and iodine atoms in dark yellow, green, dark purple and purple, respectively. The contour plot of *ρ*(*r*) is also drawn for each on the plane containing the X-*-^3^C(C_24_H_12_) moiety for type IB_Cor_ or on the plane of X-*-^12^M(C_24_H_12_) moiety for type IC_Cor_, where the contour plot is drawn on each plane.

All positive frequencies were confirmed for all adducts, except for F–H⋯π(^3^C) (*C*_1_: IB_Cor_), Cl–H⋯π(^12^M) (*C*_1_: IC_Cor_), Cl–Cl⋯π(^*a*^C) (*C*_1_: IA_Cor_), Cl–Cl⋯π(^12^M) (*C*_1_: IC_Cor_) and F–Cl⋯π(^12^M) (*C*_1_: IC_Cor_). The motion of each imaginary frequency mainly corresponds to the angular displacements between π(C_24_H_12_) and X–H or Y–X. In the case of Cl–H⋯π(^2^C) (*C*_1_: IB_Cor_), the calculation converged to Cl–H⋯π(^12^M) (*C*_1_: IC_Cor_), which did not give positive frequencies only after the frequency analysis. Table 1 summarizes the structural parameters (*r*_1_, *r*_2_, *θ*_1_, *θ*_2_, *ϕ*_1_ and *ϕ*_2_) of X–H⋯π(C_24_H_12_) and Y–X⋯π(C_24_H_12_), defined in Scheme 1. The optimized structures are not shown in figures, but a number of them can be observed in Fig. 1 and 2. The magnitudes of the *θ*_1_, *θ*_2_, *ϕ*_1_ and *ϕ*_2_ values are close to 90°, 180°, 90° and 180° (or 0°), respectively, for the most cases.

**Table 1:** Structural parameters for X–H⋯π(C_24_H_12_) and Y–X⋯π(C_24_H_12_), optimized with M06-2X/BSS-SA^a^^,^^b^

Y–X-*-π(C_24_H_12_), (symmetry: type)	*r*_1_, (Å)	*r*_2_, (Å)	*θ*_1_, (°)	*θ*_2_, (°)	*ϕ*_1_, (°)	*ϕ*_2_, (°)	Δ*E*_ES_^c^^,^^d^, (kJ mol^−1^)	Δ*E*_Ent_^c^^,^^e^, (kJ mol^−1^)
F–H⋯π(^3^C) (*C*_1_: IB_Cor_)^f^	2.2609	0.9243	80.09	169.69	−89.91	180.00	−17.5	−15.5
Br–H⋯π(^2^C) (*C*_1_: 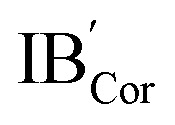 )^g^	2.6197	1.4243	78.28	166.98	−114.35	52.29	−16.8	−10.0
I–H⋯π(^3^C) (*C*_1_: IB_Cor_)	2.6427	1.6219	77.03	162.89	−108.52	−12.06	−16.9	−11.2
F–H⋯π(^12^M) (*C*_s_: IC_Cor_)	2.1815	0.9261	83.01	177.80	−90.00	180.00	−19.4	−15.1
Cl–H⋯π(^12^M) (*C*_1_: IC_Cor_)^f^	2.4502	1.2840	68.93	179.73	−90.00	179.97	−16.1	−14.9
Br–H⋯π(^12^M) (*C*_s_: IC_Cor_)	2.5236	1.4244	68.68	167.96	−90.00	0.00	−17.2	−10.3
I–H⋯π(^12^M) (*C*_s_: IC_Cor_)	2.5847	1.6222	68.98	164.38	−90.00	0.00	−17.3	−12.2
F–F⋯π(^*a*^C) (*C*_s_: IA_Cor_)	2.7873	1.3685	90.62	177.95	−89.96	0.00	−7.5	−3.1
Cl–Cl⋯π(^*a*^C) (*C*_1_: IA_Cor_)^f^	3.0381	1.9950	90.95	178.16	−89.95	0.03	−16.0	−13.5
Br–Br⋯π(^*a*^C) (*C*_s_: IA_Cor_)	3.1293	2.2912	90.95	177.03	−89.95	0.00	−20.1	−14.1
I–I⋯π(^*a*^C) (*C*_s_: IA_Cor_)	3.3116	2.6768	89.61	178.88	−89.92	180.00	−23.5	−20.2
F–Cl⋯π(^*a*^C) (*C*_s_: IA_Cor_)	2.9409	1.6257	90.82	177.56	−90.07	0.00	−20.4	−16.5
F–Br⋯π(^*a*^C) (*C*_s_: IA_Cor_)	3.0096	1.7632	92.21	174.94	−90.11	0.00	−27.4	−22.8
F–I⋯π(^*a*^C) (*C*_s_: IA_Cor_)	3.1554	1.9216	93.14	178.55	−90.00	0.00	−35.6	−32.0
F–F⋯π(^12^M) (*C*_s_: IC_Cor_)	2.9010	1.3677	72.64	162.12	−90.00	0.00	−7.0	−3.5
Cl–Cl⋯π(^12^M) (*C*_1_: IC_Cor_)^f^	3.0107	1.9990	87.28	178.12	−90.00	0.00	−14.3	−13.2
Br–Br⋯π(^12^M) (*C*_1_: IC_Cor_)	3.0801	2.2975	90.46	176.98	−88.52	65.27	−17.8	−13.3
I–I⋯π(^12^M) (*C*_s_: IC_Cor_)	3.3370	2.6788	86.08	179.26	−90.00	180.00	−20.6	−16.5
F–Cl⋯π(^12^M) (*C*_1_: IC_Cor_)^f^^,^^h^	2.8523	1.6323	91.82	178.00	−90.02	−0.09	−21.7	−19.3
F–Br⋯π(^12^M) (*C*_s_: IC_Cor_)	2.8616	1.7699	98.68	178.24	−90.00	180.00	−30.0	−25.3
F–I⋯π(^12^M) (*C*_s_: IC_Cor_)	3.0277	1.9269	94.21	179.61	−90.00	0.00	−36.5	−32.8

a See text for BSS-SA.

b See Scheme 1 for the definition of the structural parameters.

c Δ*E* = *E*(X–H⋯π(C_24_H_12_)/Y–X⋯π(C_24_H_12_)) − (*E*(X–H/Y–X) + *E*(C_24_H_12_)).

d Δ*E*_ES_ represents Δ*E* on the energy surface.

e Δ*E*_Ent_ represents Δ*E* with the correction of the heat of enthalpy.

f One imaginary frequency being predicted for each, of which motion mainly corresponds to the angular displacements between π(C_24_H_12_) and X–H or Y–X.

g Br–H being placed above the midpoint between ^2^C and ^3^C, which is defined by type 
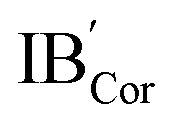
. In this case, the *r*_1_ value is measured from ^2^C.

h Close to the *C*_s_ symmetry, where Cl in F–Cl pointing to ^12^M, the midpoint between ^1^C and ^2^C.

However, significant deviations are observed in some cases. The (*ϕ*_1_, *ϕ*_2_) values of (−114.4°, 52.3°) for Br–H⋯π(^2^C) (*C*_1_: 
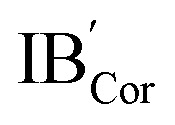
) are the typical example, taken from the intermediate structure between Br–H⋯π(^2^C) (*C*_1_: IB_Cor_) and typical Br–H⋯π(^12^M) (*C*_s_: IC_Cor_). The lack of convergence of Cl–H⋯π(C_24_H_12_) to the IB_Cor_ type with all positive frequencies is related to the formation of Br–H⋯π(^2^C) (*C*_1_: 
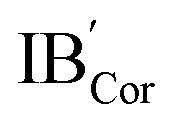
).

This is a very gentle potential energy surface around the inter-conversion between Cl–H⋯π(C_24_H_12_) (IC_Cor_) and the related structure. Similarly, for the cases discussed above, all positive frequencies only were not predicted for F–H⋯π(^3^C) (*C*_1_: IB_Cor_) and Cl–H⋯π(^12^M) (*C*_1_: IC_Cor_) in X–H⋯π(C_24_H_12_). This is also owing to the very gentle potential energy surface around the motions of the imaginary frequencies for Cl–Cl⋯π(^*a*^C) (*C*_1_: IA_Cor_), Cl–Cl⋯π(^12^M) (*C*_1_: IC_Cor_) and F–Cl⋯π(^12^M) (*C*_1_: IC_Cor_) in Y–X⋯π(C_24_H_12_). Nevertheless, with the exception of Cl–H⋯π(^12^M) (*C*_1_: IC_Cor_), positive frequencies only are predicted for these cases when the calculations are performed with M06-2X/BSS-SB. The results are collected in Table S1 of the ESI.†

The energy differences between X–H⋯π(C_24_H_12_) and Y–X⋯π(C_24_H_12_) and the components, Δ*E* (= *E*(X–H⋯π(C_24_H_12_)/Y–X⋯π(C_24_H_12_)) − (*E*(X–H/Y–X) + *E*(C_24_H_12_))) (Δ*E*_ES_ and Δ*E*_Ent_), are also given in Table 1. Δ*E*_ES_ and Δ*E*_Ent_ represent Δ*E* on the energy surface and Δ*E* with the collections by the enthalpy for the formation of the adducts at 25 °C, respectively. The plot of Δ*E*_Ent_*versus* Δ*E*_ES_ gave a (very) good correlation (*y* = 0.992*x* + 3.85: *R*_c_^2^ = 0.955 (*n* (number of data points) = 21)) even though the data for Cl–H⋯π(^12^M) (*C*_1_: IC_Cor_), Br–H⋯π(^12^M) (*C*_s_: IC_Cor_), Br–H⋯π(^2^C) (*C*_1_: 
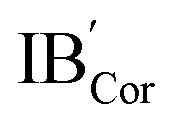
), Br–Br⋯π(^*a*^C) (*C*_s_: IA_Cor_) and Cl–Cl⋯π(^12^M) (*C*_1_: IC_Cor_) appear to deviate somewhat from the correlation (Fig. S2 of the ESI†). A much better correlation was obtained if the data for the five species are omitted from the correlation (*y* = 1.000*x* + 3.90: *R*_c_^2^ = 0.986 (*n* = 16)). Therefore, Δ*E*_ES_ can be used for the discussion of Δ*E*.

After the elucidation of the structural feature of X–H⋯π(C_24_H_12_) and Y–X⋯π(C_24_H_12_), molecular graphs, contour plots, negative Laplacians and trajectory plots are examined next.

### Molecular graphs, contour plots, negative Laplacians and trajectory plots for X–H-*-π(C_24_H_12_) and Y–X-*-π(C_24_H_12_)

Fig. 1 illustrates the molecular graphs for F–H-*-π(C_24_H_12_) (*C*_1_: IB_Cor_), Br–H-*-π(C_24_H_12_) (*C*_1_: 
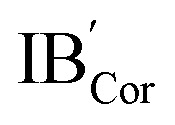
), I–H-*-π(C_24_H_12_) (*C*_1_: IB_Cor_), F–H-*-π(C_24_H_12_) (*C*_s_: IC_Cor_), Cl–H-*-π(C_24_H_12_) (*C*_1_: IC_Cor_), Br–H-*-π(C_24_H_12_) (*C*_s_: IC_Cor_) and I–H-*-π(C_24_H_12_) (*C*_s_: IC_Cor_), calculated with M06-2X/BSS-SA. Each molecular graph contains the contour plot of *ρ*(*r*) drawn on the plane containing the H-*-^3^C moiety for F–H-*-π(C_24_H_12_) (IB_Cor_) and I–H-*-π(C_24_H_12_) (IB_Cor_) with the H-*-^2^C moiety for Br–H-*-π(C_24_H_12_) (
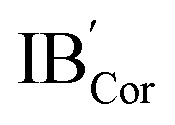
) or on the plane of H-*-^12^M moiety for X–H-*-π(C_24_H_12_) (IC_Cor_), albeit partially. Fig. 2 shows the molecular graphs for the IA_Cor_ and IC_Cor_ types of F–X-*-π(C_24_H_12_) (X = F, Cl, Br and I), calculated with M06-2X/BSS-SA. The contour plot of *ρ*(*r*) is drawn for each adduct partially, similar to Fig. 1.

In Fig. 1, all expected BCPs are clearly observed, including those for the XH-*-π and YX-*-π interactions in question, together with ring critical points (RCPs) and cage critical points (CCPs), if such exist. The structural feature is visualized well by the molecular graphs. The BPs for H-*-π and X-*-π in question seem linear for most of X–H-*-π(C_24_H_12_) and Y–X-*-π(C_24_H_12_), although some seem somewhat bending. BCPs are well located at the (three-dimensional) saddle points of *ρ*(*r*). Negative Laplacians and trajectory plots are drawn for X–H-*-π(C_24_H_12_), similar to Fig. 1 and are shown in Fig. S3 and S4 of the ESI,† respectively. Negative Laplacians and trajectory plots are also drawn for Y–X-*-π(C_24_H_12_), similar to Fig. 2, and are shown in Fig. S5 and S6 of the ESI,† respectively. The behaviour of the BCPs is well-visualized through ∇^2^*ρ*(*r*) as shown in Fig. S3 and S5 of the ESI.† All BCPs in X–H-*-π(C_24_H_12_) and Y–X-*-π(C_24_H_12_) are placed in the blue areas of the negative Laplacians; therefore, the interactions corresponding to the BCPs should be classified by the CS interactions. The space around the species around the interactions in question is well divided into atoms, as demonstrated in Fig. S4 and S6 of the ESI.†

### Survey of X–H-*-π(C_24_H_12_) and Y–X-*-π(C_24_H_12_) interactions, evaluated with M06-2X/BSS-SA

How can the X–H-*-π(C_24_H_12_) and Y–X-*-π(C_24_H_12_) interactions be described? The interactions can be defined by the corresponding BPs, although we must be careful to use the correct terminology with this concept.^31^ As shown in Fig. 1 and 2, BPs for the adducts appear to be straight, with the exception of X–H-*-π(C_24_H_12_) (*C*_1_: IB_Cor_) (X = Br and I) and Y–X-*-π(^12^M) (*C*_1_: IC_Cor_) (Y–X = F–Cl and Br–Br). The lengths of BPs (*r*_BP_) and the straight-line distances (*R*_SL_) evaluated with M06-2X/BSS-SA, are collected in Table S3 of the ESI† together with the Δ*r*_BP_ (= *r*_BP_ − *R*_SL_) values. The Δ*r*_BP_ value are 0.68 Å for F–Cl-*-π(^12^M) (*C*_1_: IC_Cor_), 0.41 Å for Br–Br-*-π(^12^M) (*C*_1_: IC_Cor_), 0.35 Å for Br–H-*-π(^2^C) (*C*_1_: IB_Cor_) and 0.17 Å for I–H-*-π(^3^C) (*C*_1_: IB_Cor_). However, the Δ*r*_BP_ values are smaller than 0.064 Å for X–H-*-π(C_24_H_12_) and smaller than 0.015 Å for Y–X-*-π(C_24_H_12_) (*C*_s_: IA_Cor_) (X, Y = F, Cl, Br and I), as shown in Table S3.† Therefore, the H-*-π and X-*-π interactions in the coronene π-system can be approximated as straight lines, except for the four species, although Δ*r*_BP_ = 0.064 Å for F–H-*-π(^3^C) (*C*_1_: IB_Cor_). The plot of *r*_BP_*versus R*_SL_ for the adducts gave an excellent correlation (*y* = 0.966*x* + 0.1079; *R*_c_^2^ = 0.999 (*n* = 16)), if the data of the four species are neglected from the correlation (not shown in the figure).

**Fig. 3:**
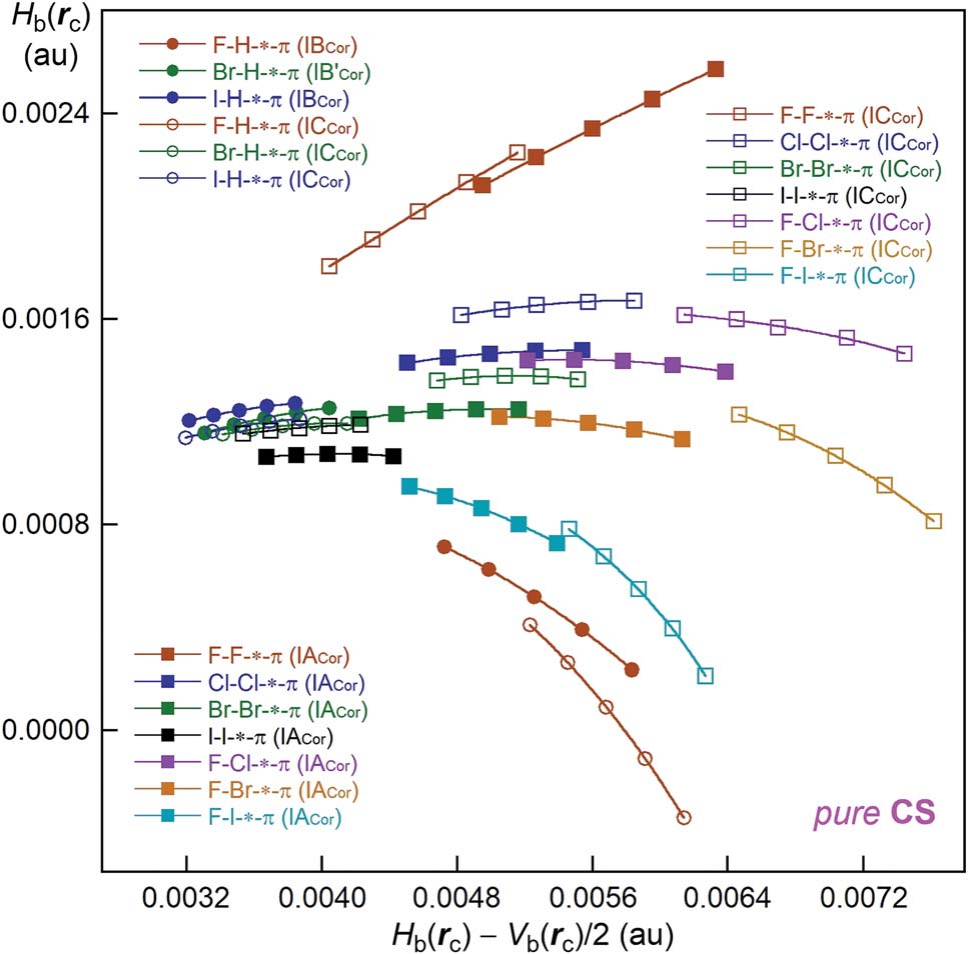
QTAIM-DFA plots of *H*_b_(*r*_c_) *versus H*_b_(*r*_c_) − *V*_b_(*r*_c_)/2 for X–H-*-π(C_24_H_12_) and Y–X-*-π(C_24_H_12_) (X, Y = F, Cl, Br and I). Marks and colours are shown in the figure, where circle and square marks correspond to the data evaluated with M06-2X/BSS-SA.

QTAIM functions are evaluated for the H-*-π and X-*- π interactions at BCPs in X–H-*-π(C_24_H_12_) and Y–X-*-π(C_24_H_12_) (X, Y = F, Cl, Br and I) using the M06-2X functional. The obtained values are presented in Table 2. Fig. 3 shows the plot of *H*_b_(*r*_c_) *versus H*_b_(*r*_c_) − *V*_b_(*r*_c_)/2 for the data in Table 2 and those from the perturbed structures around the fully optimized structures. All data in Fig. 3 appear in the region of *H*_b_(*r*_c_) − *V*_b_(*r*_c_)/2 > 0 and *H*_b_(*r*_c_) > 0, and therefore, all interactions in question are classified by the pure CS interactions.

**Table 2:** QTAIM functions and QTAIM-DFA parameters for X–H-*-π(C_24_H_12_) and Y–X-*-π(C_24_H_12_) (X, Y = F, Cl, Br and I), evaluated with M06-2X/BSS-SA and M06-2X/BSS-SB^a^^,^^b^

Y–X-*-π(C_24_H_12_), (symmetry: type)	*ρ*_b_(*r*_c_), (*ea*_o_^−3^)	*c*∇^2^*ρ*_b_(*r*_c_)^c^, (au)	*H*_b_(*r*_c_), (au)	*k*_b_(*r*_c_)^d^	*R*, (au)	*θ*, (°)	Freq, (cm^−1^)	*k*_f_, (mDyne Å^−1^)	*θ*_p_, (°)	*κ*_p_, (au^−1^)
**M06-2X/BSS-SA**										
F–H-*-π(^3^C) (*C*_1_: IB_Co_)^e^	0.0146	0.0053	0.0005	−0.948	0.0053	84.4	84.4	0.024	113.0	172.1
Br–H-*-π(^2^C) (*C*_1_: 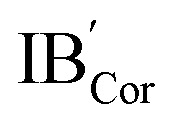 )	0.0090	0.0037	0.0012	−0.801	0.0039	71.6	60.4	0.025	82.4	162.5
I–H-*-π(^3^C) (*C*_1_: IB_Cor_)	0.0089	0.0035	0.0012	−0.785	0.0037	70.5	49.1	0.017	83.7	156.9
F–H-*-π(^12^M) (*C*_s_: IC_Cor_)	0.0172	0.0057	0.0001	−0.992	0.0057	89.1	134.4	0.059	129.1	242.5
Cl–H-*-π(^12^M) (*C*_1_: IC_Cor_)^e^^,^^f^	Non	Non	Non	Non	Non	Non	Non	Non	Non	Non
Br–H-*-π(^12^M) (*C*_s_: IC_Cor_)	0.0099	0.0038	0.0012	−0.814	0.0039	72.6	60.4	0.027	86.4	181.1
I–H-*-π(^12^M) (*C*_s_: IC_Cor_)	0.0093	0.0035	0.0012	−0.798	0.0037	71.4	53.5	0.020	83.7	171.6
F–F-*-π(^*a*^C) (*C*_s_: IA_Cor_)	0.0097	0.0056	0.0023	−0.735	0.0061	67.3	72.5	0.035	71.7	32.9
Cl–Cl-*-π(^*a*^C) (*C*_1_: IA_Cor_)^e^	0.0113	0.0050	0.0015	−0.828	0.0052	73.6	74.5	0.036	87.2	96.5
Br–Br-*-π(^*a*^C) (*C*_s_: IA_Cor_)	0.0115	0.0047	0.0012	−0.847	0.0048	75.1	59.2	0.024	87.9	121.0
I–I-*-π(^*a*^C) (*C*_s_: IA_Cor_)	0.0109	0.0040	0.0011	−0.846	0.0042	75.1	44.9	0.013	89.4	144.6
F–Cl-*-π(^*a*^C) (*C*_s_: IA_Cor_)	0.0131	0.0058	0.0014	−0.858	0.0060	76.0	79.5	0.037	92.0	108.4
F–Br-*-π(^*a*^C) (*C*_s_: IA_Cor_)	0.0137	0.0056	0.0012	−0.880	0.0057	77.9	67.4	0.033	94.5	135.3
F–I-*-π(^*a*^C) (*C*_s_: IA_Cor_)	0.0134	0.0049	0.0009	−0.904	0.0050	80.1	66.6	0.027	104.1	227.6
F–F-*-π(^12^M) (*C*_s_: IC_Cor_)	0.0081	0.0046	0.0020	−0.716	0.0050	66.2	61.2	0.026	68.1	24.5
Cl–Cl-*-π(^12^M) (*C*_1_: IC_Cor_)^e^	0.0124	0.0053	0.0017	−0.814	0.0055	72.6	170.6	0.070	86.4	98.1
Br–Br-*-π(^12^M) (*C*_1_: IC_Cor_)	0.0132	0.0051	0.0014	−0.843	0.0053	74.8	54.1	0.032	89.3	178.7
I–I-*-π(^12^M) (*C*_s_: IC_Cor_)	0.0108	0.0039	0.0012	−0.821	0.0040	73.1	48.1	0.012	87.1	107.5
F–Cl-*-π(^12^M) (*C*_1_: IC_Cor_)^e^	0.0163	0.0067	0.0016	−0.867	0.0069	76.8	73.8	0.023	95.8	92.0
F–Br-*-π(^12^M) (*C*_s_: IC_Cor_)	0.0189	0.0070	0.0011	−0.918	0.0071	81.4	108.7	0.042	109.6	220.8
F–I-*-π(^12^M) (*C*_s_: IC_Cor_)	0.0177	0.0059	0.0005	−0.951	0.0059	84.7	106.8	0.039	124.8	375.2

**M06-2X/BSS-SB**										
F–H-*-π(^3^C) (*C*_s_: IB_Cor_)	0.0142	0.0053	0.0014	−0.852	0.0055	75.6	115.5	0.051	78.7	68.4
Cl–H-*-π(^2^C) (*C*_1_: IA_Co_)^g^	0.0118	0.0041	0.0009	−0.870	0.0042	77.0	310.7	0.231	75.7	535
Br–H-*-π(^2^C) (*C*_1_: IB_Cor_)	0.0092	0.0033	0.0008	−0.871	0.0034	77.1	59.2	0.025	82.0	12.2
I–H-*-π(^3^C) (*C*_1_: IB_Cor_)	0.0093	0.0033	0.0009	−0.847	0.0034	75.2	49.5	0.019	84.7	81.0
Cl–H-*-π(^12^M) (*C*_1_: IC_Cor_)	0.0108	0.0036	0.0007	−0.895	0.0037	79.2	24.6	0.003	81.9	149.9

a See text for BSS-SA and BSS-SB.

b Data are given at BCP, which is shown by X-*-π.

c *c*∇^2^*ρ*_b_(*r*_c_) = *H*_b_(*r*_c_) − *V*_b_(*r*_c_)/2, where *c* = *ħ*^2^/8*m*.

d *k*_b_(*r*_c_) = *V*_b_(*r*_c_)/*G*_b_(*r*_c_).

e One imaginary frequency being predicted for each.

f BCP (and BP) being not detected.

g Perturbed structures for Cl–H-*-π(^2^C) (*C*_1_: IA_Cor_) are generated employing *w* = −0.1, −0.05, (0), 0.01 and 0.015 in eqn (2); therefore, some intervals in the plot are shorter than others.

### Nature of X–H-*-π(C_24_H_12_) and Y–X-*-π(C_24_H_12_) interactions, evaluated with M06-2X/BSS-SA

The plots of *H*_b_(*r*_c_) *versus H*_b_(*r*_c_) − *V*_b_(*r*_c_)/2 in Fig. 3 are analysed according to eqn (S3)–(S6) of the ESI,† which provide the QTAIM-DFA parameters of (*R*, *θ*) and (*θ*_p_, *κ*_p_). Table 2 collects the frequencies, correlated to NIV employed to generate the perturbed structures and the force constants, *k*_f_. The nature of the interactions in question is classified and characterized based on the QTAIM-DFA parameters, employing the standard values (criteria) as the reference. Table 3 summarizes the predicted nature of H-*-π in X–H-*-π(C_24_H_12_) and X-*-π in Y–X-*-π(C_24_H_12_), employing the *θ* and *θ*_p_ values evaluated with M06-2X/BSS-SA.

**Table 3:** Nature of the H-*-π and X-*-π interactions in X–H-*-π(C_24_H_12_) and Y–X-*-π(C_24_H_12_), respectively, evaluated with M06-2X/BSS-SA^a^

Y–X-*-π(C_24_H_12_), (symmetry: type)	*θ*, (°)	*θ*_p_, (°)	Predicted nature	Y–X-*-π(C_24_H_12_), (symmetry: type)	*θ*, (°)	*θ*_p_, (°)	Predicted nature
X–H-*-π(C_**24**_ **H**_**12**_ **)**							
F–H-*-π(^3^C) (*C*_1_: IB_Cor_)^b^	84.4	113.0	p-CS/t-HB_nc_^c^	F–H-*-π(^12^M) (*C*_s_: IC_Cor_)	89.1	129.1	p-CS/t-HB_nc_^c^^,^^d^
Br–H-*-π(^2^C) (*C*_1_: IB_Cor_)	71.6	82.4	p-CS/vdW^e^	Br–H-*-π(^12^M) (*C*_s_: IC_Cor_)	72.6	86.4	p-CS/vdW^e^
I–H-*-π(^3^C) (*C*_1_: IB_Cor_)	70.5	83.7	p-CS/vdW^e^	I–H-*-π(^12^M) (*C*_s_: IC_Cor_)	71.4	83.7	p-CS/vdW^e^

Y–X-*-π(C_**24**_ **H**_**12**_ **)**							
F–F-*-π(^*a*^C) (*C*_s_: IA_Cor_)	67.3	71.7	p-CS/vdW^e^	F–F-*-π(^12^M) (*C*_s_: IC_Cor_)	66.2	68.1	p-CS/vdW^e^
Cl–Cl-*-π(^*a*^C) (*C*_1_: IA_Cor_)^b^	73.6	87.2	p-CS/vdW^e^	Cl–Cl-*-π(^12^M) (*C*_1_: IC_Cor_)^b^	72.6	86.4	p-CS/vdW^e^
Br–Br-*-π(^*a*^C) (*C*_s_: IA_Cor_)	75.1	87.9	p-CS/vdW^e^	Br–Br-*-π(^12^M) (*C*_1_: IC_Cor_)	74.8	89.3	p-CS/vdW^e^
I–I-*-π(^*a*^C) (*C*_s_: IA_Cor_)	75.1	89.4	p-CS/vdW^e^	I–I-*-π(^12^M) (*C*_s_: IC_Cor_)	73.1	87.1	p-CS/vdW^e^
F–Cl-*-π(^*a*^C) (*C*_s_: IA_Cor_)	76.0	92.0	p-CS/t-HB_nc_^c^	F–Cl-*-π(^12^M) (*C*_1_: IC_Cor_)^b^	76.8	95.8	p-CS/t-HB_nc_^c^
F–Br-*-π(^*a*^C) (*C*_s_: IA_Cor_)	77.9	94.5	p-CS/t-HB_nc_^c^	F–Br-*-π(^12^M) (*C*_s_: IC_Cor_)	81.4	109.6	p-CS/t-HB_nc_^c^
F–I-*-π(^*a*^C) (*C*_s_: IA_Cor_)	80.1	104.1	p-CS/t-HB_nc_^c^	F–I-*-π(^12^M) (*C*_s_: IC_Cor_)	84.7	124.8	p-CS/t-HB_nc_^c^

a See text for BSS-SA.

b One imaginary frequency being predicted for each.

c Classified by the pure closed shell (CS) interactions and characterized as the typical hydrogen bonds (t-HB) with no covalency.

d Very close to the regular CS (r-CS) interactions and characterized as t-HB with covalency (t-HB_wc_).

e Predicted to be the vdW interactions appeared in the p-CS region.

As summarized in Table 3, the *θ* and *θ*_p_ values in X–H-*-π(C_24_H_12_) decrease in the order of X–H- = F–H- > Br–H- > I–H-, even though *θ*_p_ for X–H- = I–H- appears to be somewhat larger than that for the case of X–H- = Br–H-. The results show that *θ* and *θ*_p_ in X–H-*-π(C_24_H_12_) are controlled by the electronegativity of X. Namely, the values will be larger if the polarity of the X^δ−^–H^δ+^ type becomes larger. Conversely, *θ* and *θ*_p_ in Y–X-*-π(C_24_H_12_) become larger in the order of Y–X- = F–F- < Cl–Cl- < Br–Br- < I–I- < F–Cl- < F–Br- < F–I-. These results would be the reflection of two factors. The first is the softness of X. The *θ* and *θ*_p_ values become larger with increasing softness of X. The second factor is the polarity of Y^δ−^–X^δ+^. The *θ* and *θ*_p_ values increase with increasing polarity, resulting in the larger extension of σ*(X–Y) at the X side. This is very interesting because the *θ* and *θ*_p_ values are larger for Y–X- = F–Cl-, relative to the case of Y–X- = I–I-. The predicted nature is discussed next.

It would be instructive to review the criteria before the detailed discussion of the nature for H-*-π and X-*-π. The criteria specify that *θ* < 180° (*H*_b_(*r*_c_) − *V*_b_(*r*_c_)/2 > 0) for the CS interactions and *θ* > 180° (*H*_b_(*r*_c_) − *V*_b_(*r*_c_)/2 < 0) for the SS interactions. The CS interactions for *θ* < 180° are sub-divided into the pure CS interactions for 45° < *θ* < 90° (*H*_b_(*r*_c_) > 0) and the regular CS interactions for 90° < *θ* < 180° (*H*_b_(*r*_c_) < 0). The *θ*_p_ value plays an important role in characterizing the interactions. In the pure CS region of 45° < *θ* < 90°, the character of interactions will be the vdW type for 45° < *θ*_p_ < 90° and the typical-HB type (t-HB) with no covalency (t-HB_nc_) for 90° < *θ*_p_ < 125°, where *θ*_p_ = 125° is tentatively given, corresponding to *θ* = 90°. The regular CS (90° < *θ* < 180°) and SS (180° < *θ*) interactions are not discussed here, since the interactions in this region are not detected in this work.

The *θ* values are less than 90° for all X–H-*-π(C_24_H_12_) and Y–X-*-π(C_24_H_12_) interactions examined in this work. Therefore, the H-*-π and X-*-π interactions are all classified by the pure CS interactions. On the other hand, the *θ*_p_ values are less than 90° for all interactions with the exception of F–H-*-π(C_24_H_12_) of the IB_Cor_ and IC_Cor_ types and F–X-*-π(C_24_H_12_) (X = Cl, Br and I) of the IA_Cor_ and IC_Cor_ types. The interactions in X–H-*-π(C_24_H_12_) and Y–X-*-π(C_24_H_12_) are all characterized as the vdW nature for those with *θ*_p_ < 90°. The interactions with *θ*_p_ > 90° are characterized to have the nature of typical hydrogen bonds with no covalency (t-HB_nc_). However, the nature of the H-*-π interactions in F–H-*-π(^12^M) (*C*_s_: IC_Cor_) should be examined carefully. The *θ*_p_ value is 129.1°, which is larger than 125°. The results suggest that the H-*-π interaction should be characterized as t-HB with covalency (t-HB_wc_). However, the *θ* value of 89.1° is less than 90°, therefore, the interaction must have no covalency. In this case, the *θ* value should have the priority to the *θ*_p_ value in the prediction of the nature of the interaction, since *θ*_p_ is only given tentatively corresponding to *θ* = 90°. Therefore, the H-*-π interaction in F–H-*-π(^12^M) (*C*_s_: IC_Cor_) would be better characterized as t-HB_nc_. However, the interaction appears to be close to the borderline area between t-HB_nc_ and t-HB_wc_, since *θ* = 89.1° is close to 90°, while *θ*_p_ = 129.1° > 125°.

The X–H-*-π(C_24_H_12_) and Y–X-*-π(C_24_H_12_) interactions (X, Y = F, Cl, Br and I) were also analysed for the ID_Cor_ type with M06-2X/BSS-SA (see Scheme 2). The results of this analysis are discussed next.

### Nature of X–H-*-π(C_24_H_12_) and Y–X-*-π(C_24_H_12_) interactions of the ID_Cor_ type, evaluated with M06-2X/BSS-SA

Indeed, the ID_Cor_ type is not optimized for X–H-*-π(C_24_H_12_) with M06-2X/BSS-SA, even though they are optimized when calculated at the MP2 level. The nature of the Y–X-*-π(C_24_H_12_) interactions around the main axis of π(C_24_H_12_) is also very interesting. Therefore, X–H-*-π(C_24_H_12_) and Y–X-*-π(C_24_H_12_) are optimized assuming the *C*_2v_ symmetry. The structural parameters are presented in Table S4 of the ESI,† and are defined in Scheme 2. Table S5 of the ESI† presents the QTAIM-DFA parameters of (*R*, *θ*) and (*θ*_p_, *κ*_p_) evaluated with M06-2X/BSS-SA, together with the frequencies correlated to NIV employed to generate the perturbed structures and the force constants *k*_f_.

The nature of the interactions in question is classified and characterized based on the QTAIM-DFA parameters, employing the standard values as the reference. Table 4 summarizes the predicted nature of the H-*-π and X-*-π interactions in X–H-*-π(C_24_H_12_) and Y–X-*-π(C_24_H_12_) of the *C*_2v_ symmetry, respectively, employing the *θ* and *θ*_p_ values evaluated with M06-2X/BSS-SA. As summarized in Table 4, the *θ* and *θ*_p_ values in X–H-*-π(C_24_H_12_) (*C*_2v_: ID_Cor_) decrease in the order of X–H- = F–H- > Cl–H- > I–H- > Br–H-. On the other hand, the *θ*_p_ values in Y–X-*-π(C_24_H_12_) increase in the order of F–F- < Cl–Cl- < I–I- and F–Cl- < Br–Br- < F–Br- < F–I-. The *θ* and *θ*_p_ are smaller than 90° for all interactions in X–H-*-π(C_24_H_12_) (*C*_2v_: ID_Cor_) and Y–X-*-π(C_24_H_12_) (*C*_2v_: ID_Cor_) (see Table 4). Therefore, the H-*-π and X-*-π interactions are all classified by the pure CS interactions and are characterized to be of the vdW nature (p-CS/vdW).

**Table 4:** Nature of the H-*-π and X-*-π interactions in X–H-*-π(C_24_H_12_) and Y–X-*-π(C_24_H_12_) of the *C*_2v_ symmetry, respectively, evaluated with M06-2X/BSS-SA^a^

Y–X-*-π(C_24_H_12_), (symmetry: type)	*θ*, (°)	*θ*_p_, (°)	Predicted nature	Y–X-*-π(C_24_H_12_), (symmetry: type)	*θ*, (°)	*θ*_p_, (°)	Predicted nature
F–H-*-π(M_o_) (*C*_2v_: ID_Cor_)	70.4	79.5	p-CS/vdW^b^	Cl–H-*-π(M_o_) (*C*_2v_: ID_Cor_)	69.7	75.5	p-CS/vdW^b^
Br–H-*-π(M_o_) (*C*_2v_: ID_Cor_)	69.5	76.1	p-CS/vdW^b^	I–H-*-π(M_o_) (*C*_2v_: ID_Cor_)	69.6	76.5	p-CS/vdW^b^
F–F-*-π(M_o_) (*C*_2v_: ID_Cor_)	65.9	66.6	p-CS/vdW^b^				
Cl–Cl-*-π(M_o_) (*C*_2v_: ID_Cor_)	66.7	74.4	p-CS/vdW^b^	F–Cl-*-π(M_o_) (*C*_2v_: ID_Cor_)	67.4	75.1	p-CS/vdW^b^
Br–Br-*-π(M_o_) (*C*_2v_: ID_Cor_)	69.0	76.0	p-CS/vdW^b^	F–Br-*-π(M_o_) (*C*_2v_: ID_Cor_)	69.9	76.1	p-CS/vdW^b^
I–I-*-π(M_o_) (*C*_2v_: ID_Cor_)	69.1	75.1	p-CS/vdW^b^	F–I-*-π(M_o_) (*C*_2v_: ID_Cor_)	70.6	77.3	p-CS/vdW^b^

a See text for BSS-SA.

b Classified by the p-CS interactions and characterized as the vdW interactions.

### Nature of X–H-*-π(C_24_H_12_) and Y–X-*-π(C_24_H_12_) *versus* that of X–H-*-π(C_6_H_6_) and Y–X-*-π(C_6_H_6_)

**Fig. 4:**
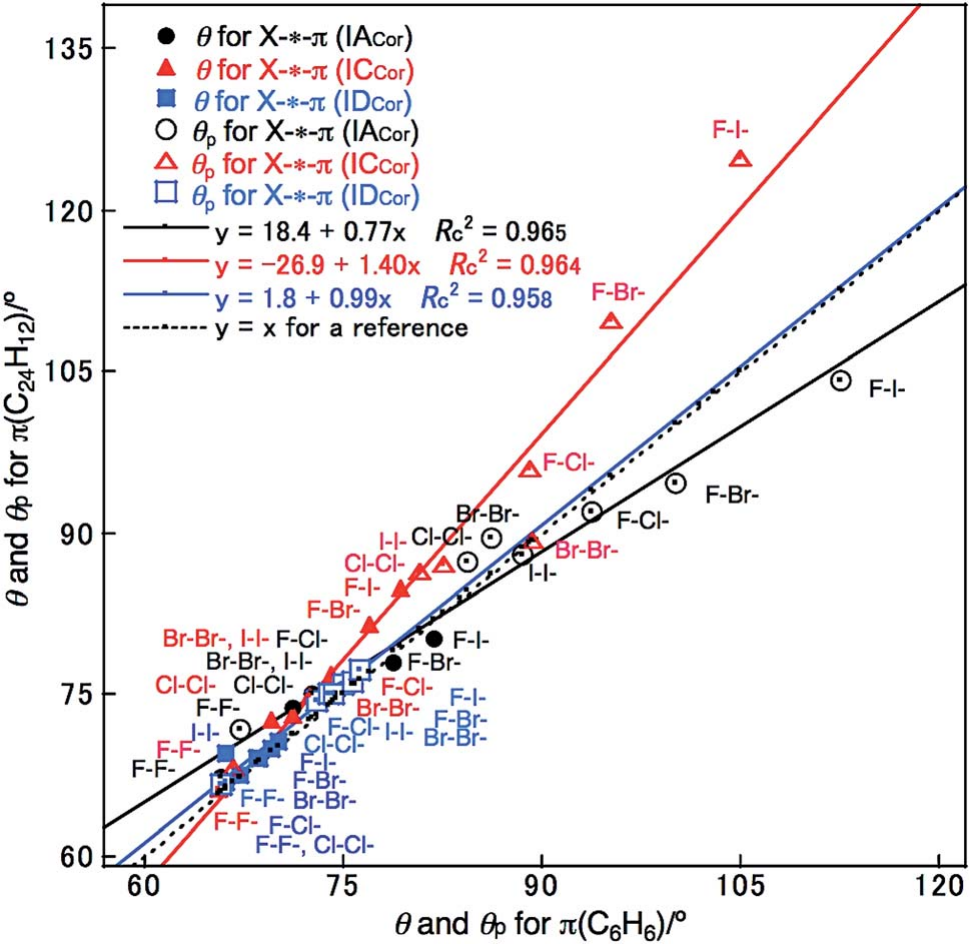
Plots of *θ* and *θ*_p_ for Y–X-*-π(C_24_H_12_) (X, Y = F, Cl, Br and I) *versus* those for Y–X-*-π(C_6_H_6_), respectively, evaluated with M06-2X/BSS-SA.

The Y–X-*-π(C_6_H_6_) and X–H-*-π(C_6_H_6_) interactions (X, Y = F, Cl, Br and I) are similarly evaluated with M06-2X/BSS-SA. The results are presented in Table S6 and S7 of the ESI.†Fig. 4 shows the plots of *θ* and *θ*_p_ for Y–X-*-π(C_24_H_12_) *versus* those of Y–X-*-π(C_6_H_6_) for convenience of comparison. As shown in Fig. 4, the *θ* and *θ*_p_ values for Y–X-*-π(^*a*^C: C_24_H_12_) (IA_Cor_) appear to be somewhat smaller than those for Y–X-*-π(C_6_H_6_) (*C*_s_: IB_Bzn_), respectively, if those of the same Y–X are compared, whereas the values for Y–X-*-π(^12^M: C_24_H_12_) (IC_Cor_) are predicted to be larger than those for Y–X-*-π(C_6_H_6_) (*C*_s_: IB_Bzn_), respectively. Conversely, the *θ* and *θ*_p_ values for Y–X-*-π(M_o_: C_24_H_12_) (*C*_2v_: ID_Cor_) are very close to those for Y–X-*-π(C_6_H_6_) (*C*_2v_: ID_Bzn_), respectively, if those of the same Y–X are compared.

**Scheme 3:**
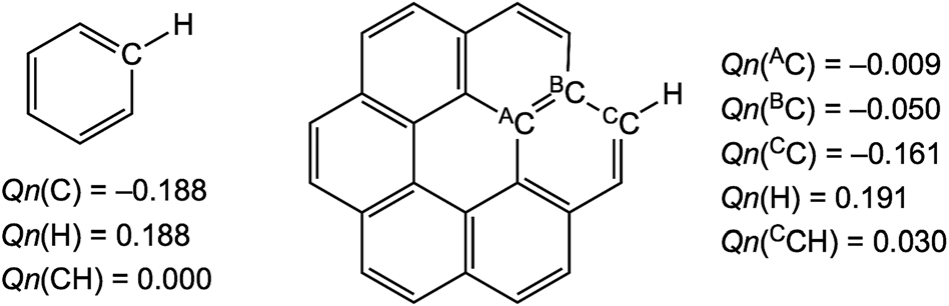
Natural charges (*Q*_n_) on the C and H atoms in benzene and coronene evaluated with MP2/6-311G(d,p).

What is the reason for the predicted results shown in Fig. 4? The charge developed on the C and H atoms of benzene and coronene is examined as the possible origin of these results. Scheme 3 shows the charge evaluated based on the natural population analysis (*Q*_n_) with MP2/6-311G(d,p).^57^ The outside ^C^C–H bonds in coronene are predicted to be substantially positively charged relative to the case of benzene, and the inside ^A^C_6_ atoms are almost neutral, resulting in the negative charge accumulated on the ^B^C atoms (see, Scheme 3). The results show that the *θ* and *θ*_p_ values for Y–X-*-π(C_24_H_12_) would be larger than those for Y–X-*-π(C_6_H_6_), respectively, if *Q*_n_ for the former or around the interaction is smaller than for the latter. For the small range of the interactions in the adducts, the electron–electron repulsion may play a more important role in the strength of the X-*-π interaction rather than the attractive interaction such as the CT interaction.

The *θ* and *θ*_p_ values for X–H-*-π(C_24_H_12_) are similarly plotted *versus* those for X–H-*-π(C_6_H_6_), as shown in Fig. S9 of the ESI.† In this case, the *θ* and *θ*_p_ values increase in the order of π(M_o_: C_24_H_12_) (ID_Cor_) < π(C_6_H_6_) ≤ π(^3^C: C_24_H_12_) (IB_Cor_) < π(^12^M: C_24_H_12_) (IC_Cor_), if those of the same X–H are compared. These results appear to be in close agreement to those for Y–X-*-π(C_24_H_12_) with Y–X-*-π(C_6_H_6_) (see, Fig. S9 of the ESI†), even though small differences between the two cases are observed.

The H-*-π and X-*-π interactions in the bent π-systems are also of highly interest. An investigation of such interactions is currently in progress.

## Conclusions

QTAIM-DFA was applied to the X–H-*-π(C_24_H_12_) (X = F, Cl, Br and I) and Y–X-*-π(C_24_H_12_) (Y–X = F–F, Cl–Cl, Br–Br, I–I, F–Cl, F–Br and F–I) interactions, which must be of fundamental importance. The structures were optimized mainly at the M06-2X/BSS-SA level of theory. Four types of structures were optimized for X–H⋯π(C_24_H_12_) and Y–X⋯π (C_24_H_12_) (types IA_Cor_, IB_Cor_, IC_Cor_ and ID_Cor_) (see, Schemes 1 and 2). The IB_Cor_ and IC_Cor_ types were predicted for X–H⋯π(C_24_H_12_), while the IA_Cor_ and IC_Cor_ types were for Y–X⋯π(C_24_H_12_), if optimized with M06-2X/BSS-SA. All BCPs expected are clearly observed in the molecular graphs drawn on the optimized structures.

QTAIM-DFA parameters of (*R*, *θ*) and (*θ*_p_, *κ*_p_) are calculated for H-*-π in X–H-*-π(C_24_H_12_) and X-*-π in Y–X-*-π(C_24_H_12_) by analysing the plots of *H*_b_(*r*_c_) *versus H*_b_(*r*_c_) − *V*_b_(*r*_c_)/2 at BCPs. The *θ* values are smaller than 90° for all X–H-*-π(C_24_H_12_) and Y–X-*-π(C_24_H_12_) interactions, and are therefore classified as the pure CS interactions. The *θ*_p_ values are larger than 90° for F–H-*-π(C_24_H_12_) of the IB_Cor_ and IC_Cor_ types and F–X-*-π(C_24_H_12_) (X = Cl, Br and I) of the IA_Cor_ and IC_Cor_ types; therefore, they have the t-HB_nc_ nature. The H-*-π interaction in F–H-*-π(C_24_H_12_) (*C*_s_: type IC_Cor_) appear to be present close to the borderline area between t-HB_nc_ and t-HB_wc_, since *θ* = 89.1°, which is close to 90°, while *θ*_p_ = 129.1° > 125°. The H-*-π and X-*-π interactions other than above have the vdW nature due to *θ*_p_ < 90°. The *θ* and *θ*_p_ values are smaller than 90° for all interactions in question in X–H-*-π(C_24_H_12_) (*C*_2v_: ID_Cor_) and Y–X-*-π(C_24_H_12_) (*C*_2v_: ID_Cor_). Therefore, the H-*-π and X-*-π interactions around the main axis of π(C_24_H_12_) in the adducts are all predicted to have the nature of p-CS/vdW. The *θ* and *θ*_p_ values for Y–X-*-π(M_o_: C_24_H_12_) (*C*_2v_: ID_Cor_) are very close to the corresponding values for Y–X-*-π(C_6_H_6_) (*C*_2v_: ID_Bzn_), respectively. Conversely, the *θ* and *θ*_p_ values for Y–X-*-π(^*a*^C: C_24_H_12_) (IA_Cor_) appear to be somewhat smaller than the corresponding values for Y–X-*-π(C_6_H_6_) (*C*_s_: IB_Bzn_), respectively, whereas the values for Y–X-*-π(^12^M: C_24_H_12_) (IC_Cor_) are predicted to be larger than those for Y–X-*-π(C_6_H_6_) (*C*_s_: IB_Bzn_), respectively.

## Conflicts of interest

The authors declare no conflict of interest.

## Supplementary Material

RA-008-C8RA01862F-s001
