# Supplementary material for: Behaviour of the XH-*-π and YX-*-π interactions (X, Y = F, Cl, Br and I) in the coronene π-system, as elucidated by QTAIM dual functional analysis with QC calculations
Source: RSC Adv. 2018 May 3;8(29):16349–61. doi: 10.1039/c8ra01862f (PMC9080335; doi:10.1039/c8ra01862f)
Supplement: RA-008-C8RA01862F-s001 [file RA-008-C8RA01862F-s001.pdf]

Electronic Supplementary Material (ESI) for RSC Advances.
This journal is © The Royal Society of Chemistry 2018

Electronic Supplementary Information

Behaviour of the XH-∗-π and YX-∗-π interactions (X, Y = F, Cl, Br and I) in the coronene π-system, as elucidated by QTAIM dual functional analysis with QC calculations

Satoko Hayashi,* Yuji Sugibayashi and Waro Nakanishi*
Faculty of Systems Engineering, Wakayama University, 930 Sakaedani, Wakayama, 640-8510 Japan
E-mail: hayashi3@sys.wakayama-u.ac.jp and nakanisi@sys.wakayama-u.ac.jp

QTAIM Dual Functional Analysis (QTAIM-DFA)

The bond critical point (BCP; ∗) is an important concept in QTAIM. The BCP of $(\omega, \sigma) = (3, -1)$[S1] is a point along the bond path (BP) at the interatomic surface, where charge density $\rho(\boldsymbol{r})$ reaches a minimum. It is donated by $\rho_b(\boldsymbol{r}_c)$. While the chemical bonds or interactions between A and B are denoted by A–B, which correspond to BPs between A and B in QTAIM, A-∗-B emphasizes the presence of BCP (∗) in A–B.

The sign of the Laplacian $\rho_b(\boldsymbol{r}_c)$ ($\nabla^2\rho_b(\boldsymbol{r}_c)$) indicates that $\rho_b(\boldsymbol{r}_c)$ is depleted or concentrated with respect to its surrounding, since $\nabla^2\rho_b(\boldsymbol{r}_c)$ is the second derivative of $\rho_b(\boldsymbol{r}_c)$. $\rho_b(\boldsymbol{r}_c)$ is locally depleted relative to the average distribution around $\boldsymbol{r}_c$ if $\nabla^2\rho_b(\boldsymbol{r}_c) > 0$, but it is concentrated when $\nabla^2\rho_b(\boldsymbol{r}_c) < 0$. Total electron energy densities at BCPs ($H_b(\boldsymbol{r}_c)$) must be a more appropriate measure for weak interactions on the energy basis.[S1–S6] $H_b(\boldsymbol{r}_c)$ are the sum of kinetic energy densities ($G_b(\boldsymbol{r}_c)$) and potential energy densities ($V_b(\boldsymbol{r}_c)$) at BCPs, as shown in eqn (S1). Electrons at BCPs are stabilized when $H_b(\boldsymbol{r}_c) < 0$, therefore, interactions exhibit the covalent nature in this region, whereas they exhibit no covalency if $H_b(\boldsymbol{r}_c) > 0$, due to the destabilization of electrons at BCPs under the conditions.[S1] Eqn (S2) represents the relation between $\nabla^2\rho_b(\boldsymbol{r}_c)$ and $H_b(\boldsymbol{r}_c)$, together with $G_b(\boldsymbol{r}_c)$ and $V_b(\boldsymbol{r}_c)$, which is closely related to the virial theorem.

$$H_b(\boldsymbol{r}_c) = G_b(\boldsymbol{r}_c) + V_b(\boldsymbol{r}_c) \quad \text{(S1)}$$
$$(\hbar^2/8m)\nabla^2\rho_b(\boldsymbol{r}_c) = H_b(\boldsymbol{r}_c) - V_b(\boldsymbol{r}_c)/2 \quad \text{(S2)}$$
$$= G_b(\boldsymbol{r}_c) + V_b(\boldsymbol{r}_c)/2 \quad \text{(S2')}$$

Interactions are classified by the signs of $\nabla^2\rho_b(\boldsymbol{r}_c)$ and $H_b(\boldsymbol{r}_c)$. Interactions in the region of $\nabla^2\rho_b(\boldsymbol{r}_c) < 0$ are called shared-shell (SS) interactions and they are closed-shell (CS) interactions for $\nabla^2\rho_b(\boldsymbol{r}_c) > 0$. $H_b(\boldsymbol{r}_c)$ must be negative when $\nabla^2\rho_b(\boldsymbol{r}_c) < 0$, since $H_b(\boldsymbol{r}_c)$ are larger than $(\hbar^2/8m)\nabla^2\rho_b(\boldsymbol{r}_c)$ by $V_b(\boldsymbol{r}_c)/2$ with negative $V_b(\boldsymbol{r}_c)$ at all BCPs (eqn (S2)). Consequently, $\nabla^2\rho_b(\boldsymbol{r}_c) < 0$ and $H_b(\boldsymbol{r}_c) < 0$ for the SS interactions. The CS interactions are especially called *pure* CS interactions for $H_b(\boldsymbol{r}_c) > 0$ and $\nabla^2\rho_b(\boldsymbol{r}_c) > 0$, since electrons at BCPs are depleted and destabilized under the conditions.[S1a] Electrons in the intermediate

region between SS and *pure* CS, which belong to CS, are locally depleted but stabilized at BCPs, since $\nabla^2\rho_b(\boldsymbol{r}_c) > 0$ but $H_b(\boldsymbol{r}_c) < 0$.[S1a] We call the interactions in this region *regular* CS,[S4,S5] when it is necessary to distinguish from *pure* CS. The role of $\nabla^2\rho_b(\boldsymbol{r}_c)$ in the classification can be replaced by $H_b(\boldsymbol{r}_c) - V_b(\boldsymbol{r}_c)/2$, since $(\hbar^2/8m)\nabla^2\rho_b(\boldsymbol{r}_c) = H_b(\boldsymbol{r}_c) - V_b(\boldsymbol{r}_c)/2$ (eqn (S2)).

We proposed QTAIM-DFA by plotting $H_b(\boldsymbol{r}_c)$ versus $H_b(\boldsymbol{r}_c) - V_b(\boldsymbol{r}_c)/2$ (= $(\hbar^2/8m)\nabla^2\rho_b(\boldsymbol{r}_c)$),[S4a] after the proposal of $H_b(\boldsymbol{r}_c)$ versus $\nabla^2\rho_b(\boldsymbol{r}_c)$.[S4b] Both axes in the plot of the former are given in energy unit, therefore, distances on the (x, y) (= $(H_b(\boldsymbol{r}_c) - V_b(\boldsymbol{r}_c)/2, H_b(\boldsymbol{r}_c))$) plane can be expressed in the energy unit, which provides an analytical development. QTAIM-DFA can incorporate the classification of interactions by the signs of $\nabla^2\rho_b(\boldsymbol{r}_c)$ and $H_b(\boldsymbol{r}_c)$. Scheme S1 summarizes the QTAIM-DFA treatment. Interactions of *pure* CS appear in the first quadrant, those of *regular* CS in the fourth quadrant and SS interactions do in the third quadrant. No interactions appear in the second one.

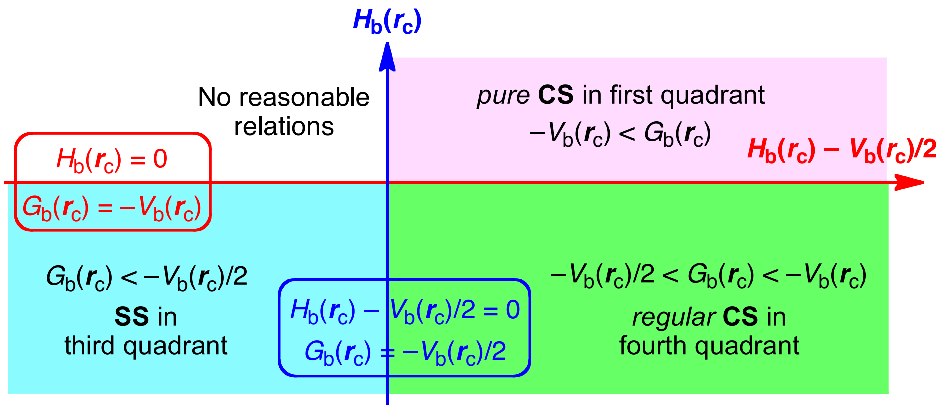

Scheme S1. QTAIM-DFA: Plot of $H_b(\boldsymbol{r}_c)$ versus $H_b(\boldsymbol{r}_c) - V_b(\boldsymbol{r}_c)/2$ for Weak to Strong Interactions

In our treatment, data for perturbed structures around fully optimized structures are also employed for the plots, together with the fully optimized ones (see Fig. S1).[S4–S6] We proposed the concept of the "dynamic nature of interaction" originated from the perturbed structures. The behavior of interactions at the fully optimized structures corresponds to "the static nature of interactions", whereas that containing perturbed structures exhibit the "dynamic nature of interaction" as explained below. The method to generate the perturbed structures is discussed later. Plots of $H_b(\boldsymbol{r}_c)$ versus $H_b(\boldsymbol{r}_c) - V_b(\boldsymbol{r}_c)/2$ are analyzed employing the polar coordinate (R, θ) representation with (θ_p, κ_p) parameters.[S4a,S5,S6] Fig. S1 explains the treatment. R in (R, θ) is defined by eqn (S3) and given in the energy unit. R corresponds to the energy for an interaction at BCP. The plots show a spiral stream, as a whole. θ in (R, θ) defined by eqn (S4), measured from the y-axis, controls the spiral stream of the plot. Each plot for an interaction shows a specific curve, which provides important information of the interaction (see Fig. S1). The curve is expressed by θ_p and κ_p. While θ_p, defined by eqn (S5) and measured from the y-direction, corresponds to the tangent line of a plot, where θ_p is calculated employing data of the perturbed structures with a fully-optimized structure and κ_p is the curvature of the plot (eqn (S6)). While (R, θ) correspond to the static nature, (θ_p, κ_p) represent the dynamic nature of interactions. We call (R, θ) and (θ_p, κ_p) QTAIM-DFA parameters, whereas $\rho_b(\boldsymbol{r}_c)$, $\nabla^2\rho_b(\boldsymbol{r}_c)$, $G_b(\boldsymbol{r}_c)$, $V_b(\boldsymbol{r}_c)$, $H_b(\boldsymbol{r}_c)$ and $H_b(\boldsymbol{r}_c) - V_b(\boldsymbol{r}_c)/2$ belong to

QTAIM functions. $k_b(\boldsymbol{r}_c)$, defined by eqn (S7), is an QTAIM function but it will be treated as if it were an QTAIM-DFA parameter, if suitable.

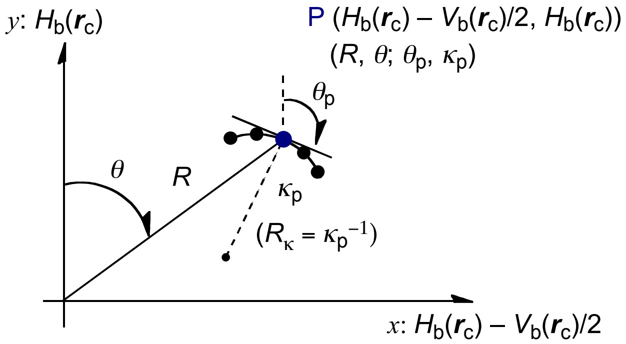

Fig. S1 Polar (R, θ) coordinate representation of $H_b(\boldsymbol{r}_c)$ versus $H_b(\boldsymbol{r}_c) - V_b(\boldsymbol{r}_c)/2$, with ($\theta_p$, κ_p) parameters.

$$R = (x^2 + y^2)^{1/2} \quad \text{(S3)}$$
$$\theta = 90° - \tan^{-1}(y/x) \quad \text{(S4)}$$
$$\theta_p = 90° - \tan^{-1}(dy/dx) \quad \text{(S5)}$$
$$\kappa_p = |d^2y/dx^2| / [1 + (dy/dx)^2]^{3/2} \quad \text{(S6)}$$
$$k_b(\boldsymbol{r}_c) = V_b(\boldsymbol{r}_c)/G_b(\boldsymbol{r}_c) \quad \text{(S7)}$$

where $(x, y) = (H_b(\boldsymbol{r}_c) - V_b(\boldsymbol{r}_c)/2, H_b(\boldsymbol{r}_c))$

Criteria for Classification of Interactions: Behavior of Typical Interactions Elucidated by QTAIM-DFA

$H_b(\boldsymbol{r}_c)$ are plotted versus $H_b(\boldsymbol{r}_c) - V_b(\boldsymbol{r}_c)/2$ for typical interactions in vdW (van der Waals interactions), HB (hydrogen bonds), CT-MC (molecular complexes through charge transfer), X_3^- (trihalide ions), CT-TBP (trigonal bipyramidal adducts through charge-transfer), Cov-w (weak covalent bonds) and Cov-s (strong covalent bonds).[S4–S6] Rough criteria are obtained, after the analysis of the plots for the typical interactions according to eqns (S3)–(S7), by applying QTAIM-DFA. Scheme S2 shows the rough criteria, which are accomplished by the θ and θ_p values, together with the values of $k_b(\boldsymbol{r}_c)$. The criteria will be employed to discuss the nature of interactions in question, as a reference.

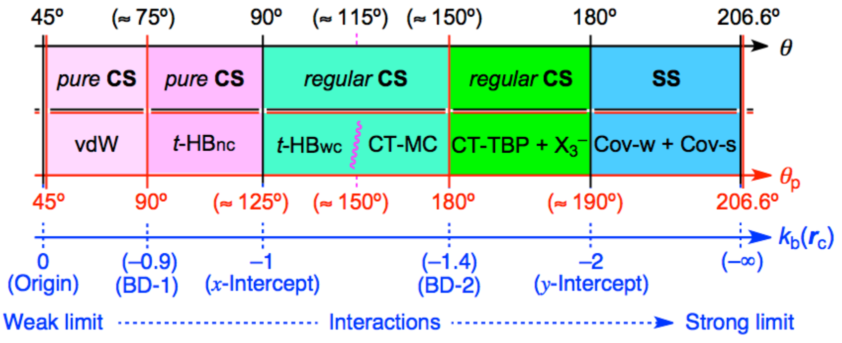

Scheme S2. Rough classification of interactions by θ and θ_p, together with $k_b(\boldsymbol{r}_c)$ (= $V_b(\boldsymbol{r}_c)/G_b(\boldsymbol{r}_c)$).

Table S1. Structural parameters for X–H---π($C_{24}H_{12}$), optimized with M06-2X/BSS-SB[a,b]

X–H-∗-π($C_{24}H_{12}$) (symmetry: type)	r_1 (Å)	r_2 (Å)	θ_1 (°)	θ_2 (°)	ϕ_1 (°)	ϕ_2 (°)	ΔE_{ES}[c,d] (kJ mol^{-1})	ΔE_{Ent}[c,e] (kJ mol^{-1})
F–H---π(^{3}C) (C_s: IB_{Cor})	2.2405	0.9286	83.39	170.58	-89.92	180.00	-19.7	-15.4
Cl–H---π(^{a}C) (C_s: IA_{Cor})	2.4625	1.2954	106.56	152.39	-97.70	0.75	-17.1	-11.1
Br–H---π(^{2}C) (C_1: IB_{Cor})	2.5885	1.4321	77.45	171.11	-111.88	47.86	-18.1	-11.3
I–H---π(^{3}C) (C_1: IB_{Cor})	2.6212	1.6272	76.25	163.81	-106.37	-21.93	-18.0	-12.0
Cl–H---π(^{12}M) (C_1: IC_{Cor})[f]	2.4450	1.2962	70.00	178.52	-90.00	0.00	-16.9	-15.8

[a] See text for BSS-SB. [b] See Scheme 2 of the text for the definition of the structural parameters. [c] $\Delta E = E$(X–H---π($C_{24}H_{12}$)/Y–X---π($C_{24}H_{12}$)) – (E(X–H/Y–X) + E($C_{24}H_{12}$)). [d] ΔE_{ES} stands for ΔE on the energy surface. [e] ΔE_{Ent} stands for ΔE with the correction of the heat of enthalpy. [f] One imaginary frequency being predicted for each.

Table S2. QTAIM functions and QTAIM-DFA parameters for X–H-∗-π($C_{24}H_{12}$) (X, Y = F, Cl, Br and I), evaluated with M06-2X/BSS-SB[a,b]

X–H-∗-π($C_{24}H_{12}$) (symmetry: type)	$\rho_b(\mathbf{r}_c)$ (ea_o^{-3})	$c\nabla^2\rho_b(\mathbf{r}_c)$[c] (au)	$H_b(\mathbf{r}_c)$ (au)	$k_b(\mathbf{r}_c)$[d]	R (au)	θ (°)	freq (cm^{-1})	k_f (mDyne Å^{-1})	θ_p (°)	κ_p (au^{-1})
F–H---π(^{3}C) (C_s: IB_{Cor})	0.0142	0.0053	0.0014	-0.852	0.0055	75.6	115.5	0.051	78.7	68.4
Cl–H---π(^{a}C) (C_s: IA_{Cor})[e]	0.0118	0.0041	0.0009	-0.870	0.0042	77.0	310.7	0.231	75.7	535
Br–H---π(^{2}C) (C_1: IB_{Cor})	0.0092	0.0033	0.0008	-0.871	0.0034	77.1	59.2	0.025	82.0	12.2
I–H---π(^{3}C) (C_1: IB_{Cor})	0.0093	0.0033	0.0009	-0.847	0.0034	75.2	49.5	0.019	84.7	81.0
Cl–H---π(^{12}M) (C_1: IC_{Cor})[f]	0.0108	0.0036	0.0007	-0.895	0.0037	79.2	24.6	0.003	81.9	149.9

[a] See text for BSS-SB. [b] Data are given at BCP, which is shown by X-∗-π. [c] $c\nabla^2\rho_b(\mathbf{r}_c) = H_b(\mathbf{r}_c) - V_b(\mathbf{r}_c)/2$, where $c = \hbar^2/8m$. [d] $k_b(\mathbf{r}_c) = V_b(\mathbf{r}_c)/G_b(\mathbf{r}_c)$. [e] Cl–H-∗-π(^{2}C) ($C_1$: IA_{Cor}) is generated employing w = –0.1, –0.05, (0), 0.01 and 0.015 in eqn (2); therefore, some intervals in the plot are shorter than others.[f] One imaginary frequency being predicted for each.

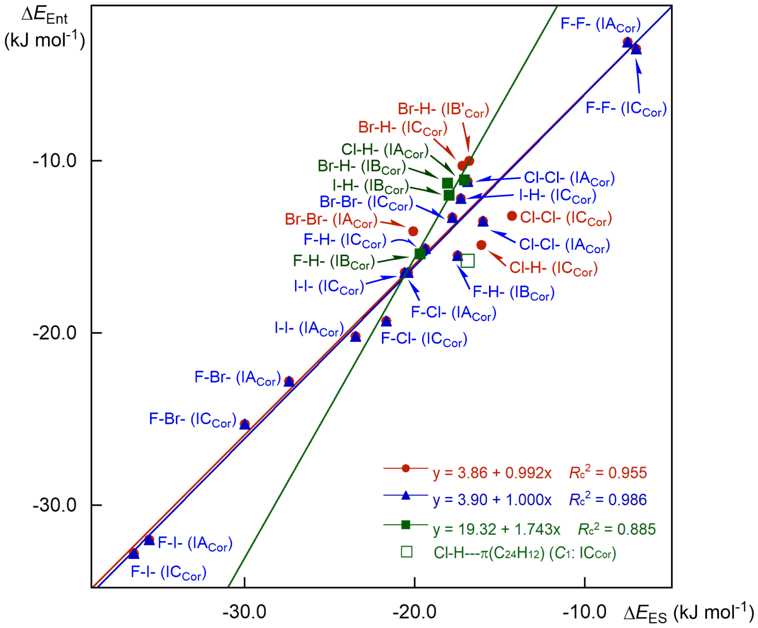

Fig. S2 Plots of ΔE_{Ent} versus ΔE_{ES} for X–H-∗-π($C_{24}H_{12}$) and Y–X-∗-π($C_{24}H_{12}$) (X, Y = F, Cl, Br and I) evaluated with M06-2X/BSS-SA.

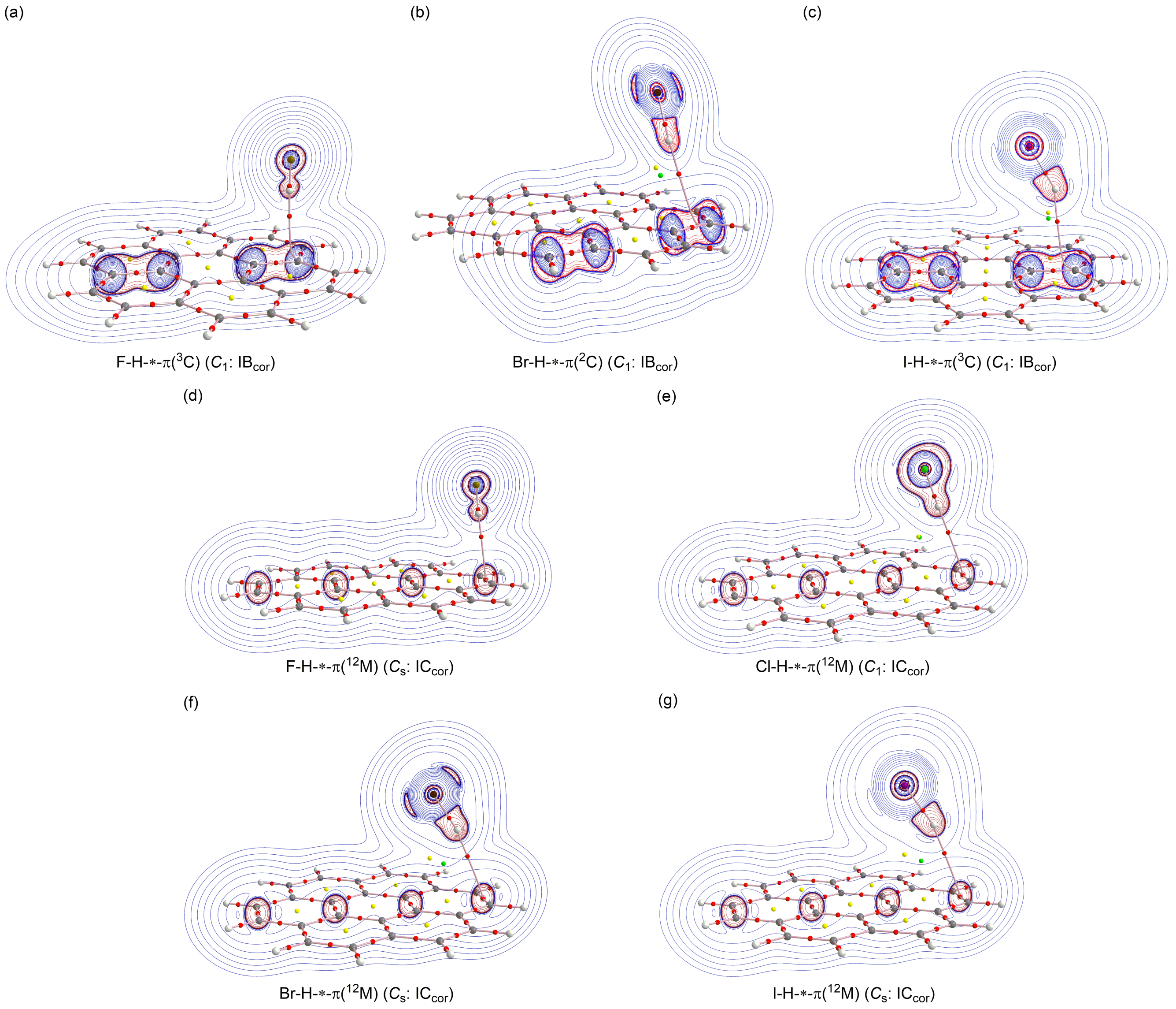

Fig. S3 Negative Laplacian for F–H-∗-π($C_{24}H_{12}$) (C_1: IB_{cor}) (a), Br–H-∗-π($C_{24}H_{12}$) (C_1: IB_{cor}) (b), I–H-∗-π($C_{24}H_{12}$) (C_1: IB_{cor}) (c), F–H-∗-π($C_{24}H_{12}$) (C_s: IC_{cor}) (d), Cl–H-∗-π($C_{24}H_{12}$) (C_1: IC_{cor}) (e), Br–H-∗-π($C_{24}H_{12}$) (C_s: IC_{cor}) (f) and I–H-∗-π($C_{24}H_{12}$) (C_s: IC_{cor}) (g), drawn similarly to the case Fig. 1 in the text with M06-2X/BSS-SA. Blue and red lines correspond to the positive and negative values, respectively.

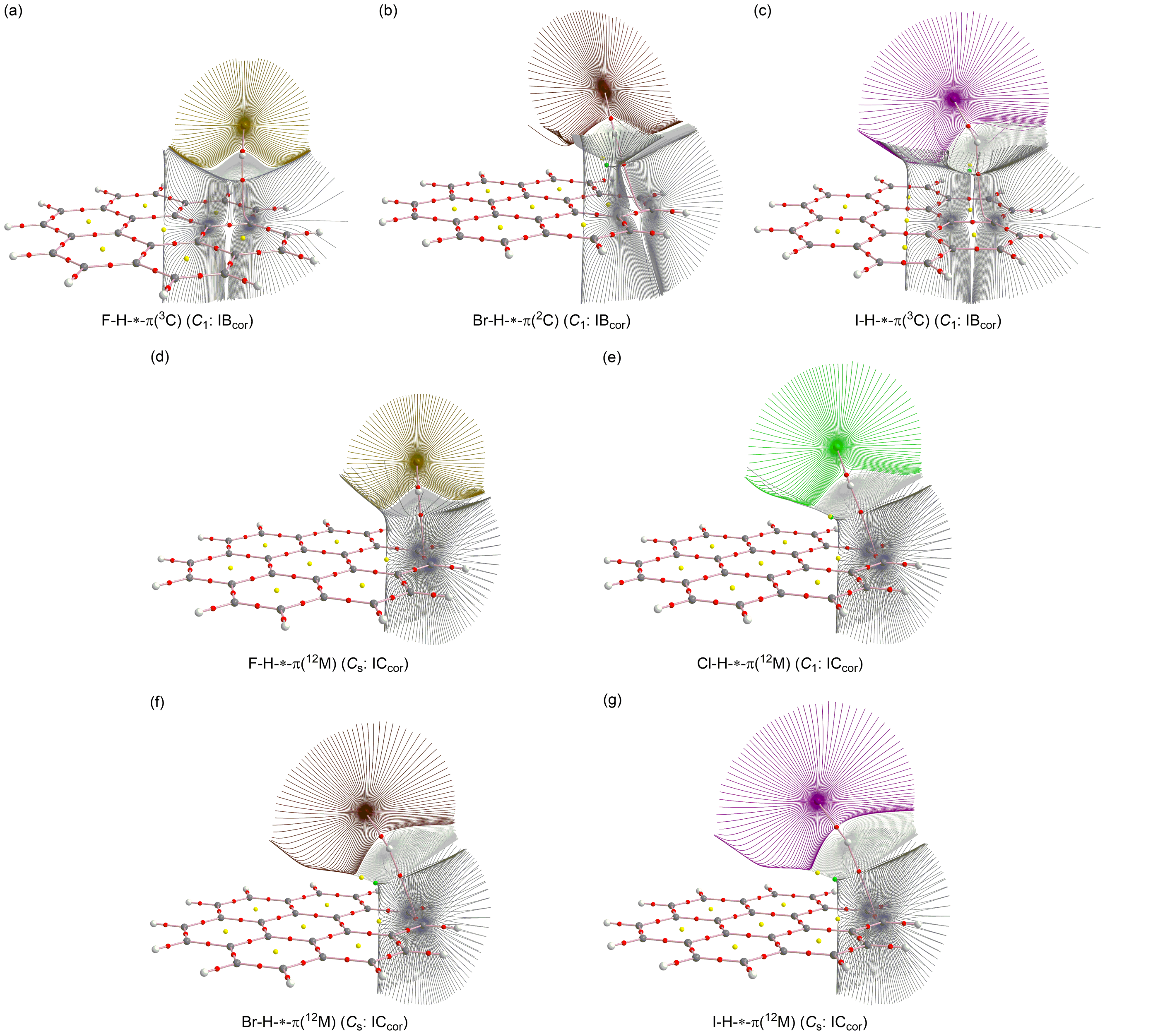

Fig. S4 Trajectory plots for F–H-∗-π($C_{24}H_{12}$) (C_1: IB_{cor}) (a), Br–H-∗-π($C_{24}H_{12}$) (C_1: IB_{cor}) (b), I–H-∗-π($C_{24}H_{12}$) (C_1: IB_{cor}) (c), F–H-∗-π($C_{24}H_{12}$) (C_s: IC_{cor}) (d), Cl–H-∗-π($C_{24}H_{12}$) (C_1: IC_{cor}) (e), Br–H-∗-π($C_{24}H_{12}$) (C_s: IC_{cor}) (f) and I–H-∗-π($C_{24}H_{12}$) (C_s: IC_{cor}) (g), drawn similarly to case of Fig. 1 in the text with M06-2X/BSS-SA. Colors and marks are the same as those in Fig. 1.

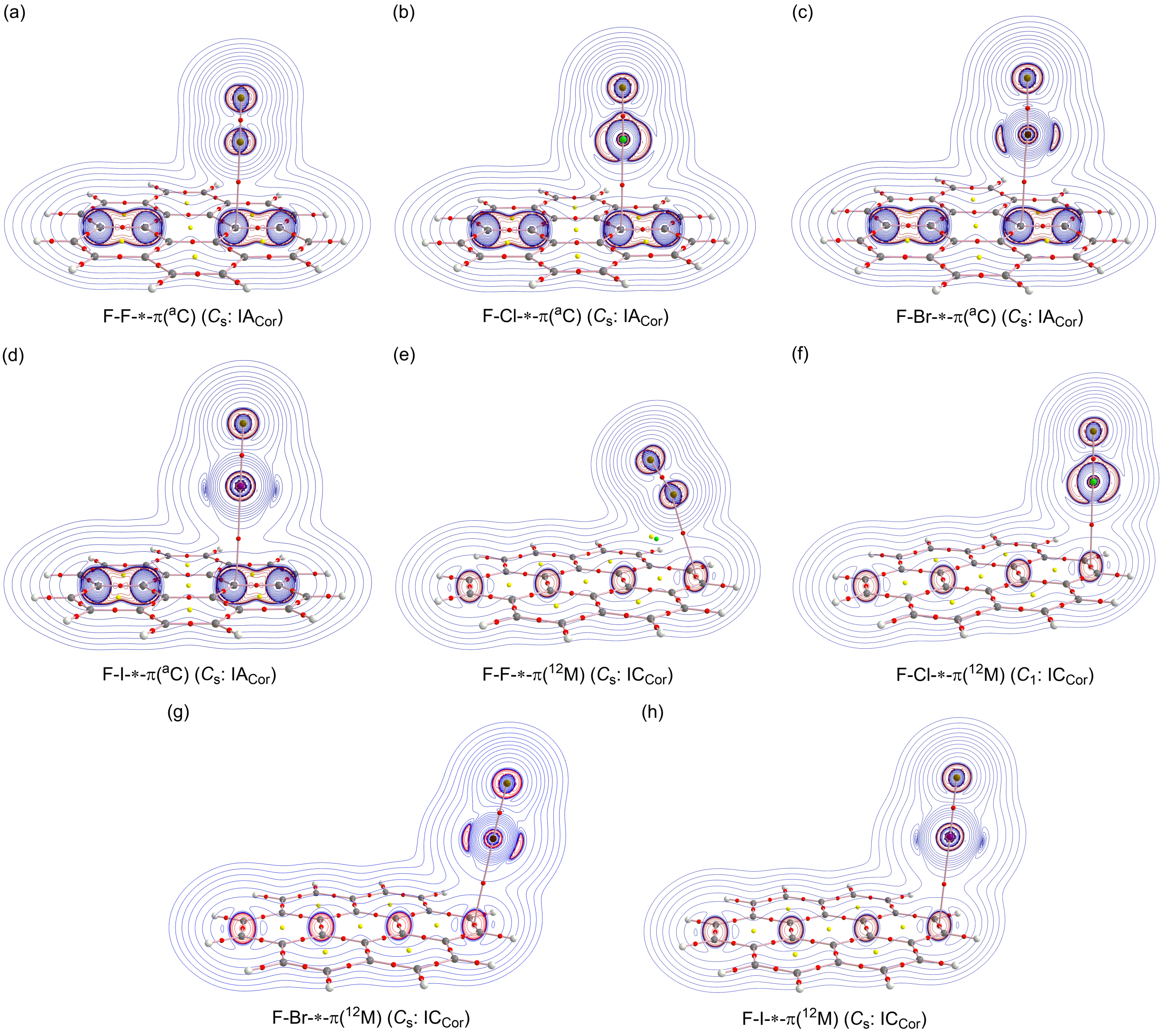

Fig. S5 Negative Laplacian for F–F-∗-π($C_{24}H_{12}$) (C_1: IA_{cor}) (a), F–Cl-∗-π($C_{24}H_{12}$) (C_s: IA_{cor}) (b), F–Br-∗-π($C_{24}H_{12}$) (C_s: IA_{cor}) (c), F–I-∗-π($C_{24}H_{12}$) (C_s: IA_{cor}) (d), F–F-∗-π($C_{24}H_{12}$) (C_1: IC_{cor}) (e), F–Cl-∗-π($C_{24}H_{12}$) (C_s: IC_{cor}) (f), F–Br-∗-π($C_{24}H_{12}$) (C_s: IC_{cor}) (g) and F–I-∗-π($C_{24}H_{12}$) (C_s: IC_{cor}) (h), drawn similarly to the case Fig. 2 in the text with M06-2X/BSS-SA. Blue and red lines correspond to the positive and negative values, respectively.

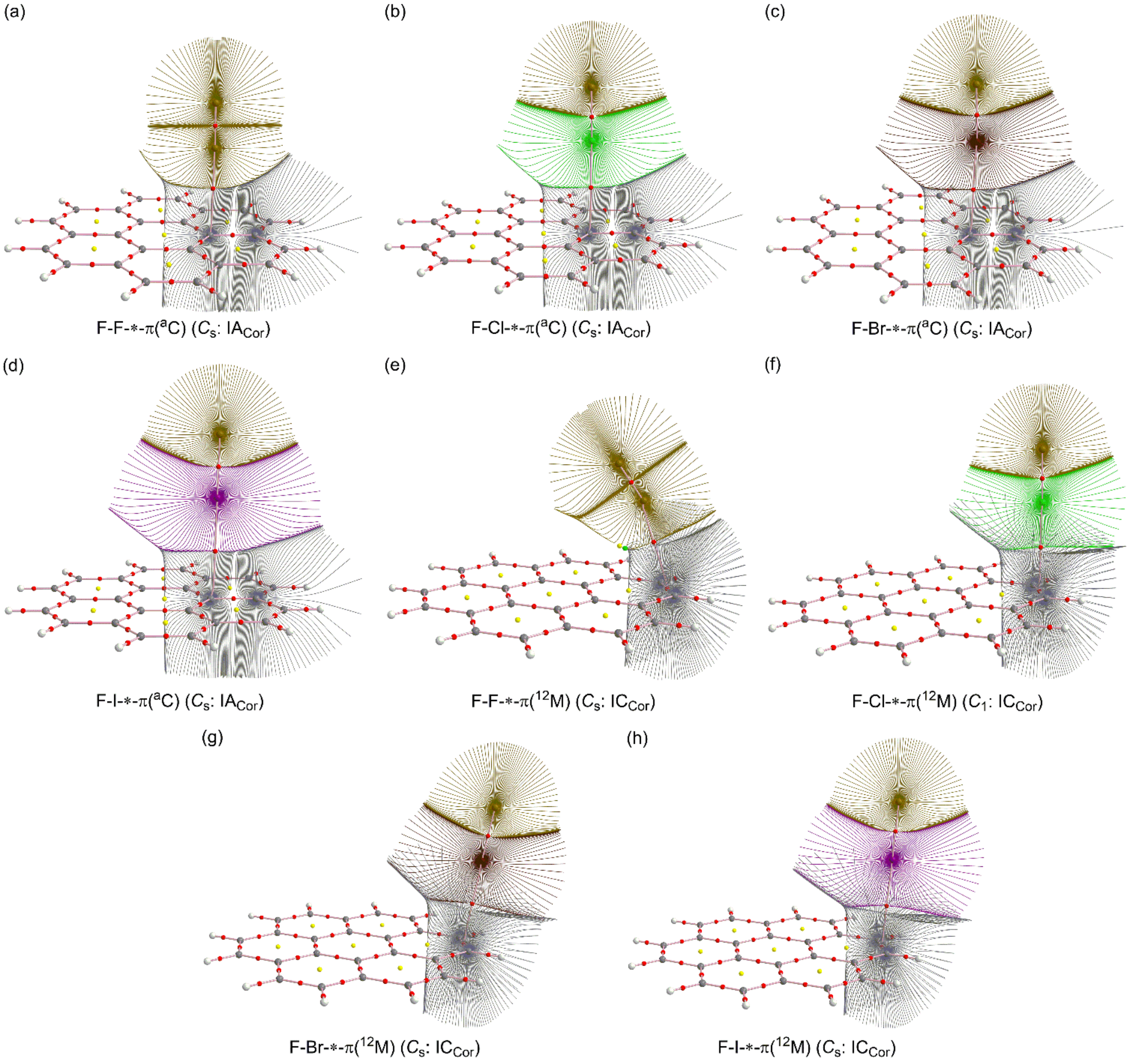

Fig. S6 Trajectory plots for F–F-∗-π($C_{24}H_{12}$) (C_1: IA_{cor}) (a), F–Cl-∗-π($C_{24}H_{12}$) (C_s: IA_{cor}) (b), F–Br-∗-π($C_{24}H_{12}$) (C_s: IA_{cor}) (c), F–I-∗-π($C_{24}H_{12}$) (C_s: IA_{cor}) (d), F–F-∗-π($C_{24}H_{12}$) (C_1: IC_{cor}) (e), F–Cl-∗-π($C_{24}H_{12}$) (C_s: IC_{cor}) (f), F–Br-∗-π($C_{24}H_{12}$) (C_s: IC_{cor}) (g) and F–I-∗-π($C_{24}H_{12}$) (C_s: IC_{cor}) (h), drawn similarly to case of Fig. 2 in the text with M06-2X/BSS-SA. Colors and marks are the same as those in Fig. 2.

Table S3. Lengths of bond paths (r_{BP}) with components (r_{BP-1} and r_{BP-2}) and the corresponding straight-line distances (R_{SL}) in X–H-∗-π($C_{24}H_{12}$) and Y–X-∗-π($C_{24}H_{12}$), evaluated at the M06-2X with BSS-SA[a,b]

Y–X-∗-π($C_{24}H_{12}$) (symmetry: type)	r_{BP-1} (Å)	r_{BP-2} (Å)	r_{BP} (Å)	R_{SL}[c] (Å)	Δr_{BP}[d] (Å)
F–H-∗-π(^{3}C) (C_1: IB_{Cor})	0.8087	1.5158	2.3245	2.2609	0.06_4
Br–H-∗-π(^{2}C) (C_1: IB'_{Cor})	0.9944	1.9704	2.9649	2.6197	0.34_5
I–H-∗-π(^{3}C) (C_1: IB_{Cor})	1.0348	1.7736	2.8084	2.6427	0.16_6
F–H-∗-π(^{12}M) (C_s: IC_{Cor})	0.7785	1.4266	2.2051	2.1815	0.02_4
Cl–H-∗-π(^{12}M) (C_1: IC_{Cor})	0.9267	1.5382	2.4649	2.4502	0.01_5
Br–H-∗-π(^{12}M) (C_s: IC_{Cor})	0.9710	1.5654	2.5363	2.5236	0.01_3
I–H-∗-π(^{12}M) (C_s: IC_{Cor})	1.0151	1.5804	2.5955	2.5847	0.01_1
F–F-∗-π(^{a}C) (C_s: IA_{Cor})	1.2741	1.5145	2.7886	2.7873	0.00_1
Cl–Cl-∗-π(^{a}C) (C_1: IA_{Cor})	1.5332	1.5064	3.0397	3.0381	0.00_2
Br–Br-∗-π(^{a}C) (C_s: IA_{Cor})	1.6161	1.5147	3.1308	3.1293	0.00_1
I–I-∗-π(^{a}C) (C_s: IA_{Cor})	1.7703	1.5424	3.3127	3.3116	0.00_1
F–Cl-∗-π(^{a}C) (C_s: IA_{Cor})	1.4650	1.4775	2.9425	2.9409	0.00_2
F–Br-∗-π(^{a}C) (C_s: IA_{Cor})	1.5291	1.4836	3.0127	3.0096	0.00_3
F–I-∗-π(^{a}C) (C_s: IA_{Cor})	1.6496	1.5118	3.1615	3.1554	0.00_6
F–F-∗-π(^{12}M) (C_s: IC_{Cor})	1.3368	1.5788	2.9156	2.9010	0.01_5
Cl–Cl-∗-π(^{12}M) (C_1: IC_{Cor})	1.5159	1.4961	3.0120	3.0107	0.00_1
Br–Br-∗-π(^{12}M) (C_1: IC_{Cor})	1.5891	1.9026	3.4916	3.0801	0.41_2
I–I-∗-π(^{12}M) (C_s: IC_{Cor})	1.7744	1.5642	3.3386	3.3370	0.00_2
F–Cl-∗-π(^{12}M) (C_1: IC_{Cor})	1.4244	2.1040	3.5284	2.8523	0.67_6
F–Br-∗-π(^{12}M) (C_s: IC_{Cor})	1.4591	1.4069	2.8660	2.8616	0.00_4
F–I-∗-π(^{12}M) (C_s: IC_{Cor})	1.5854	1.4464	3.0318	3.0277	0.00_4

[a] See text for BSS-SA. [b] See text for r_{BP-1} and r_{BP-2}, where $r_{BP} = r_{BP-1} + r_{BP-2}$. [c] $R_{SL} = r_1$. See Scheme 1 of the text for the definition of the structural parameters. [d] $\Delta r_{BP} = r_{BP} - R_{SL}$.

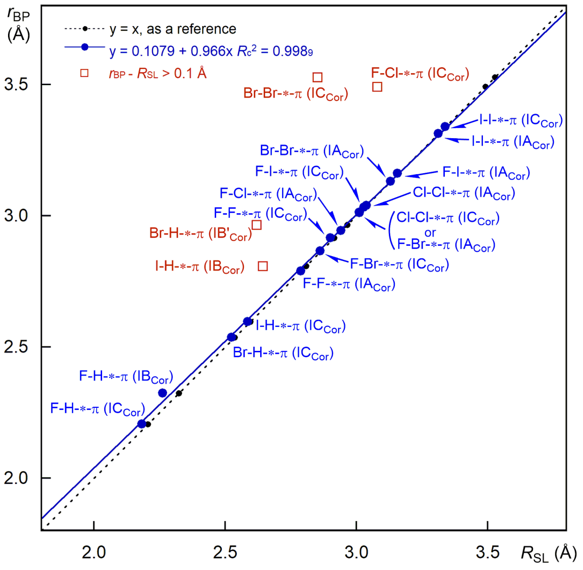

Fig. S7 Plot of r_{BP} *versus* R_{SL} for HX-∗-π($C_{24}H_{12}$) and XY-∗-π($C_{24}H_{12}$) (X, Y = F, Cl, Br and I) evaluated with M06-2X/BSS-SA.

Table S4. Structural parameters for X–H---π($C_{24}H_{12}$) and Y–X---π($C_{24}H_{12}$), optimized with M06-2X/BSS-SA[a,b]

Y–X-*-π($C_{24}H_{12}$) (symmetry: type)	r_1 (Å)	r_2 (Å)	θ_1 (°)	θ_2 (°)	ϕ_1 (°)	ϕ_2 (°)	ΔE_{ES}[c,d] (kJ mol^{-1})	ΔE_{Ent}[c,e] (kJ mol^{-1})
F–H---π(M_o) (C_{2v}: ID_{Cor})[f]	2.2195	0.9222	90.00	180.00	-90.00	180.00	-14.3	-15.5
Cl–H---π(M_o) (C_{2v}: ID_{Cor})[g]	2.3065	1.2814	90.00	180.00	-90.00	180.00	-13.8	-11.1
Br–H---π(M_o) (C_{2v}: ID_{Cor})[f]	2.3363	1.4232	90.00	180.00	-90.00	180.00	-13.7	-14.5
I–H---π(M_o) (C_{2v}: ID_{Cor})	2.3655	1.6177	90.00	180.00	-90.00	180.00	-13.8	-7.6
F–F---π(M_o) (C_{2v}: ID_{Cor})[h]	2.8942	1.3655	90.00	180.00	-90.00	180.00	-6.3	-10.0
Cl–Cl---π(M_o) (C_{2v}: ID_{Cor})[f]	3.0869	1.9916	90.01	180.00	-89.98	180.00	-12.0	-12.9
Br–Br---π(M_o) (C_{2v}: ID_{Cor})[f]	3.1855	2.2882	90.01	180.00	-90.01	180.00	-15.2	-16.3
I–I---π(M_o) (C_{2v}: ID_{Cor})[f]	3.3489	2.6747	90.02	180.00	-90.01	180.00	-19.4	-20.7
F–Cl---π(M_o) (C_{2v}: ID_{Cor})[f]	3.0492	1.6224	90.01	180.00	-89.98	180.00	-14.5	-15.5
F–Br---π(M_o) (C_{2v}: ID_{Cor})[f]	3.1417	1.7577	90.02	180.00	-90.01	180.00	-19.5	-20.6
F–I---π(M_o) (C_{2v}: ID_{Cor})[i]	3.2795	1.9147	90.02	180.00	-90.01	180.00	-25.5	-31.8

[a] See text for BSS-SA. [b] See Scheme 2 of the text for the definition of the structural parameters. [c] $\Delta E = E$(X–H---π($C_{24}H_{12}$)/Y–X---π($C_{24}H_{12}$)) – (E(X–H/Y–X) + E($C_{24}H_{12}$)). [d] ΔE_{ES} stands for ΔE on the energy surface. [e] ΔE_{Ent} stands for ΔE with the correction of the heat of enthalpy. [f] Two imaginary frequencies being predicted for each. [g] One imaginary frequency being predicted for each. [h] Three imaginary frequencies being predicted for each. [i] Four imaginary frequencies being predicted for each.

Table S5. QTAIM functions and QTAIM-DFA parameters for X–H-*-π($C_{24}H_{12}$) and Y–X-*-π($C_{24}H_{12}$) (X, Y = F, Cl, Br and I), evaluated with M06-2X/BSS-SA[a,b]

Y–X-*-π($C_{24}H_{12}$) (symmetry: type)	$\rho_b(\boldsymbol{r}_c)$ (ea_o^{-3})	$c\nabla^2\rho_b(\boldsymbol{r}_c)$[c] (au)	$H_b(\boldsymbol{r}_c)$ (au)	$k_b(\boldsymbol{r}_c)$[d]	R (au)	θ (°)	freq (cm^{-1})	k_f (mDyne Å^{-1})	θ_p (°)	κ_p (au^{-1})
F–H-*-π(M_o) (C_{2v}: ID_{Cor})	0.0075	0.0035	0.0012	-0.783	0.0037	70.4	138.5	0.063	79.5	68.3
Cl–H-*-π(M_o) (C_{2v}: ID_{Cor})	0.0074	0.0032	0.0012	-0.773	0.0034	69.7	134.2	0.056	75.5	83.2
Br–H-*-π(M_o) (C_{2v}: ID_{Cor})	0.0074	0.0031	0.0012	-0.770	0.0033	69.5	134.1	0.056	76.1	64.0
I–H-*-π(M_o) (C_{2v}: ID_{Cor})	0.0074	0.0031	0.0012	-0.772	0.0033	69.6	133.0	0.054	76.5	89.2
F–F-*-π(M_o) (C_{2v}: ID_{Cor})	0.0045	0.0025	0.0011	-0.713	0.0027	65.9	127.8	0.048	66.6	29.6
Cl–Cl-*-π(M_o) (C_{2v}: ID_{Cor})	0.0064	0.0030	0.0013	-0.725	0.0032	66.7	149.3	0.082	74.4	75.9
Br–Br-*-π(M_o) (C_{2v}: ID_{Cor})	0.0067	0.0028	0.0011	-0.763	0.0030	69.0	147.6	0.075	76.0	89.4
I–I-*-π(M_o) (C_{2v}: ID_{Cor})	0.0068	0.0026	0.0010	-0.764	0.0028	69.1	145.5	0.071	75.1	142.8
F–Cl-*-π(M_o) (C_{2v}: ID_{Cor})	0.0066	0.0031	0.0013	-0.737	0.0033	67.4	159.3	0.106	74.5	78.5
F–Br-*-π(M_o) (C_{2v}: ID_{Cor})	0.0069	0.0029	0.0011	-0.776	0.0031	69.9	154.2	0.089	76.1	101.8
F–I-*-π(M_o) (C_{2v}: ID_{Cor})	0.0072	0.0027	0.0010	-0.786	0.0029	70.6	151.4	0.081	77.3	158.8

[a] See text for BSS-SA. [b] Data are given at BCP, which is shown by X-*-π. [c] $c\nabla^2\rho_b(\boldsymbol{r}_c) = H_b(\boldsymbol{r}_c) - V_b(\boldsymbol{r}_c)/2$, where $c = \hbar^2/8m$. [d] $k_b(\boldsymbol{r}_c) = V_b(\boldsymbol{r}_c)/G_b(\boldsymbol{r}_c)$.

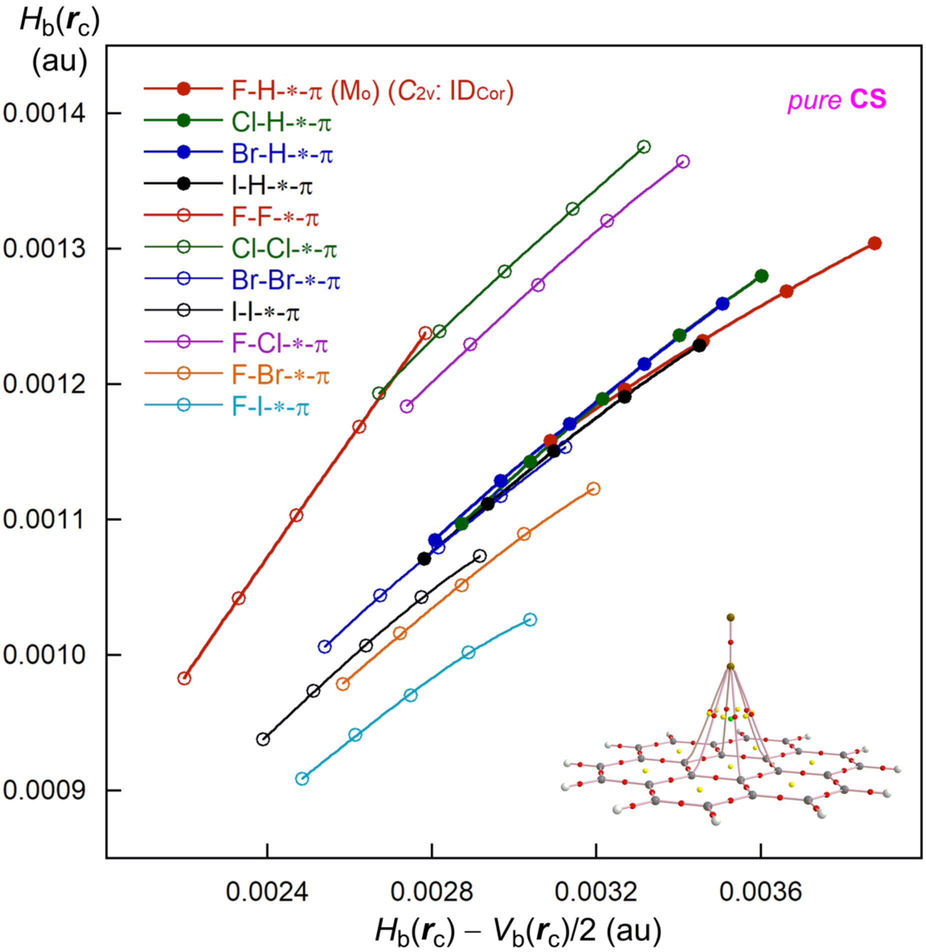

Fig. S8 QTAIM-DFA plots ($H_b(\boldsymbol{r}_c)$ versus $H_b(\boldsymbol{r}_c) - V_b(\boldsymbol{r}_c)/2$) for H–X-∗-π($C_{24}H_{12}$) ($C_{2v}$: ID_{Cor}) and Y–X-∗-π($C_{24}H_{12}$) (C_{2v}: ID_{Cor}) (X, Y = F, Cl, Br and I), evaluated with M06-2X/BSS-SA.

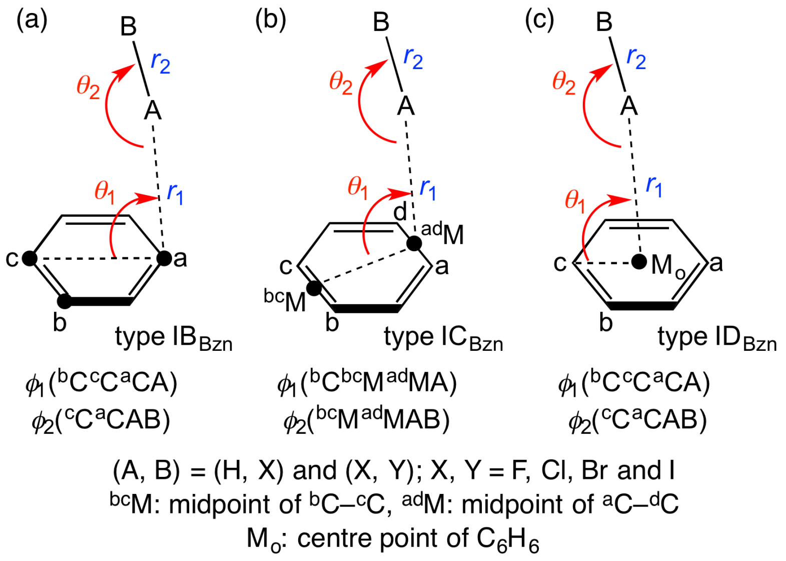

Scheme S3. Structures of X–H-∗-π(C_6H_6) and Y–X-∗-π(C_6H_6) to be clarified with the definition of structural parameters and types, where X–H = F–H, Cl–H, Br–H and I–H and Y–X = F–F, Cl–Cl, Br–Br, I–I, F–Cl, F–Br and F–I ((A, B) = (H, X) and (X, Y); X, Y = F, Cl, Br and I)).

Table S6. Structural parameters for X–H---π(C_6H_6) and Y–X---π(C_6H_6), optimized with M06-2X/BSS-SA[a,b]

Y–X-*-π(C_6H_6) (symmetry: type)	r_1 (Å)	r_2 (Å)	θ_1 (°)	θ_2 (°)	ϕ_1 (°)	ϕ_2 (°)	ΔE_{ES}[c,d] (kJ mol^{-1})	ΔE_{Ent}[c,e] (kJ mol^{-1})
F–H-*-π(C_6H_6) (C_s: IB_{Bzn})	2.3970	0.9248	64.09	162.47	-90.00	180.00	-22.2	-17.8
Cl–H-*-π(C_6H_6) (C_s: IB_{Bzn})	2.6165	1.2843	61.44	160.60	-89.99	180.00	-18.1	-13.8
Br–H-*-π(C_6H_6) (C_s: IB_{Bzn})	2.6129	1.4245	63.64	168.91	-90.00	180.00	-17.6	-11.6
I–H-*-π(C_6H_6) (C_s: IB_{Bzn})	2.6896	1.6217	62.49	162.23	-90.00	180.00	-15.6	-10.1
F–F-*-π(C_6H_6) (C_s: IB_{Bzn})	2.9904	1.3675	72.99	171.79	-90.06	0.00	-6.4	-2.5
Cl–Cl-*-π(C_6H_6) (C_s: IB_{Bzn})[f]	3.1165	1.9955	82.45	176.87	-89.99	180.00	-12.5	-11.1
Br–Br-*-π(C_6H_6) (C_s: IB_{Bzn})	3.1442	2.2956	86.72	175.62	-89.95	180.00	-16.1	-12.1
I–I-*-π(C_6H_6) (C_s: IB_{Bzn})[f]	3.4027	2.6783	82.44	176.65	-89.98	180.00	-18.0	-16.6
F–Cl-*-π(C_6H_6) (C_s: IB_{Bzn})	2.9362	1.6292	87.54	176.52	-89.96	180.00	-19.0	-15.1
F–Br-*-π(C_6H_6) (C_s: IB_{Bzn})[f]	2.9764	1.7657	89.33	175.79	-89.90	180.00	-25.6	-24.2
F–I-*-π(C_6H_6) (C_s: IB_{Bzn})[f]	3.1162	1.9233	91.67	174.26	-89.81	180.00	-31.1	-29.5
F–H-*-π(C_6H_6) (C_s: IC_{Bzn})	2.3227	0.9248	68.22	166.66	-90.00	180.00	-22.2	-17.9
Cl–H-*-π(C_6H_6) (C_s: IC_{Bzn})	2.5382	1.2843	64.90	163.97	-90.00	180.00	-18.1	-13.8
Br–H-*-π(C_6H_6) (C_s: IC_{Bzn})[f]	2.5801	1.4257	64.33	159.58	-90.00	180.00	-17.4	-15.1
I–H-*-π(C_6H_6) (C_s: IC_{Bzn})	non	non	non	non	non	non	non	non
F–F-*-π(C_6H_6) (C_s: IC_{Bzn})	2.9271	1.3676	76.98	167.93	-90.00	0.00	-6.4	-2.5
Cl–Cl-*-π(C_6H_6) (C_s: IC_{Bzn})[f]	3.1196	1.9951	85.34	179.72	-90.00	0.00	-12.4	-11.1
Br–Br-*-π(C_6H_6) (C_s: IC_{Bzn})[f]	3.1457	2.2954	89.62	179.03	-90.00	180.00	-15.9	-14.4
I–I-*-π(C_6H_6) (C_s: IC_{Bzn})[f]	3.3905	2.6781	85.20	179.54	-90.00	180.00	-18.0	-16.6
F–Cl-*-π(C_6H_6) (C_s: IC_{Bzn})	2.9145	1.6296	91.70	179.75	-90.00	0.00	-19.1	-15.2
F–Br-*-π(C_6H_6) (C_s: IC_{Bzn})	2.9459	1.7648	96.05	178.03	-90.00	180.00	-25.9	-21.8
F–I-*-π(C_6H_6) (C_s: IC_{Bzn})	3.0957	1.9239	95.99	177.98	-90.00	180.00	-31.7	-27.3

[a] See text for BSS-SA. [b] See scheme S3 of the text for the definition of the structural parameters. [c] $\Delta E = E$(X–H---π(C_6H_6)/Y–X---π(C_6H_6)) – (E(X–H/Y–X) + E(C_6H_6)). [d] ΔE_{ES} stands for ΔE on the energy surface. [e] ΔE_{Ent} stands for ΔE with the correction of the heat of enthalpy. [f] one imaginary frequencies being predicted for each.

Table S7. QTAIM functions and QTAIM-DFA parameters for X–H-∗-π(C_6H_6) and Y–X-∗-π(C_6H_6) (X, Y = F, Cl, Br and I), evaluated with M06-2X/BSS-SA[a,b]

X–H-∗-π(C_6H_6)<br>Y–X-∗-π(C_6H_6)<br>(symmetry: type)	$\rho_b(\boldsymbol{r}_c)$<br>(ea_o^{-3})	$c\nabla^2\rho_b(\boldsymbol{r}_c)$[c]<br>(au)	$H_b(\boldsymbol{r}_c)$<br>(au)	$k_b(\boldsymbol{r}_c)$[d]	R<br>(au)	θ<br>(°)	freq<br>(cm^{-1})	k_f<br>(mDyne Å^{-1})	θ_p<br>(°)	κ_p<br>(au^{-1})
F–H-∗-π(C_6H_6) (C_s: IB_{Bzn})	0.0120	0.0049	0.0010	-0.881	0.0050	78.0	122.9	0.0703	103.0	230.6
Cl–H-∗-π(C_6H_6) (C_s: IB_{Bzn})	0.0089	0.0038	0.0012	-0.805	0.0040	71.9	92.5	0.0564	82.6	143.2
Br–H-∗-π(C_6H_6) (C_s: IB_{Bzn})	0.0092	0.0037	0.0012	-0.810	0.0039	72.3	72.3	0.0332	83.9	146.1
I–H-∗-π(C_6H_6) (C_s: IB_{Bzn})	0.0085	0.0034	0.0012	-0.794	0.0036	71.1	61.6	0.0163	80.9	131.0
F–F-∗-π(C_6H_6) (C_s: IB_{Bzn})	0.0071	0.0040	0.0018	-0.710	0.0044	65.8	73.1	0.0213	67.3	27.8
Cl–Cl-∗-π(C_6H_6) (C_s: IB_{Bzn})[e]	0.0101	0.0044	0.0015	-0.795	0.0047	71.2	78.3	0.0341	84.5	104.9
Br–Br-∗-π(C_6H_6) (C_s: IB_{Bzn})	0.0115	0.0045	0.0013	-0.840	0.0047	74.6	101.8	0.0325	88.6	129.7
I–I-∗-π(C_6H_6) (C_s: IB_{Bzn})[e]	0.0096	0.0035	0.0011	-0.815	0.0037	72.7	72.8	0.0202	86.2	132.5
F–Cl-∗-π(C_6H_6) (C_s: IB_{Bzn})	0.0135	0.0058	0.0014	-0.857	0.0060	75.9	116.0	0.0609	93.9	119.0
F–Br-∗-π(C_6H_6) (C_s: IB_{Bzn})[e]	0.0147	0.0058	0.0011	-0.891	0.0059	78.9	101.8	0.0457	100.2	155.7
F–I-∗-π(C_6H_6) (C_s: IB_{Bzn})[e]	0.0144	0.0051	0.0007	-0.924	0.0051	81.9	103.9	0.0346	112.6	279.0
F–H-∗-π(C_6H_6) (C_s: IC_{Bzn})	0.0119	0.0049	0.0011	-0.877	0.0050	77.6	122.6	0.0690	102.0	228.5
Cl–H-∗-π(C_6H_6) (C_s: IC_{Bzn})	0.0088	0.0038	0.0012	-0.802	0.0040	71.7	92.8	0.0565	82.0	143.5
Br–H-∗-π(C_6H_6) (C_s: IC_{Bzn})[e]	0.0085	0.0036	0.0012	-0.794	0.0038	71.1	77.0	0.0282	79.4	149.6
I–H-∗-π(C_6H_6) (C_s: IC_{Bzn})	non	non	non	non	non	non	non	non	non	non
F–F-∗-π(C_6H_6) (C_s: IC_{Bzn})	0.0073	0.0041	0.0019	-0.708	0.0045	65.7	68.5	0.0188	66.8	21.2
Cl–Cl-∗-π(C_6H_6) (C_s: IC_{Bzn})[e]	0.0096	0.0043	0.0016	-0.772	0.0046	69.7	79.1	0.0305	80.8	91.0
Br–Br-∗-π(C_6H_6) (C_s: IC_{Bzn})[e]	0.0111	0.0045	0.0014	-0.815	0.0047	72.6	74.0	0.0170	83.3	83.4
I–I-∗-π(C_6H_6) (C_s: IC_{Bzn})[e]	0.0093	0.0035	0.0012	-0.797	0.0037	71.3	70.8	0.0153	82.6	132.3
F–Cl-∗-π(C_6H_6) (C_s: IC_{Bzn})	0.0136	0.0060	0.0017	-0.835	0.0062	74.2	132.4	0.0690	89.1	108.3
F–Br-∗-π(C_6H_6) (C_s: IC_{Bzn})	0.0151	0.0061	0.0014	-0.869	0.0063	77.0	115.6	0.0803	95.2	168.9
F–I-∗-π(C_6H_6) (C_s: IC_{Bzn})	0.0146	0.0053	0.0010	-0.896	0.0054	79.4	126.8	0.0559	105.1	281.5

[a] See text for BSS-SA. [b] Data are given at BCP, which is shown by H-∗-π and X-∗-π. [c] $c\nabla^2\rho_b(\boldsymbol{r}_c) = H_b(\boldsymbol{r}_c) - V_b(\boldsymbol{r}_c)/2$, where $c = \hbar^2/8m$. [d] $k_b(\boldsymbol{r}_c) = V_b(\boldsymbol{r}_c)/G_b(\boldsymbol{r}_c)$. [e] One imaginary frequency being predicted for each.

Table S8. Structural parameters for X–H---π(C_6H_6) (C_{2v}: ID_{Bzn}) and Y–X---π(C_6H_6) (C_{2v}: ID_{Bzn}), optimized with M06-2X/BSS-SA[a,b]

Y–X-*-π(C_6H_6) (symmetry)	r_1 (Å)	r_2 (Å)	θ_1 (°)	θ_2 (°)	ϕ_1 (°)	ϕ_2 (°)	ΔE_{ES}[c,d] (kJ mol^{-1})	ΔE_{Ent}[c,e] (kJ mol^{-1})
F–H---π(C_6H_6) (C_{2v})	2.1554	0.9243	89.99	180.00	-90.01	180.00	-22.2	-17.9
Cl–H---π(C_6H_6) (C_{2v})	2.2942	1.2840	90.00	180.00	-90.00	180.00	-18.2	-12.6
Br–H---π(C_6H_6) (C_{2v})[g]	2.3192	1.4261	90.00	180.00	-90.00	180.00	-17.2	-18.1
I–H---π(C_6H_6) (C_{2v})	2.3827	1.6201	90.00	180.00	-90.02	180.00	-15.5	-9.2
F–F---π(C_6H_6) (C_{2v})[g]	2.9803	1.3662	89.99	180.00	-90.01	180.00	-5.5	-6.6
Cl–Cl---π(C_6H_6) (C_{2v})[g]	3.1597	1.9925	89.99	180.00	-90.02	180.00	-10.3	-11.4
Br–Br---π(C_6H_6) (C_{2v})[g]	3.2495	2.2893	89.98	180.00	-90.03	180.00	-13.2	-14.3
I–I---π(C_6H_6) (C_{2v})[g]	3.4528	2.6755	89.99	180.00	-90.01	180.00	-15.9	-17.1
F–Cl---π(C_6H_6) (C_{2v})[g]	3.0975	1.6237	89.99	180.00	-90.02	180.00	-13.3	-14.3
F–Br---π(C_6H_6) (C_{2v})[g]	3.2011	1.7586	89.98	180.00	-90.04	180.00	-17.9	-18.9
F–I---π(C_6H_6) (C_{2v})[h]	3.3538	1.9150	89.97	180.00	-90.06	180.00	-22.6	-28.6

[a] See text for BSS-SA. [b] See Scheme S3 of the text for the definition of the structural parameters. [c] $\Delta E = E$(X–H---π($C_{24}H_{12}$)/Y–X---π($C_{24}H_{12}$)) – (E(X–H/Y–X) + E($C_{24}H_{12}$)). [d] ΔE_{ES} stands for ΔE on the energy surface. [e] ΔE_{Ent} stands for ΔE with the correction of the heat of enthalpy. [f] Two imaginary frequencies being predicted for each. [g] Two imaginary frequencies being predicted for each. [h] Four imaginary frequencies being predicted for each.

Table S9. QTAIM functions and QTAIM-DFA parameters for X–H-*-π(C_6H_6) (C_{2v}: ID_{Bzn}) and Y–X-*-π(C_6H_6) (C_{2v}: ID_{Bzn}) (X, Y = F, Cl, Br and I), evaluated with M06-2X/BSS-SA[a,b]

Y–X-*-π($C_{24}H_{12}$) (symmetry)	$\rho_b(\boldsymbol{r}_c)$ (ea_o^{-3})	$c\nabla^2\rho_b(\boldsymbol{r}_c)$[c] (au)	$H_b(\boldsymbol{r}_c)$ (au)	$k_b(\boldsymbol{r}_c)$[d]	R (au)	θ (°)	freq (cm^{-1})	k_f (mDyne Å^{-1})	θ_p (°)	κ_p (au^{-1})
F–H-*-π(C_6H_6) (C_{2v})	0.0090	0.0043	0.0013	-0.813	0.0045	72.5	134.1	0.093	83.3	106.6
Cl–H-*-π(C_6H_6) (C_{2v})	0.0081	0.0036	0.0013	-0.786	0.0038	70.6	108.3	0.078	78.3	118.1
Br–H-*-π(C_6H_6) (C_{2v})	0.0081	0.0035	0.0012	-0.784	0.0037	70.4	71.8	0.033	76.9	111.5
I–H-*-π(C_6H_6) (C_{2v})	0.0077	0.0032	0.0012	-0.779	0.0035	70.1	63.9	0.023	77.0	79.5
F–F-*-π(C_6H_6) (C_{2v})	0.0042	0.0023	0.0010	-0.719	0.0025	66.3	94.1	0.060	65.9	4.9
Cl–Cl-*-π(C_6H_6) (C_{2v})	0.0061	0.0028	0.0012	-0.718	0.0030	66.2	44.4	0.013	73.0	72.8
Br–Br-*-π(C_6H_6) (C_{2v})	0.0064	0.0027	0.0010	-0.760	0.0029	68.8	62.6	0.021	75.0	67.9
I–I-*-π(C_6H_6) (C_{2v})	0.0061	0.0023	0.0009	-0.757	0.0025	68.6	45.0	0.010	73.9	86.7
F–Cl-*-π(C_6H_6) (C_{2v})	0.0065	0.0030	0.0013	-0.736	0.0032	67.3	91.0	0.056	74.4	66.7
F–Br-*-π(C_6H_6) (C_{2v})	0.0066	0.0027	0.0010	-0.773	0.0029	69.7	83.6	0.042	75.8	69.8
F–I-*-π(C_6H_6) (C_{2v})	0.0067	0.0025	0.0009	-0.780	0.0027	70.2	71.6	0.028	76.4	98.6

[a] See text for BSS-SA. [b] Data are given at BCP, which is shown by H-*-π and X-*-π. [c] $c\nabla^2\rho_b(\boldsymbol{r}_c) = H_b(\boldsymbol{r}_c) - V_b(\boldsymbol{r}_c)/2$, where $c = \hbar^2/8m$. [d] $k_b(\boldsymbol{r}_c) = V_b(\boldsymbol{r}_c)/G_b(\boldsymbol{r}_c)$.

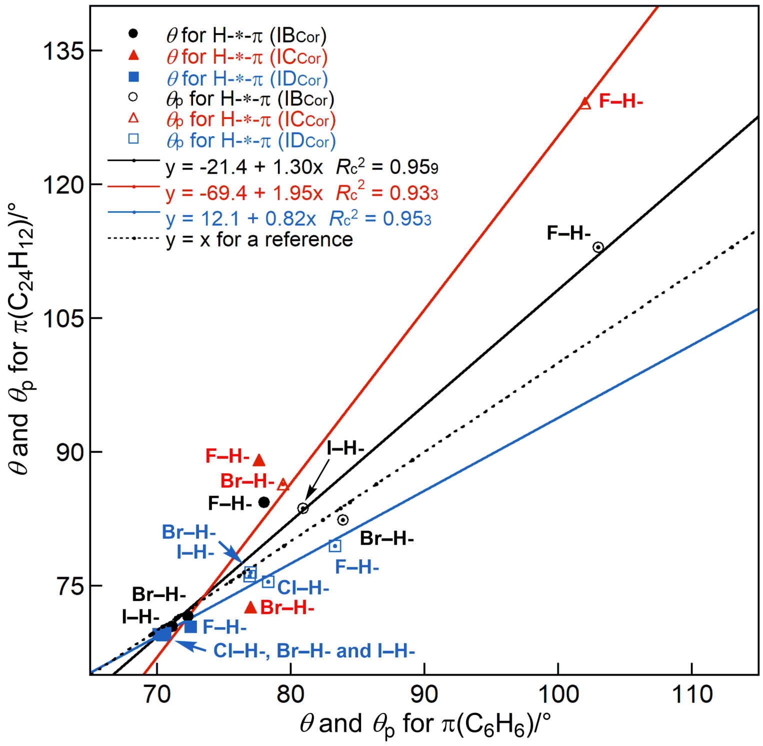

Fig. S9 Plots of θ and θ_p for X–H-∗-π($C_{24}H_{12}$) (X = F, Cl, Br and I) versus those for X–H-∗-π(C_6H_6), evaluated with M06-2X/BSS-SA.

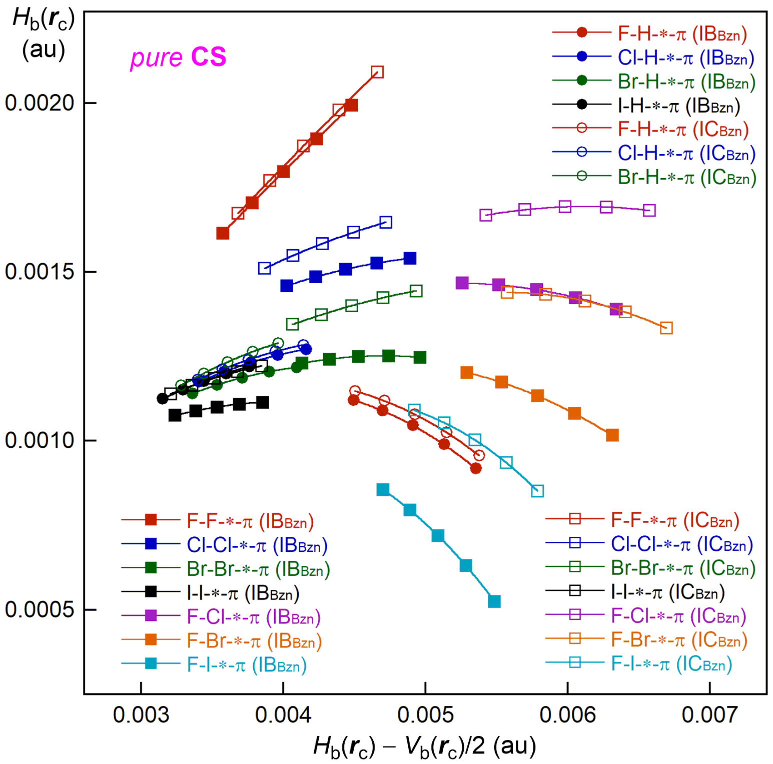

Fig. S10 QTAIM-DFA plots ($H_b(\boldsymbol{r}_c)$ versus $H_b(\boldsymbol{r}_c) - V_b(\boldsymbol{r}_c)/2$) for H–X-∗-π($C_6H_6$) and Y–X-∗-π($C_6H_6$) (X, Y = F, Cl, Br and I). Marks and colours are shown in the figure, where solid marks for interaction between C atom and H or X atoms and hollow marks between BCP for midpoint of C–C and H or X atoms correspond to the data evaluated with M06-2X/BSS-SA.

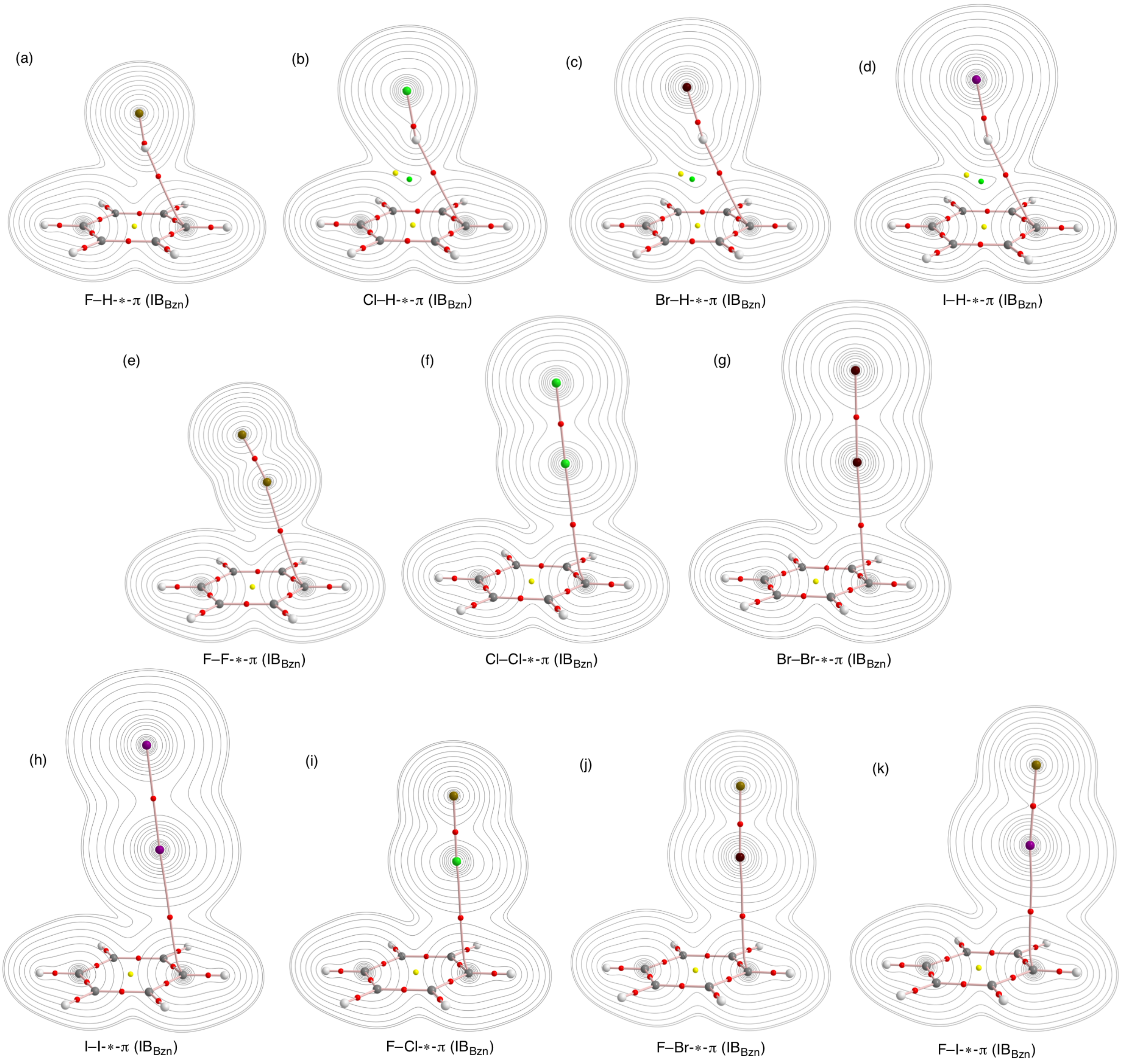

Fig. S11 Molecular graphs for F–H-∗-π(C_6H_6) (C_s: type IB_{Bzn}) (a), Cl–H-∗-π(C_6H_6) (C_s: type IB_{Bzn}) (b), Br–H-∗-π(C_6H_6) (C_s: type IB_{Bzn}) (c), I–H-∗-π(C_6H_6) (C_s: type IB_{Bzn}) (d), F–F-∗-π(C_6H_6) (C_s: type IB_{Bzn}) (e), Cl–Cl-∗-π(C_6H_6) (C_s: type IB_{Bzn}) (f), Br–Br-∗-π(C_6H_6) (C_s: type IB_{Bzn}) (g), I–I-∗-π(C_6H_6) (C_s: type IB_{Bzn}) (h), F–Cl-∗-π(C_6H_6) (C_s: type IB_{Bzn}) (i), F–Br-∗-π(C_6H_6) (C_s: type IB_{Bzn}) (j) and F–I-∗-π(C_6H_6) (C_s: type IB_{Bzn}) (k), calculated with M06-2X/BSS-SA. BCPs are denoted by red dots, RCPs by yellow dots, CCPs by green dots and BPs by pink lines. Carbon atoms are in black and hydrogen atoms are in grey, with fluorine, chlorine, bromine and iodine atoms in dark yellow, green, dark purple and purple, respectively. The contour plot of $\rho(\boldsymbol{r})$ is also drawn for each on the plane containing the H-∗-^{a}C(C_6H_6) moiety for type IB_{Bzn} with the H-∗-^{2}C($C_{24}H_{12}$) moiety for type IB'_{Cor} or on the plane of H-∗-^{12}M($C_{24}H_{12}$) moiety for type IC_{Cor}, although the contour plot is drawn only partially.

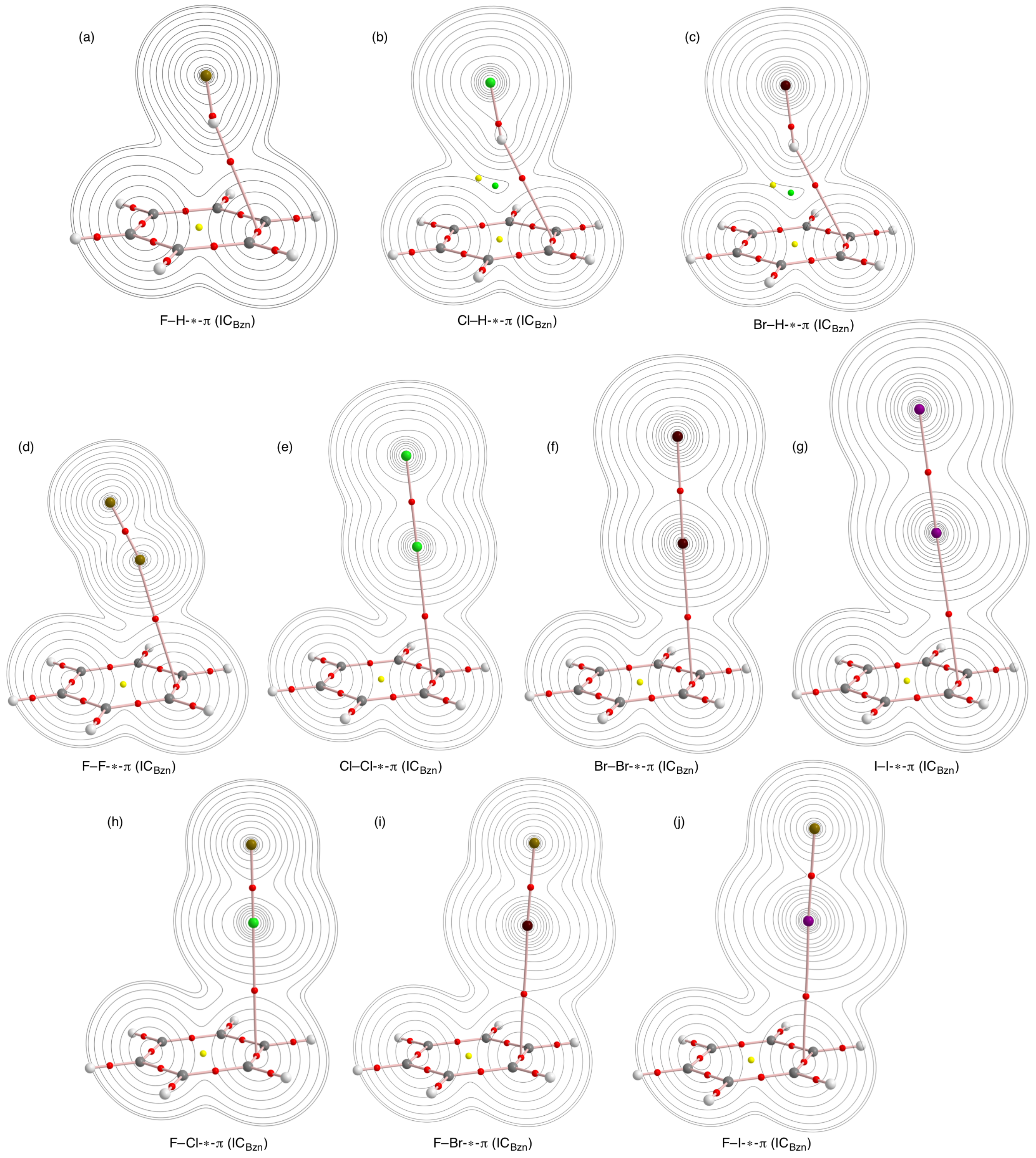

Fig. S12 Molecular graphs for F–H-∗-π(C_6H_6) (C_s: type IB$_{Bzn}$) (a), Cl–H-∗-π(C_6H_6) (C_s: type IB$_{Bzn}$) (b), Br–H-∗-π(C_6H_6) (C_s: type IB$_{Bzn}$) (c), I–H-∗-π(C_6H_6) (C_s: type IB$_{Bzn}$) (d), F–F-∗-π(C_6H_6) (C_s: type IB$_{Bzn}$) (e), Cl–Cl-∗-π(C_6H_6) (C_s: type IB$_{Bzn}$) (f), Br–Br-∗-π(C_6H_6) (C_s: type IB$_{Bzn}$) (g), I–I-∗-π(C_6H_6) (C_s: type IB$_{Bzn}$) (h), F–Cl-∗-π(C_6H_6) (C_s: type IB$_{Bzn}$) (i), F–Br-∗-π(C_6H_6) (C_s: type IB$_{Bzn}$) (j) and F–I-∗-π(C_6H_6) (C_s: type IB$_{Bzn}$) (k), drawn similarly to case of Fig. S10 in the text with M06-2X/BSS-SA. Colors and marks are the same as those in Fig. S10. The contour plot of $\rho(\boldsymbol{r})$ is also drawn for each on on the plane of H-∗-^{ad}M($C_{24}H_{12}$) moiety for type IC$_{Bzn}$, although the contour plot is drawn only partially.

Table S10. Structural parameters for X–H---π($C_{24}H_{12}$) and Y–X---π($C_{24}H_{12}$), optimized with MP2/6-311G(d,p) for XH---π($C_{24}H_{12}$) and MP2/6-311G(d) for YX-∗-π($C_{24}H_{12}$)[a]

Y–X---π($C_{24}H_{12}$) (symmetry: type)	r_1 (Å)	r_2 (Å)	θ_1 (°)	θ_2 (°)	ϕ_1 (°)	ϕ_2 (°)	ΔE_{ES}[b,c] (kJ mol^{-1})	ΔE_{Ent}[b,d] (kJ mol^{-1})
F–H---π(^{a}C) (C_1: IA_{Cor})	3.7660	0.9184	127.40	168.51	-134.17	113.45	-21.9	-17.3
Cl–H---π(^{a}C) (C_s: IA_{Cor})	2.2496	1.2812	89.71	179.34	-88.88	180.00	-20.9	-16.9
Br–H---π(^{a}C) (C_s:IA_{Cor})	2.2638	1.4199	79.05	177.77	-88.92	180.00	-23.3	-19.5
I–H---π(^{a}C) (C_s:IA_{Cor})	2.3471	1.6122	72.11	174.86	-88.91	0.00	-26.0	-22.2
F–F---π(^{a}C) (C_s:IA_{Cor})	2.6632	1.4250	89.23	179.35	-88.89	0.00	-10.6	-7.0
Cl–Cl---π(^{a}C) (C_s:IA_{Cor})	2.8320	2.0481	87.94	179.63	-89.02	0.00	-23.6	-19.8
Br–Br---π(^{a}C) (C_s:IA_{Cor})	2.9180	2.3290	88.14	179.95	-89.04	0.00	-30.0	-26.1
I–I---π(^{a}C) (C_s:IA_{Cor})	3.0891	2.7355	87.38	179.55	-89.04	180.00	-36.4	-32.5
F–Cl---π(^{a}C) (C_s:IA_{Cor})	2.7446	1.6943	89.78	179.84	-89.13	180.00	-27.7	-23.9
F–Br---π(^{a}C) (C_s:IA_{Cor})	2.8064	1.8237	89.83	179.77	-89.17	180.00	-36.1	-32.3
F–I---π(^{a}C) (C_s:IA_{Cor})	2.9644	1.9865	89.28	179.40	-89.17	-180.00	-43.4	-39.4

[a] See Scheme 1 for the definition of the structural parameters. [b] ΔE = E(X–H---π($C_{24}H_{12}$)/Y–X---π($C_{24}H_{12}$)) – (E(Y–X) + E($C_{24}H_{12}$)). [c] ΔE_{ES} stands for ΔE on the energy surface. [d] ΔE_{Ent} stands for ΔE with the correction of the heat of enthalpy.

Table S11. QTAIM functions and QTAIM-DFA parameters for X–H-∗-π($C_{24}H_{12}$) and Y–X-∗-π($C_{24}H_{12}$) (X, Y = F, Cl, Br and I), evaluated with MP2/6-311G(d,p) for XH---π($C_{24}H_{12}$) and MP2/6-311G(d) for YX-∗-π($C_{24}H_{12}$)[a]

Y–X-∗-π($C_{24}H_{12}$) (symmetry: type)	$\rho_b(\boldsymbol{r}_c)$ (ea_o^{-3})	$c\nabla^2\rho_b(\boldsymbol{r}_c)$[b] (au)	$H_b(\boldsymbol{r}_c)$ (au)	$k_b(\boldsymbol{r}_c)$[c]	R (au)	θ (°)	freq. (cm^{-1})	k_e (unit[d])	θ_p (°)	κ_p (au^{-1})
F–H---π(^{a}C) (C_1: IA_{Cor})	0.0149	0.0061	0.0021	-0.787	0.0064	70.7	117.8	0.0527	84.5	148.2
Cl–H---π(^{a}C) (C_s: IA_{Cor})	0.0158	0.0055	0.0012	-0.882	0.0056	78.0	73.5	0.0334	93.1	226.1
Br–H---π(^{a}C) (C_s:IA_{Cor})	0.0161	0.0055	0.0012	-0.883	0.0056	78.1	53.5	0.0247	92.2	243.2
I–H---π(^{a}C) (C_s:IA_{Cor})	0.0147	0.0050	0.0011	-0.878	0.0051	77.7	41.4	0.0147	86.6	235.7
F–F---π(^{a}C) (C_s:IA_{Cor})	0.0127	0.0068	0.0018	-0.852	0.0071	75.5	65.7	0.0422	77.9	139.2
Cl–Cl---π(^{a}C) (C_s:IA_{Cor})	0.0170	0.0071	0.0012	-0.911	0.0072	80.7	69.9	0.0354	102.3	105.6
Br–Br---π(^{a}C) (C_s:IA_{Cor})	0.0173	0.0064	0.0007	-0.939	0.0064	83.4	57.2	0.0232	107.7	140.4
I–I---π(^{a}C) (C_s:IA_{Cor})	0.0166	0.0052	0.0003	-0.973	0.0052	87.0	52.0	0.0172	114.2	195.4
F–Cl---π(^{a}C) (C_s:IA_{Cor})	0.0194	0.0079	0.0009	-0.940	0.0079	83.5	79.5	0.0273	106.8	100.8
F–Br---π(^{a}C) (C_s:IA_{Cor})	0.0204	0.0074	0.0004	-0.973	0.0074	87.0	71.9	0.0315	116.4	144.8
F–I---π(^{a}C) (C_s:IA_{Cor})	0.0194	0.0060	-0.0001	-1.009	0.0060	91.0	67.0	0.0278	123.8	180.2

[a] Data are given at BCP, which is shown by X-∗-π. [b] $c\nabla^2\rho_b(\boldsymbol{r}_c) = H_b(\boldsymbol{r}_c) - V_b(\boldsymbol{r}_c)/2$, where $c = \hbar^2/8m$. [c] $k_b(\boldsymbol{r}_c) = V_b(\boldsymbol{r}_c)/G_b(\boldsymbol{r}_c)$. [d] mDyne Å^{-1}.

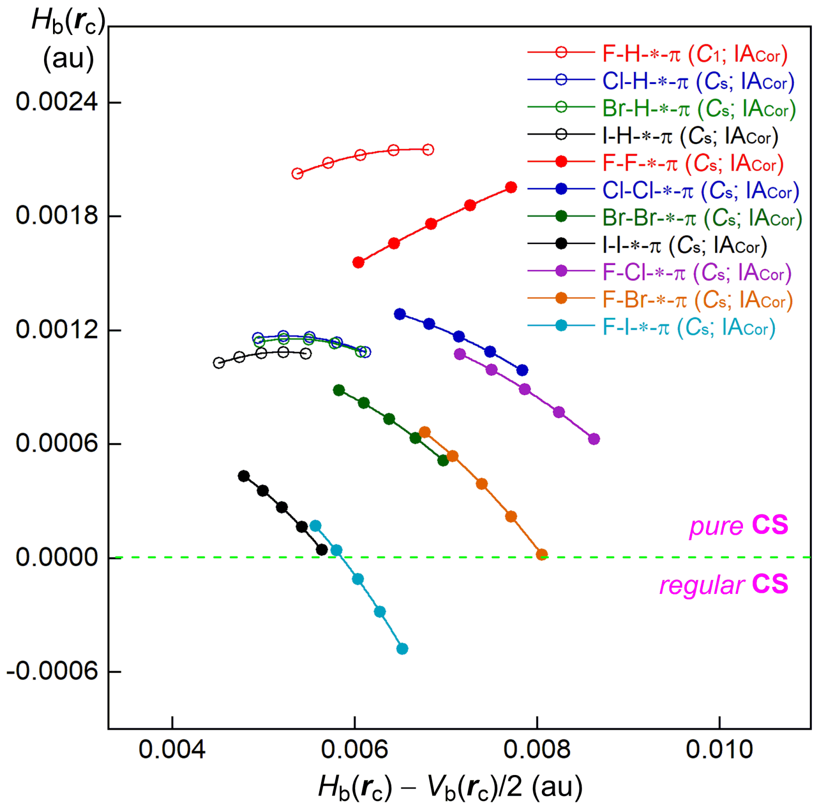

Fig. S13 QTAIM-DFA plots ($H_{b(rc)}$ versus $H_{b(rc)} - V_{b(rc)}/2$) for X–H-∗-π($C_{24}H_{12}$) and Y–X-∗-π($C_{24}H_{12}$) (X, Y = F, Cl, Br and I), evaluated with MP2/6-311G(d,p) for XH---π($C_{24}H_{12}$) and MP2/6-311G(d) for YX-∗-π($C_{24}H_{12}$).

References

S1 (a) *Atoms in Molecules. A Quantum Theory*: eds. R. F. W. Bader, Oxford University Press, Oxford, UK, 1990; (b) C. F. Matta, R. J. Boyd, *An Introduction to the Quantum Theory of Atoms in Molecules* in *The Quantum Theory of Atoms in Molecules: From Solid State to DNA and Drug Design*: eds. C. F. Matta, R. J. Boyd, WILEY-VCH, Weinheim, Germany, 2007, Chapter 1.

S2 (a) R. F. W. Bader, T. S. Slee. D. Cremer, E. Kraka, *J. Am. Chem. Soc.* **1983**, *105*, 5061–5068; (b) R. F. W. Bader, *Chem. Rev.* **1991**, *91*, 893–926; (c) R. F. W. Bader, *J. Phys. Chem. A* **1998**, *102*, 7314–7323; (d) F. Biegler-König, R. F. W. Bader, T. H. Tang, *J. Comput. Chem.* **1982**, *3*, 317–328; (e) R. F. W. Bader, *Acc. Chem. Res.* **1985**, *18*, 9–15; (f) T. H. Tang, R. F. W. Bader, P. MacDougall, *Inorg. Chem.* **1985**, *24*, 2047–2053; (g) F. Biegler-König, J. Schönbohm, D. Bayles, *J. Comput. Chem.* **2001**, *22*, 545–559; (h) F. Biegler-König, J. Schönbohm, *J. Comput. Chem.* **2002**, *23*, 1489–1494.

S3 W. Nakanishi, T. Nakamoto, S. Hayashi, T. Sasamori, N. Tokitoh, *Chem. Eur. J.* **2007**, *13*, 255–268.

S4 (a) W. Nakanishi, S. Hayashi, K. Narahara, *J. Phys. Chem. A* **2009**, *113*, 10050–10057; (*b*) W. Nakanishi, S. Hayashi, K. Narahara, *J. Phys. Chem. A* **2008**, *112*, 13593–13599.

S5 W. Nakanishi, S. Hayashi, *Curr. Org. Chem.* **2010**, *14*, 181–197.

S6 (a) W. Nakanishi, S. Hayashi, *J. Phys. Chem. A* **2010**, *114*, 7423–7430; (b) W. Nakanishi, S. Hayashi, K. Matsuiwa, M. Kitamoto, *Bull. Chem. Soc. Jpn* **2012**, *85*, 1293–1305.

Optimized structures given by Cartesian coordinates

The structures were optimized using the Gaussian 09 programme package.[62] The basis set system (BSS) from the Sapporo Basis Set Factory[63] (BSS-S) was employed for the calculations. In the calculations with BSS-SA, the (7433211/743111/7411/2 + 1s1p) type was employed for I, the (743211/74111/721/2 + 1s1p) type for Br, the (63211/6111/31/2 + 1s1p) type for Cl and the (6211/311/21/2 + 1s1p) type for F with the (6211/311/21/2 + 1s1p) type for C and the (411/21/2 + 1s1p) type for H. BSS-SA was applied for the calculations at the M06-2X (M06-2X/BSS-SA) level of density functional theory (DFT). Optimized structures were confirmed by the frequency analysis. QTAIM functions were similarly calculated using the Gaussian 09 programme package[62] with the same method of the optimizations and the data were analysed with the AIM2000 [64] and AIMAll[65] programmes. The results obtained at the M06-2X/BSS-SA level of theory will be mainly discussed in the text.

For BSS-SB, the (743321/74321/742 + 1s1p) type was employed for I, the (74321/7421/72 + 1s1p) type for Br, the (6321/621/3 + 1s1p) type for Cl and the (621/31/2 + 1s1p) type for F with the (621/31/2 + 1s1p) type for C and the (31/3 + 1s1p) type for H. The calculations were also performed at the M06-2X/BSS-SB level of theory to search for the potential energy surface minima as the pre-optimizations, when necessary. M06-2X/BSS-SB is also employed to confirm the minima and BPs with BCPs around the interactions in question, if they are not obtained satisfactorily with M06-2X/BSS-SA.

M06-2X/BSS-SA
Adduct F–H---π($C_{24}H_{12}$)
Symmetry C_1: type IB_{Cor}
energy HF = -1022.1894438
Standard orientation

6	0	-0.543222	-1.232282	-0.237685
6	0	-1.250662	0.000000	-0.305076
6	0	-0.543221	1.232282	-0.237684
6	0	0.872511	1.231079	-0.110515
6	0	1.579867	-0.000001	-0.048621
6	0	0.872509	-1.231082	-0.110515
6	0	-1.244793	-2.449727	-0.293380
6	0	-2.659422	-2.422573	-0.416174
6	0	-3.338837	-1.245098	-0.483474
1	0	-3.194005	-3.363285	-0.453063
1	0	-4.417571	-1.239048	-0.573229
6	0	-2.654759	0.000001	-0.431329
6	0	-3.338835	1.245101	-0.483474
6	0	-2.659420	2.422574	-0.416173
1	0	-4.417569	1.239053	-0.573229
1	0	-3.194001	3.363288	-0.453062
6	0	-1.244791	2.449728	-0.293379
6	0	-0.515331	3.665300	-0.222615
6	0	0.840799	3.664279	-0.104527
1	0	-1.060505	4.599743	-0.263953
1	0	1.385025	4.598883	-0.051861
6	0	1.572130	2.449072	-0.044959
6	0	2.985999	2.421879	0.081315
6	0	3.663747	1.242785	0.139812

1	0	3.519638	3.362627	0.132255
1	0	4.742013	1.236360	0.237375
6	0	2.980656	-0.000001	0.077353
6	0	3.663746	-1.242787	0.139812
6	0	2.986000	-2.421881	0.081315
1	0	4.742013	-1.236360	0.237375
1	0	3.519637	-3.362629	0.132256
6	0	1.572131	-2.449073	-0.044959
6	0	0.840796	-3.664277	-0.104527
6	0	-0.515334	-3.665300	-0.222615
1	0	1.385013	-4.598884	-0.051859
1	0	-1.060507	-4.599742	-0.263953
1	0	-2.467605	0.000000	1.821826
9	0	-2.557240	0.000000	2.741730

M06-2X/BSS-SA
Adduct Br–H---π($C_{24}H_{12}$)
Symmetry C_1: type IB'$_{Cor}$
energy HF = -3496.5367492
Standard orientation

6	0	-0.593765	-0.564601	-0.942359
6	0	-0.439972	0.847901	-0.885030
6	0	0.786958	1.412257	-0.439736
6	0	1.861202	0.564134	-0.057775
6	0	1.707215	-0.847828	-0.114261
6	0	0.478865	-1.412075	-0.552952
6	0	-1.811078	-1.123487	-1.372792
6	0	-2.870163	-0.255349	-1.746256
6	0	-2.724142	1.098663	-1.691187
1	0	-3.806717	-0.690226	-2.071934
1	0	-3.542562	1.748560	-1.974150
6	0	-1.505934	1.688177	-1.258419
6	0	-1.330910	3.095630	-1.181727
6	0	-0.154844	3.634300	-0.759083
1	0	-2.155405	3.736241	-1.468114
1	0	-0.034081	4.708975	-0.706029
6	0	0.936194	2.809240	-0.377422
6	0	2.171732	3.347695	0.067684
6	0	3.201991	2.535120	0.431139
1	0	2.281347	4.423833	0.114268
1	0	4.138959	2.958908	0.770421
6	0	3.075909	1.122319	0.379316
6	0	4.133985	0.253798	0.755643
6	0	3.986658	-1.098369	0.701075
1	0	5.066934	0.688851	1.091649
1	0	4.801399	-1.749190	0.992615
6	0	2.769780	-1.686664	0.266459
6	0	2.588779	-3.093189	0.205885
6	0	1.410227	-3.633597	-0.209354
1	0	3.410350	-3.733961	0.500537
1	0	1.284648	-4.708307	-0.247755
6	0	0.323034	-2.808833	-0.599782
6	0	-0.916511	-3.347277	-1.035566
6	0	-1.944282	-2.536330	-1.407277
1	0	-1.031512	-4.423428	-1.065700
1	0	-2.884967	-2.959869	-1.735932
1	0	-2.483336	0.519782	0.872801
35	0	-2.739732	-0.015142	2.167714

M06-2X/BSS-SA
Adduct I–H---π($C_{24}H_{12}$)
Symmetry C_1: type IB_{Cor}
energy HF = -7843.2534562
Standard orientation

6	0	0.310216	-0.411812	1.259465
6	0	0.023718	0.972315	1.107672
6	0	-1.181516	1.384343	0.475548
6	0	-2.102917	0.411826	0.001088
6	0	-1.816043	-0.971949	0.151976
6	0	-0.608303	-1.383693	0.777931
6	0	1.512858	-0.819579	1.864768
6	0	2.423573	0.170711	2.316510
6	0	2.149882	1.497482	2.172419
1	0	3.350494	-0.149332	2.775647
1	0	2.855750	2.241870	2.518944
6	0	0.943243	1.935749	1.563960
6	0	0.640085	3.312084	1.388288
6	0	-0.516786	3.705130	0.788368
1	0	1.351074	4.047866	1.742598
1	0	-0.734887	4.757926	0.660143
6	0	-1.458715	2.753726	0.314713
6	0	-2.669252	3.138893	-0.318373
6	0	-3.554379	2.207287	-0.767937
1	0	-2.877125	4.194616	-0.439264
1	0	-4.473796	2.515278	-1.250023
6	0	-3.295830	0.819287	-0.622728
6	0	-4.198922	-0.171441	-1.089833
6	0	-3.923735	-1.496889	-0.946209
1	0	-5.116047	0.148494	-1.568462
1	0	-4.620180	-2.241828	-1.310097
6	0	-2.724880	-1.934054	-0.323838
6	0	-2.411376	-3.309532	-0.168230
6	0	-1.251473	-3.703812	0.425745
1	0	-3.114993	-4.045414	-0.536813
1	0	-1.023636	-4.756739	0.534457
6	0	-0.317463	-2.752714	0.913661
6	0	0.901943	-3.137660	1.530601
6	0	1.783849	-2.207342	1.987755
1	0	1.120891	-4.193496	1.629083
1	0	2.712475	-2.515165	2.451667
1	0	2.171854	0.928069	-0.547596
53	0	2.574556	-0.018533	-1.801513

M06-2X/BSS-SA
Adduct F–H---π($C_{24}H_{12}$)
Symmetry C_s: type IC_{Cor}
energy HF = -1022.1901607
Standard orientation

6	0	0.294299	-1.003853	0.711265
6	0	0.294299	-1.003853	-0.711265
6	0	0.157335	0.219644	-1.421537
6	0	0.022746	1.442681	-0.710659
6	0	0.022746	1.442681	0.710659
6	0	0.157335	0.219644	1.421537

6	0	0.428357	-2.213221	1.417056
6	0	0.563828	-3.422450	0.682741
6	0	0.563828	-3.422450	-0.682741
1	0	0.668892	-4.352381	1.227766
1	0	0.668892	-4.352381	-1.227766
6	0	0.428357	-2.213221	-1.417056
6	0	0.420710	-2.188279	-2.835940
6	0	0.289986	-1.015980	-3.514886
1	0	0.520052	-3.123937	-3.371452
1	0	0.285102	-1.009355	-4.597445
6	0	0.155731	0.218937	-2.827983
6	0	0.018674	1.453970	-3.514173
6	0	-0.109668	2.625540	-2.833062
1	0	0.017072	1.447472	-4.596750
1	0	-0.214207	3.560796	-3.368637
6	0	-0.111123	2.653148	-1.413733
6	0	-0.243748	3.861437	-0.680703
6	0	-0.243748	3.861437	0.680703
1	0	-0.346325	4.790581	-1.227128
1	0	-0.346325	4.790581	1.227128
6	0	-0.111123	2.653148	1.413733
6	0	-0.109668	2.625540	2.833062
6	0	0.018674	1.453970	3.514173
1	0	-0.214207	3.560796	3.368637
1	0	0.017072	1.447472	4.596750
6	0	0.155731	0.218937	2.827983
6	0	0.289986	-1.015980	3.514886
6	0	0.420710	-2.188279	2.835940
1	0	0.285102	-1.009355	4.597445
1	0	0.520052	-3.123937	3.371452
1	0	-1.617500	-3.397935	0.000000
9	0	-2.543246	-3.423034	0.000000

M06-2X/BSS-SA
Adduct Cl–H---π($C_{24}H_{12}$)
Symmetry C_1: type IC_{Cor}
energy HF = -1382.5044292
Standard orientation

6	0	0.909941	0.711048	-0.528605
6	0	0.909975	-0.710989	-0.528613
6	0	-0.301726	-1.421489	-0.310672
6	0	-1.513747	-0.710734	-0.098690
6	0	-1.513782	0.710674	-0.098685
6	0	-0.301794	1.421489	-0.310660
6	0	2.110146	1.415961	-0.734720
6	0	3.308781	0.682096	-0.939455
6	0	3.308813	-0.681918	-0.939465
1	0	4.231526	1.227468	-1.092963
1	0	4.231583	-1.227244	-1.092981
6	0	2.110213	-1.415842	-0.734738
6	0	2.086136	-2.835215	-0.719005
6	0	0.925079	-3.514359	-0.512464
1	0	3.014305	-3.370570	-0.874246
1	0	0.919753	-4.596939	-0.502589
6	0	-0.299962	-2.827775	-0.303696
6	0	-1.523192	-3.513999	-0.086545
6	0	-2.685037	-2.833035	0.113002
1	0	-1.515942	-4.596596	-0.082021

1	0	-3.611597	-3.368652	0.277589
6	0	-2.712993	-1.413915	0.113484
6	0	-3.910393	-0.680743	0.322997
6	0	-3.910426	0.680565	0.323001
1	0	-4.830965	-1.227106	0.485780
1	0	-4.831025	1.226883	0.485785
6	0	-2.713062	1.413796	0.113492
6	0	-2.685174	2.832917	0.113019
6	0	-1.523361	3.513939	-0.086521
1	0	-3.611759	3.368489	0.277606
1	0	-1.516162	4.596536	-0.081989
6	0	-0.300097	2.827775	-0.303673
6	0	0.924912	3.514419	-0.512430
6	0	2.086002	2.835332	-0.718973
1	0	0.919534	4.596999	-0.502546
1	0	3.014147	3.370733	-0.874207
1	0	2.833655	0.000031	1.464215
17	0	2.590672	-0.000002	2.724956

M06-2X/BSS-SA
Adduct Br–H---π($C_{24}H_{12}$)
Symmetry C_S: type IC_{Cor}
energy HF = -3496.5368922
Standard orientation

6	0	0.907044	-0.614496	0.710813
6	0	0.907044	-0.614496	-0.710813
6	0	0.525980	0.556080	-1.421456
6	0	0.149746	1.727793	-0.710553
6	0	0.149746	1.727793	0.710553
6	0	0.525980	0.556080	1.421456
6	0	1.265335	-1.778312	1.415918
6	0	1.619461	-2.941491	0.681903
6	0	1.619461	-2.941491	-0.681903
1	0	1.889084	-3.836796	1.228028
1	0	1.889084	-3.836796	-1.228028
6	0	1.265335	-1.778312	-1.415918
6	0	1.244268	-1.757254	-2.835087
6	0	0.884524	-0.634127	-3.514336
1	0	1.518590	-2.656960	-3.371276
1	0	0.871444	-0.630872	-4.596906
6	0	0.515166	0.552243	-2.827489
6	0	0.131482	1.733911	-3.513862
6	0	-0.224766	2.857747	-2.833104
1	0	0.125832	1.725324	-4.596468
1	0	-0.515571	3.752226	-3.369589
6	0	-0.227328	2.885987	-1.414035
6	0	-0.600106	4.043128	-0.680653
6	0	-0.600106	4.043128	0.680653
1	0	-0.887900	4.932129	-1.227726
1	0	-0.887900	4.932129	1.227726
6	0	-0.227328	2.885987	1.414035
6	0	-0.224766	2.857747	2.833104
6	0	0.131482	1.733911	3.513862
1	0	-0.515571	3.752226	3.369589
1	0	0.125832	1.725324	4.596468
6	0	0.515166	0.552243	2.827489
6	0	0.884524	-0.634127	3.514336
6	0	1.244268	-1.757254	2.835087

1	0	0.871444	-0.630872	4.596906
1	0	1.518590	-2.656960	3.371276
1	0	-0.898423	-2.772006	0.000000
35	0	-2.268406	-2.382075	0.000000

M06-2X/BSS-SA
Adduct I–H---π($C_{24}H_{12}$)
Symmetry C_S: type IC_{Cor}
energy HF = -7843.2536265
Standard orientation

6	0	1.212926	-0.260955	0.710792
6	0	1.212926	-0.260955	-0.710792
6	0	0.656232	0.836910	-1.421337
6	0	0.106398	1.937803	-0.710546
6	0	0.106398	1.937803	0.710546
6	0	0.656232	0.836910	1.421337
6	0	1.744510	-1.356580	1.415276
6	0	2.274346	-2.450745	0.681785
6	0	2.274346	-2.450745	-0.681785
1	0	2.678644	-3.294081	1.227781
1	0	2.678644	-3.294081	-1.227781
6	0	1.744510	-1.356580	-1.415276
6	0	1.717094	-1.340632	-2.834360
6	0	1.188061	-0.286708	-3.513963
1	0	2.125697	-2.188358	-3.370004
1	0	1.172218	-0.287013	-4.596513
6	0	0.643698	0.830353	-2.827439
6	0	0.084214	1.939764	-3.513695
6	0	-0.436534	2.997401	-2.832774
1	0	0.078224	1.929687	-4.596294
1	0	-0.860938	3.837346	-3.368523
6	0	-0.441883	3.025374	-1.413761
6	0	-0.984947	4.112953	-0.680659
6	0	-0.984947	4.112953	0.680659
1	0	-1.404806	4.948081	-1.227355
1	0	-1.404806	4.948081	1.227355
6	0	-0.441883	3.025374	1.413761
6	0	-0.436534	2.997401	2.832774
6	0	0.084214	1.939764	3.513695
1	0	-0.860938	3.837346	3.368523
1	0	0.078224	1.929687	4.596294
6	0	0.643698	0.830353	2.827439
6	0	1.188061	-0.286708	3.513963
6	0	1.717094	-1.340632	2.834360
1	0	1.172218	-0.287013	4.596513
1	0	2.125697	-2.188358	3.370004
1	0	-0.299473	-2.688130	0.000000
53	0	-1.895245	-2.396650	0.000000

M06-2X/BSS-SA
Adduct F–F---π($C_{24}H_{12}$)
Symmetry C_S: type IA_{Cor}
energy HF = -1121.2402556
Standard orientation

6	0	0.433409	-0.512450	1.231375
6	0	0.522775	-1.217784	0.000000

6	0	0.433409	-0.512450	-1.231375
6	0	0.257444	0.898185	-1.231172
6	0	0.170215	1.603422	0.000000
6	0	0.257444	0.898185	1.231172
6	0	0.509522	-1.211721	2.449167
6	0	0.675200	-2.621535	2.421459
6	0	0.759003	-3.297301	1.242653
1	0	0.732528	-3.154447	3.362367
1	0	0.882666	-4.372906	1.236429
6	0	0.684591	-2.615673	0.000000
6	0	0.759003	-3.297301	-1.242653
6	0	0.675200	-2.621535	-2.421459
1	0	0.882666	-4.372906	-1.236429
1	0	0.732528	-3.154447	-3.362367
6	0	0.509522	-1.211721	-2.449167
6	0	0.415500	-0.484484	-3.664671
6	0	0.251450	0.867036	-3.664664
1	0	0.476060	-1.027856	-4.599354
1	0	0.181257	1.409070	-4.599469
6	0	0.166486	1.595567	-2.449252
6	0	-0.009613	3.004059	-2.421785
6	0	-0.092130	3.679585	-1.242762
1	0	-0.078958	3.535460	-3.362738
1	0	-0.227339	4.753848	-1.236399
6	0	-0.005536	2.999126	0.000000
6	0	-0.092130	3.679585	1.242762
6	0	-0.009613	3.004059	2.421785
1	0	-0.227339	4.753848	1.236399
1	0	-0.078958	3.535460	3.362738
6	0	0.166486	1.595567	2.449252
6	0	0.251450	0.867036	3.664664
6	0	0.415500	-0.484484	3.664671
1	0	0.181257	1.409070	4.599469
1	0	0.476060	-1.027856	4.599354
9	0	-2.239134	-1.593413	0.000000
9	0	-3.600860	-1.729274	0.000000

M06-2X/BSS-SA
Adduct Cl–Cl---π($C_{24}H_{12}$)
Symmetry C_1: type IA_{Cor}
energy HF = -1842.0240531
Standard orientation

6	0	-0.169076	-1.232211	-0.840413
6	0	-0.836318	-0.000017	-1.087553
6	0	-0.169117	1.232198	-0.840412
6	0	1.163991	1.231427	-0.349131
6	0	1.831059	0.000026	-0.104406
6	0	1.164031	-1.231396	-0.349133
6	0	-0.834021	-2.449811	-1.071539
6	0	-2.170400	-2.421636	-1.549243
6	0	-2.809884	-1.243050	-1.784912
1	0	-2.677947	-3.362662	-1.720401
1	0	-3.831441	-1.236892	-2.143451
6	0	-2.161774	-0.000039	-1.561255
6	0	-2.809925	1.242951	-1.784911
6	0	-2.170479	2.421558	-1.549240
1	0	-3.831482	1.236762	-2.143450
1	0	-2.678058	3.362568	-1.720396

6	0	-0.834102	2.449777	-1.071535
6	0	-0.147987	3.665343	-0.813395
6	0	1.131104	3.665036	-0.347506
1	0	-0.663955	4.600081	-0.993489
1	0	1.642474	4.599832	-0.154764
6	0	1.822144	2.449636	-0.101428
6	0	3.154477	2.422143	0.386996
6	0	3.794089	1.242972	0.619116
1	0	3.656963	3.363105	0.572926
1	0	4.811090	1.236580	0.990752
6	0	3.150218	0.000047	0.382233
6	0	3.794130	-1.242855	0.619114
6	0	3.154558	-2.422048	0.386991
1	0	4.811131	-1.236433	0.990750
1	0	3.657074	-3.362993	0.572920
6	0	1.822225	-2.449584	-0.101432
6	0	1.131225	-3.665006	-0.347512
6	0	-0.147866	-3.665355	-0.813401
1	0	1.642624	-4.599786	-0.154773
1	0	-0.663805	-4.600110	-0.993497
17	0	-1.933951	-0.000034	1.745276
17	0	-2.594783	-0.000007	3.627684

M06-2X/BSS-SA
Adduct Br–Br---π($C_{24}H_{12}$)
Symmetry C_s: type IA_{Cor}
energy HF = -6070.120944
Standard orientation

6	0	-1.094074	-0.803246	1.232546
6	0	-1.620220	-0.324215	0.000000
6	0	-1.094074	-0.803246	-1.232546
6	0	-0.044864	-1.761656	-1.231465
6	0	0.478885	-2.241365	0.000000
6	0	-0.044864	-1.761656	1.231465
6	0	-1.604321	-0.318201	2.450053
6	0	-2.643691	0.648583	2.421596
6	0	-3.147770	1.107602	1.243233
1	0	-3.029705	1.021009	3.362156
1	0	-3.937859	1.847859	1.237110
6	0	-2.650977	0.635542	0.000000
6	0	-3.147770	1.107602	-1.243233
6	0	-2.643691	0.648583	-2.421596
1	0	-3.937859	1.847859	-1.237110
1	0	-3.029705	1.021009	-3.362156
6	0	-1.604321	-0.318201	-2.450053
6	0	-1.059138	-0.808333	-3.665609
6	0	-0.057759	-1.730654	-3.665077
1	0	-1.455634	-0.431588	-4.600052
1	0	0.349142	-2.096243	-4.599560
6	0	0.477757	-2.232001	-2.449622
6	0	1.525345	-3.189513	-2.421999
6	0	2.026395	-3.649569	-1.242856
1	0	1.923146	-3.549501	-3.362526
1	0	2.827081	-4.378562	-1.236218
6	0	1.518888	-3.187879	0.000000
6	0	2.026395	-3.649569	1.242856
6	0	1.525345	-3.189513	2.421999
1	0	2.827081	-4.378562	1.236218
1	0	1.923146	-3.549501	3.362526

6	0	0.477757	-2.232001	2.449622
6	0	-0.057759	-1.730654	3.665077
6	0	-1.059138	-0.808333	3.665609
1	0	0.349142	-2.096243	4.599560
1	0	-1.455634	-0.431588	4.600052
35	0	0.451647	2.020905	0.000000
35	0	2.055600	3.656964	0.000000

M06-2X/BSS-SA
Adduct I–I---π($C_{24}H_{12}$)
Symmetry C_s: type IA_{Cor}
energy HF = -14763.5905215
Standard orientation

6	0	-0.987065	-1.617652	1.232412
6	0	-1.621020	-1.294474	0.000000
6	0	-0.987065	-1.617652	-1.232412
6	0	0.276260	-2.269368	-1.231321
6	0	0.906663	-2.595967	0.000000
6	0	0.276260	-2.269368	1.231321
6	0	-1.603438	-1.277772	2.449810
6	0	-2.856601	-0.611215	2.421567
6	0	-3.463226	-0.299906	1.242980
1	0	-3.323457	-0.347061	3.362168
1	0	-4.415196	0.215703	1.237119
6	0	-2.863326	-0.630979	0.000000
6	0	-3.463226	-0.299906	-1.242980
6	0	-2.856601	-0.611215	-2.421567
1	0	-4.415196	0.215703	-1.237119
1	0	-3.323457	-0.347061	-3.362168
6	0	-1.603438	-1.277772	-2.449810
6	0	-0.946548	-1.603137	-3.665244
6	0	0.261119	-2.231541	-3.664638
1	0	-1.425441	-1.338424	-4.599545
1	0	0.752228	-2.472587	-4.599062
6	0	0.906294	-2.580722	-2.449376
6	0	2.167504	-3.231494	-2.422031
6	0	2.769422	-3.548669	-1.242798
1	0	2.647249	-3.471193	-3.362694
1	0	3.732084	-4.044464	-1.236526
6	0	2.158257	-3.237536	0.000000
6	0	2.769422	-3.548669	1.242798
6	0	2.167504	-3.231494	2.422031
1	0	3.732084	-4.044464	1.236526
1	0	2.647249	-3.471193	3.362694
6	0	0.906294	-2.580722	2.449376
6	0	0.261119	-2.231541	3.664638
6	0	-0.946548	-1.603137	3.665244
1	0	0.752228	-2.472587	4.599062
1	0	-1.425441	-1.338424	4.599545
53	0	-0.085120	1.639368	0.000000
53	0	1.109591	4.034735	0.000000

M06-2X/BSS-SA
Adduct F–Cl---π($C_{24}H_{12}$)
Symmetry C_s: type IA_{Cor}
energy HF = -7942.4669678

Standard orientation

6	0	0.995086	0.202916	1.233877
6	0	1.304361	-0.438677	0.000000
6	0	0.995086	0.202916	-1.233877
6	0	0.370199	1.479152	-1.231781
6	0	0.059390	2.117027	0.000000
6	0	0.370199	1.479152	1.231781
6	0	1.295781	-0.434178	2.450657
6	0	1.912162	-1.713078	2.421580
6	0	2.213665	-2.325635	1.243741
1	0	2.135760	-2.200272	3.362280
1	0	2.678348	-3.303350	1.238150
6	0	1.920516	-1.707059	0.000000
6	0	2.213665	-2.325635	-1.243741
6	0	1.912162	-1.713078	-2.421580
1	0	2.678348	-3.303350	-1.238150
1	0	2.135760	-2.200272	-3.362280
6	0	1.295781	-0.434178	-2.450657
6	0	0.967834	0.221076	-3.666164
6	0	0.372178	1.445185	-3.665146
1	0	1.199359	-0.274269	-4.600569
1	0	0.126421	1.933827	-4.599582
6	0	0.057321	2.107964	-2.449889
6	0	-0.566374	3.382779	-2.422367
6	0	-0.863846	3.994238	-1.242954
1	0	-0.804559	3.863473	-3.362858
1	0	-1.341080	4.966219	-1.236567
6	0	-0.561445	3.378725	0.000000
6	0	-0.863846	3.994238	1.242954
6	0	-0.566374	3.382779	2.422367
1	0	-1.341080	4.966219	1.236567
1	0	-0.804559	3.863473	3.362858
6	0	0.057321	2.107964	2.449889
6	0	0.372178	1.445185	3.665146
6	0	0.967834	0.221076	3.666164
1	0	0.126421	1.933827	4.599582
1	0	1.199359	-0.274269	4.600569
53	0	-1.452495	-1.973693	0.000000
9	0	-3.154587	-2.865628	0.000000

M06-2X/BSS-SA

Adduct F–Br---π($C_{24}H_{12}$)

Symmetry C_s: type IA_{Cor}

energy HF = -3595.7155633

Standard orientation

6	0	0.829569	-0.126171	1.233731
6	0	1.043591	-0.805389	0.000000
6	0	0.829569	-0.126171	-1.233731
6	0	0.390772	1.224856	-1.231786
6	0	0.170580	1.899801	0.000000
6	0	0.390772	1.224856	1.231786
6	0	1.039699	-0.798490	2.450584
6	0	1.464970	-2.152941	2.421679
6	0	1.669773	-2.804169	1.243917
1	0	1.619788	-2.665323	3.362832
1	0	1.988415	-3.838746	1.238327
6	0	1.466506	-2.150150	0.000000
6	0	1.669773	-2.804169	-1.243917

6	0	1.464970	-2.152941	-2.421679
1	0	1.988415	-3.838746	-1.238327
1	0	1.619788	-2.665323	-3.362832
6	0	1.039699	-0.798490	-2.450584
6	0	0.810466	-0.102367	-3.666138
6	0	0.391466	1.192692	-3.665267
1	0	0.973005	-0.623816	-4.600934
1	0	0.218319	1.710909	-4.600056
6	0	0.169257	1.891935	-2.449963
6	0	-0.270229	3.241286	-2.422308
6	0	-0.479698	3.888214	-1.242980
1	0	-0.437765	3.750203	-3.363171
1	0	-0.814683	4.917845	-1.236456
6	0	-0.266694	3.236054	0.000000
6	0	-0.479698	3.888214	1.242980
6	0	-0.270229	3.241286	2.422308
1	0	-0.814683	4.917845	1.236456
1	0	-0.437765	3.750203	3.363171
6	0	0.169257	1.891935	2.449963
6	0	0.391466	1.192692	3.665267
6	0	0.810466	-0.102367	3.666138
1	0	0.218319	1.710909	4.600056
1	0	0.973005	-0.623816	4.600934
35	0	-1.782830	-1.839230	0.000000
9	0	-3.485724	-2.296571	0.000000

M06-2X/BSS-SA
Adduct F–I---π($C_{24}H_{12}$)
Symmetry C_s: type IA_{Cor}
energy HF = =-7942.4669678
Standard orientation

6	0	0.995086	0.202916	1.233877
6	0	1.304361	-0.438677	0.000000
6	0	0.995086	0.202916	-1.233877
6	0	0.370199	1.479152	-1.231781
6	0	0.059390	2.117027	0.000000
6	0	0.370199	1.479152	1.231781
6	0	1.295781	-0.434178	2.450657
6	0	1.912162	-1.713078	2.421580
6	0	2.213665	-2.325635	1.243741
1	0	2.135760	-2.200272	3.362280
1	0	2.678348	-3.303350	1.238150
6	0	1.920516	-1.707059	0.000000
6	0	2.213665	-2.325635	-1.243741
6	0	1.912162	-1.713078	-2.421580
1	0	2.678348	-3.303350	-1.238150
1	0	2.135760	-2.200272	-3.362280
6	0	1.295781	-0.434178	-2.450657
6	0	0.967834	0.221076	-3.666164
6	0	0.372178	1.445185	-3.665146
1	0	1.199359	-0.274269	-4.600569
1	0	0.126421	1.933827	-4.599582
6	0	0.057321	2.107964	-2.449889
6	0	-0.566374	3.382779	-2.422367
6	0	-0.863846	3.994238	-1.242954
1	0	-0.804559	3.863473	-3.362858
1	0	-1.341080	4.966219	-1.236567
6	0	-0.561445	3.378725	0.000000

6	0	-0.863846	3.994238	1.242954
6	0	-0.566374	3.382779	2.422367
1	0	-1.341080	4.966219	1.236567
1	0	-0.804559	3.863473	3.362858
6	0	0.057321	2.107964	2.449889
6	0	0.372178	1.445185	3.665146
6	0	0.967834	0.221076	3.666164
1	0	0.126421	1.933827	4.599582
1	0	1.199359	-0.274269	4.600569
53	0	-1.452495	-1.973693	0.000000
9	0	-3.154587	-2.865628	0.000000

M06-2X/BSS-SA
Adduct F–F---π($C_{24}H_{12}$)
Symmetry C_s: type IC_{Cor}
energy HF = -1121.2400649
Standard orientation

6	0	0.504383	-0.912005	0.710976
6	0	0.504383	-0.912005	-0.710976
6	0	0.300055	0.301913	-1.421477
6	0	0.096828	1.516138	-0.710800
6	0	0.096828	1.516138	0.710800
6	0	0.300055	0.301913	1.421477
6	0	0.704687	-2.113026	1.414586
6	0	0.900454	-3.312698	0.681098
6	0	0.900454	-3.312698	-0.681098
1	0	1.050511	-4.235776	1.227052
1	0	1.050511	-4.235776	-1.227052
6	0	0.704687	-2.113026	-1.414586
6	0	0.697548	-2.086899	-2.833521
6	0	0.502353	-0.924025	-3.514220
1	0	0.850944	-3.015491	-3.368958
1	0	0.499347	-0.918109	-4.596917
6	0	0.298500	0.301662	-2.828213
6	0	0.092774	1.527443	-3.514129
6	0	-0.100901	2.690258	-2.833380
1	0	0.092641	1.521051	-4.596830
1	0	-0.256730	3.618459	-3.368830
6	0	-0.104552	2.717410	-1.413953
6	0	-0.305283	3.916085	-0.680749
6	0	-0.305283	3.916085	0.680749
1	0	-0.460245	4.838113	-1.227025
1	0	-0.460245	4.838113	1.227025
6	0	-0.104552	2.717410	1.413953
6	0	-0.100901	2.690258	2.833380
6	0	0.092774	1.527443	3.514129
1	0	-0.256730	3.618459	3.368830
1	0	0.092641	1.521051	4.596830
6	0	0.298500	0.301662	2.828213
6	0	0.502353	-0.924025	3.514220
6	0	0.697548	-2.086899	2.833521
1	0	0.499347	-0.918109	4.596917
1	0	0.850944	-3.015491	3.368958
9	0	-1.972938	-2.913340	0.000000
9	0	-3.204293	-2.318168	0.000000

M06-2X/BSS-SA
Adduct Cl–Cl---π($C_{24}H_{12}$)
Symmetry C_1: type IC_{Cor}
energy HF = -1842.0234094
Standard orientation

6	0	-0.261876	-0.711042	-0.910703
6	0	-0.261876	0.711045	-0.910700
6	0	0.857573	1.421513	-0.398830
6	0	1.978277	0.710597	0.110354
6	0	1.978277	-0.710596	0.110351
6	0	0.857574	-1.421511	-0.398836
6	0	-1.369938	-1.415500	-1.414170
6	0	-2.478174	-0.681896	-1.915002
6	0	-2.478174	0.681903	-1.915000
1	0	-3.332229	-1.227659	-2.296789
1	0	-3.332229	1.227667	-2.296784
6	0	-1.369939	1.415505	-1.414165
6	0	-1.349032	2.833947	-1.397329
6	0	-0.276361	3.514283	-0.906918
1	0	-2.207484	3.369507	-1.783006
1	0	-0.272697	4.596929	-0.899344
6	0	0.855883	2.827968	-0.395739
6	0	1.986949	3.514089	0.119732
6	0	3.060681	2.833196	0.606020
1	0	1.980235	4.596754	0.119478
1	0	3.916900	3.369000	0.996505
6	0	3.086627	1.413799	0.615282
6	0	4.193412	0.680756	1.117653
6	0	4.193412	-0.680759	1.117650
1	0	5.044404	1.227501	1.504330
1	0	5.044404	-1.227507	1.504324
6	0	3.086628	-1.413801	0.615276
6	0	3.060682	-2.833198	0.606009
6	0	1.986950	-3.514089	0.119717
1	0	3.916901	-3.369003	0.996491
1	0	1.980237	-4.596754	0.119459
6	0	0.855883	-2.827966	-0.395750
6	0	-0.276360	-3.514280	-0.906932
6	0	-1.349031	-2.833942	-1.397340
1	0	-0.272696	-4.596926	-0.899362
1	0	-2.207483	-3.369501	-1.783019
17	0	-3.592177	-0.000002	0.882013
17	0	-4.270561	-0.000006	2.762336

M06-2X/BSS-SA
Adduct Br–Br---π($C_{24}H_{12}$)
Symmetry C_1: type IC_{Cor}
energy HF = -6070.1200664
Standard orientation

6	0	0.715127	0.777522	1.091481
6	0	0.660949	-0.642828	1.149468
6	0	1.631869	-1.419371	0.461484
6	0	2.660629	-0.776855	-0.280031
6	0	2.716687	0.642267	-0.335303
6	0	1.742938	1.419411	0.348681
6	0	-0.248163	1.547825	1.766588
6	0	-1.266665	0.881770	2.499679
6	0	-1.317091	-0.480943	2.557344
1	0	-2.007228	1.477168	3.019350

1	0	-2.099419	-0.974886	3.120378
6	0	-0.355285	-1.279986	1.883746
6	0	-0.394158	-2.697539	1.918778
6	0	0.535863	-3.441365	1.258556
1	0	-1.183234	-3.181364	2.480503
1	0	0.494682	-4.522750	1.291920
6	0	1.574321	-2.823902	0.513777
6	0	2.556235	-3.576980	-0.182380
6	0	3.541706	-2.961615	-0.891469
1	0	2.506284	-4.657676	-0.139396
1	0	4.284292	-3.547744	-1.418273
6	0	3.621865	-1.545623	-0.959963
6	0	4.640045	-0.880201	-1.691797
6	0	4.694873	0.479226	-1.743337
1	0	5.377924	-1.477066	-2.213044
1	0	5.477400	0.974219	-2.304626
6	0	3.735179	1.277712	-1.068250
6	0	3.767866	2.696510	-1.108871
6	0	2.834112	3.441011	-0.455635
1	0	4.554903	3.180572	-1.673414
1	0	2.870533	4.522329	-0.496338
6	0	1.795491	2.823907	0.290433
6	0	0.809150	3.577158	0.978814
6	0	-0.176167	2.962698	1.689722
1	0	0.853960	4.657735	0.927973
1	0	-0.923959	3.548500	2.209295
35	0	-3.102793	0.095345	0.039288
35	0	-4.413504	-0.095285	-1.837957

M06-2X/BSS-SA
Adduct I–I---π($C_{24}H_{12}$)
Symmetry C_s: type IC_{Cor}
energy HF = -14763.5894163
Standard orientation

6	0	-1.236067	-1.440587	0.710998
6	0	-1.236067	-1.440587	-0.710998
6	0	-0.355550	-2.300687	-1.421561
6	0	0.521121	-3.164523	-0.710519
6	0	0.521121	-3.164523	0.710519
6	0	-0.355550	-2.300687	1.421561
6	0	-2.102686	-0.586051	1.416303
6	0	-2.968134	0.268487	0.682342
6	0	-2.968134	0.268487	-0.682342
1	0	-3.632004	0.927307	1.228702
1	0	-3.632004	0.927307	-1.228702
6	0	-2.102686	-0.586051	-1.416303
6	0	-2.076513	-0.596350	-2.834876
6	0	-1.232833	-1.420887	-3.514581
1	0	-2.742204	0.068172	-3.371182
1	0	-1.221865	-1.419530	-4.597152
6	0	-0.351313	-2.295850	-2.827756
6	0	0.535723	-3.165894	-3.514245
6	0	1.373234	-3.995556	-2.833462
1	0	0.534588	-3.157815	-4.596846
1	0	2.044581	-4.654274	-3.370034
6	0	1.390090	-4.017807	-1.414077
6	0	2.255675	-4.871201	-0.680748
6	0	2.255675	-4.871201	0.680748

1	0	2.921882	-5.526434	-1.227892
1	0	2.921882	-5.526434	1.227892
6	0	1.390090	-4.017807	1.414077
6	0	1.373234	-3.995556	2.833462
6	0	0.535723	-3.165894	3.514245
1	0	2.044581	-4.654274	3.370034
1	0	0.534588	-3.157815	4.596846
6	0	-0.351313	-2.295850	2.827756
6	0	-1.232833	-1.420887	3.514581
6	0	-2.076513	-0.596350	2.834876
1	0	-1.221865	-1.419530	4.597152
1	0	-2.742204	0.068172	3.371182
53	0	-0.470621	2.481610	0.000000
53	0	1.511321	4.283825	0.000000

M06-2X/BSS-SA
Adduct F–Cl---π($C_{24}H_{12}$)
Symmetry C_1: type IC_{Cor}
energy HF = -1481.6603611
Standard orientation

6	0	0.522201	0.711191	-0.673690
6	0	0.521821	-0.711862	-0.673574
6	0	-0.655581	-1.421614	-0.313999
6	0	-1.833208	-0.710171	0.043855
6	0	-1.832818	0.710873	0.043773
6	0	-0.654811	1.421629	-0.314188
6	0	1.686506	1.416283	-1.025838
6	0	2.852269	0.681670	-1.375005
6	0	2.851918	-0.683698	-1.374853
1	0	3.747349	1.226903	-1.647842
1	0	3.746725	-1.229445	-1.647559
6	0	1.685765	-1.417634	-1.025572
6	0	1.662892	-2.835399	-1.012688
6	0	0.533949	-3.515090	-0.668821
1	0	2.564407	-3.371704	-1.280689
1	0	0.528643	-4.597684	-0.662064
6	0	-0.654741	-2.828146	-0.311021
6	0	-1.843996	-3.514092	0.051905
6	0	-2.971678	-2.832951	0.393263
1	0	-1.837462	-4.596710	0.052649
1	0	-3.871527	-3.368908	0.667903
6	0	-2.998319	-1.413189	0.399275
6	0	-4.160890	-0.679796	0.752955
6	0	-4.160515	0.681854	0.752886
1	0	-5.054915	-1.226480	1.025389
1	0	-5.054242	1.229049	1.025268
6	0	-2.997544	1.414571	0.399124
6	0	-2.970121	2.834318	0.392955
6	0	-1.842071	3.514801	0.051496
1	0	-3.869671	3.370797	0.667553
1	0	-1.834946	4.597415	0.052116
6	0	-0.653201	2.828161	-0.311383
6	0	0.535849	3.514410	-0.669314
6	0	1.664404	2.834061	-1.013149
1	0	0.531132	4.597007	-0.662688
1	0	2.566201	3.369842	-1.281251
17	0	3.765210	-0.000237	1.327223
9	0	4.233472	0.000317	2.890895

M06-2X/BSS-SA

Adduct F–Br---π($C_{24}H_{12}$)

Symmetry C_s: type IC_{Cor}

energy HF = -3595.7165449

Standard orientation

6	0	-0.764421	0.047875	0.711814
6	0	-0.764421	0.047875	-0.711814
6	0	-0.329156	-1.103341	-1.421702
6	0	0.102863	-2.255972	-0.710417
6	0	0.102863	-2.255972	0.710417
6	0	-0.329156	-1.103341	1.421702
6	0	-1.194826	1.184588	1.417914
6	0	-1.629267	2.322630	0.683684
6	0	-1.629267	2.322630	-0.683684
1	0	-1.979785	3.190357	1.228918
1	0	-1.979785	3.190357	-1.228918
6	0	-1.194826	1.184588	-1.417914
6	0	-1.180822	1.163842	-2.835168
6	0	-0.761932	0.060486	-3.515109
1	0	-1.507785	2.046077	-3.370907
1	0	-0.754401	0.056100	-4.597655
6	0	-0.327442	-1.102217	-2.828304
6	0	0.109839	-2.266293	-3.514664
6	0	0.521930	-3.369906	-2.833599
1	0	0.109380	-2.259821	-4.597240
1	0	0.851957	-4.251015	-3.369567
6	0	0.530008	-3.396401	-1.413675
6	0	0.954082	-4.535395	-0.680874
6	0	0.954082	-4.535395	0.680874
1	0	1.279673	-5.411349	-1.227814
1	0	1.279673	-5.411349	1.227814
6	0	0.530008	-3.396401	1.413675
6	0	0.521930	-3.369906	2.833599
6	0	0.109839	-2.266293	3.514664
1	0	0.851957	-4.251015	3.369567
1	0	0.109380	-2.259821	4.597240
6	0	-0.327442	-1.102217	2.828304
6	0	-0.761932	0.060486	3.515109
6	0	-1.180822	1.163842	2.835168
1	0	-0.754401	0.056100	4.597655
1	0	-1.507785	2.046077	3.370907
35	0	0.863667	3.727670	0.000000
9	0	2.378145	4.643569	0.000000

M06-2X/BSS-SA

Adduct F–I---π($C_{24}H_{12}$)

Symmetry C_s: type IC_{Cor}

energy HF = -7942.4673129

Standard orientation

6	0	0.910639	0.286142	0.711833
6	0	0.910639	0.286142	-0.711833
6	0	0.393090	1.402651	-1.421609
6	0	-0.123158	2.519848	-0.710401
6	0	-0.123158	2.519848	0.710401
6	0	0.393090	1.402651	1.421609

6	0	1.416760	-0.818754	1.418180
6	0	1.919580	-1.928719	0.684129
6	0	1.919580	-1.928719	-0.684129
1	0	2.324788	-2.772619	1.229529
1	0	2.324788	-2.772619	-1.229529
6	0	1.416760	-0.818754	-1.418180
6	0	1.400845	-0.799748	-2.835309
6	0	0.905521	0.271553	-3.515061
1	0	1.787368	-1.657866	-3.370606
1	0	0.896644	0.274894	-4.597581
6	0	0.389254	1.400320	-2.828256
6	0	-0.134404	2.528016	-3.514559
6	0	-0.627717	3.597799	-2.833275
1	0	-0.135425	2.520810	-4.597107
1	0	-1.025427	4.450805	-3.368797
6	0	-0.636480	3.624057	-1.413454
6	0	-1.147692	4.726642	-0.680854
6	0	-1.147692	4.726642	0.680854
1	0	-1.542049	5.574068	-1.227509
1	0	-1.542049	5.574068	1.227509
6	0	-0.636480	3.624057	1.413454
6	0	-0.627717	3.597799	2.833275
6	0	-0.134404	2.528016	3.514559
1	0	-1.025427	4.450805	3.368797
1	0	-0.135425	2.520810	4.597107
6	0	0.389254	1.400320	2.828256
6	0	0.905521	0.271553	3.515061
6	0	1.400845	-0.799748	2.835309
1	0	0.896644	0.274894	4.597581
1	0	1.787368	-1.657866	3.370606
53	0	-0.731666	-3.390765	0.000000
9	0	-2.425371	-4.309704	0.000000

M06-2X/BSS-SB
Adduct F–H---π($C_{24}H_{12}$)
Symmetry C_s: type IB_{Cor}
energy HF = -1021.9949333
Standard orientation

6	0	0.238542	-0.534092	1.236336
6	0	0.308388	-1.243601	0.000000
6	0	0.238542	-0.534092	-1.236336
6	0	0.104808	0.885935	-1.235366
6	0	0.039086	1.595405	0.000000
6	0	0.104808	0.885935	1.235366
6	0	0.299559	-1.238017	2.458619
6	0	0.429604	-2.656777	2.432299
6	0	0.498881	-3.339225	1.248822
1	0	0.470263	-3.194588	3.377882
1	0	0.592921	-4.423526	1.242504
6	0	0.442404	-2.652089	0.000000
6	0	0.498881	-3.339225	-1.248822
6	0	0.429604	-2.656777	-2.432299
1	0	0.592921	-4.423526	-1.242504
1	0	0.470263	-3.194588	-3.377882
6	0	0.299559	-1.238017	-2.458619
6	0	0.225963	-0.507101	-3.678843
6	0	0.100191	0.855197	-3.677950
1	0	0.271293	-1.055202	-4.618305

1	0	0.044978	1.402205	-4.617695
6	0	0.036183	1.588002	-2.458178
6	0	-0.097110	3.006085	-2.431674
6	0	-0.158903	3.686912	-1.246635
1	0	-0.150688	3.542927	-3.377322
1	0	-0.261485	4.770712	-1.240409
6	0	-0.093058	3.001120	0.000000
6	0	-0.158903	3.686912	1.246635
6	0	-0.097110	3.006085	2.431674
1	0	-0.261485	4.770712	1.240409
1	0	-0.150688	3.542927	3.377322
6	0	0.036183	1.588002	2.458178
6	0	0.100191	0.855197	3.677950
6	0	0.225963	-0.507101	3.678843
1	0	0.044978	1.402205	4.617695
1	0	0.271293	-1.055202	4.618305
1	0	-1.797651	-2.605688	0.000000
9	0	-2.716717	-2.738601	0.000000

M06-2X/BSS-SB
Adduct Cl–H---π($C_{24}H_{12}$)
Symmetry C_1: type IA_{Cor}
energy HF = -1382.3317575
Standard orientation

6	0	-0.721148	-1.024637	-0.492844
6	0	-1.105442	0.348240	-0.560084
6	0	-0.125255	1.372792	-0.395845
6	0	1.239968	1.023701	-0.177570
6	0	1.625041	-0.348283	-0.117907
6	0	0.644376	-1.372136	-0.272849
6	0	-1.694451	-2.037711	-0.635380
6	0	-3.053021	-1.663999	-0.845003
6	0	-3.422916	-0.348242	-0.911670
1	0	-3.799553	-2.449190	-0.949676
1	0	-4.465512	-0.077559	-1.068874
6	0	-2.460335	0.693758	-0.769848
6	0	-2.821482	2.072147	-0.813771
6	0	-1.880742	3.052552	-0.656645
1	0	-3.866654	2.331211	-0.973334
1	0	-2.170566	4.101403	-0.689562
6	0	-0.508890	2.730519	-0.444499
6	0	0.487670	3.734337	-0.278171
6	0	1.798613	3.399513	-0.074769
1	0	0.186500	4.779832	-0.315677
1	0	2.550690	4.176388	0.050771
6	0	2.209931	2.037191	-0.017742
6	0	3.567425	1.663691	0.198283
6	0	3.936925	0.347742	0.254319
1	0	4.311360	2.448784	0.321323
1	0	4.977881	0.076689	0.422641
6	0	2.976655	-0.692996	0.099020
6	0	3.335541	-2.070117	0.158911
6	0	2.394348	-3.052268	0.012484
1	0	4.379415	-2.329081	0.328026
1	0	2.681723	-4.101148	0.062755
6	0	1.024253	-2.730062	-0.206237
6	0	0.024414	-3.733579	-0.357052
6	0	-1.286496	-3.400343	-0.561350

1	0	0.323154	-4.779176	-0.303611
1	0	-2.040971	-4.177046	-0.671759
1	0	-2.228544	0.207964	1.626917
17	0	-2.232283	-0.012937	2.903375

M06-2X/BSS-SB
Adduct Br–H---π($C_{24}H_{12}$)
Symmetry C_1: type IB_{Cor}
energy HF = -3496.3640857
Standard orientation

6	0	-0.592176	-0.602962	-0.941294
6	0	-0.474344	0.818378	-0.899434
6	0	0.742375	1.421212	-0.460873
6	0	1.843077	0.602299	-0.072071
6	0	1.725915	-0.818520	-0.115591
6	0	0.507371	-1.421059	-0.546283
6	0	-1.801096	-1.199851	-1.361584
6	0	-2.888004	-0.361343	-1.741101
6	0	-2.776127	1.003002	-1.702222
1	0	-3.819777	-0.826561	-2.058262
1	0	-3.617185	1.631475	-1.989491
6	0	-1.567524	1.629898	-1.279358
6	0	-1.429480	3.047054	-1.216292
6	0	-0.261326	3.623306	-0.799260
1	0	-2.275682	3.666528	-1.507855
1	0	-0.168925	4.707169	-0.755774
6	0	0.855156	2.827511	-0.410914
6	0	2.080471	3.405233	0.028573
6	0	3.137678	2.619501	0.398925
1	0	2.161834	4.490167	0.066083
1	0	4.068820	3.073384	0.734390
6	0	3.047961	1.198767	0.358958
6	0	4.133836	0.359843	0.741485
6	0	4.021943	-1.002928	0.698549
1	0	5.060899	0.825000	1.072780
1	0	4.859518	-1.632197	0.994560
6	0	2.815709	-1.628689	0.270860
6	0	2.672222	-3.044843	0.222865
6	0	1.501176	-3.622799	-0.185507
1	0	3.516147	-3.664546	0.520997
1	0	1.403935	-4.706696	-0.214599
6	0	0.387698	-2.827160	-0.579874
6	0	-0.842720	-3.404704	-1.006987
6	0	-1.898409	-2.620593	-1.382634
1	0	-0.929698	-4.489660	-1.027607
1	0	-2.834169	-3.074226	-1.704758
1	0	-2.425616	0.532938	0.902526
35	0	-2.704240	-0.015317	2.195811

M06-2X/BSS-SB
Adduct I–H---π($C_{24}H_{12}$)
Symmetry C_1: type IB_{Cor}
energy HF = -7843.0808655
Standard orientation

6	0	0.315653	-0.455257	1.262471
6	0	0.065750	0.943137	1.132021

6	0	-1.132899	1.398783	0.506529
6	0	-2.084982	0.455610	0.018529
6	0	-1.835455	-0.942468	0.149856
6	0	-0.634164	-1.397719	0.768748
6	0	1.513341	-0.906152	1.859157
6	0	2.456078	0.054805	2.324079
6	0	2.216889	1.397049	2.201213
1	0	3.381198	-0.299235	2.775728
1	0	2.947905	2.120626	2.557817
6	0	1.016505	1.877784	1.601402
6	0	0.750635	3.269264	1.446183
6	0	-0.401489	3.704720	0.851160
1	0	1.485677	3.984244	1.811586
1	0	-0.591634	4.770681	0.737745
6	0	-1.373093	2.782868	0.364595
6	0	-2.577536	3.212441	-0.262349
6	0	-3.493606	2.307578	-0.724980
1	0	-2.757733	4.280853	-0.368713
1	0	-4.410522	2.649538	-1.202453
6	0	-3.271843	0.906384	-0.599094
6	0	-4.206657	-0.055026	-1.079856
6	0	-3.967003	-1.396116	-0.954688
1	0	-5.120856	0.298559	-1.553918
1	0	-4.688335	-2.120223	-1.329487
6	0	-2.775433	-1.875889	-0.338951
6	0	-2.499895	-3.266470	-0.202516
6	0	-1.344862	-3.703117	0.386745
1	0	-3.228018	-3.981700	-0.581214
1	0	-1.145345	-4.769188	0.481513
6	0	-0.380500	-2.781404	0.885945
6	0	0.833394	-3.210615	1.495501
6	0	1.747308	-2.307059	1.963452
1	0	1.024317	-4.279134	1.580103
1	0	2.674054	-2.648783	2.421286
1	0	2.134489	0.921756	-0.568112
53	0	2.541094	-0.018996	-1.831940

M06-2X/BSS-SB
Adduct Cl–H---π($C_{24}H_{12}$)
Symmetry C_1: type IC_{Cor}
energy HF = -1382.3316
Standard orientation

6	0	-0.908756	-0.713472	-0.531247
6	0	-0.908784	0.713422	-0.531253
6	0	0.306631	1.426380	-0.310305
6	0	1.522538	0.713194	-0.095731
6	0	1.522567	-0.713144	-0.095726
6	0	0.306687	-1.426380	-0.310295
6	0	-2.113022	-1.421051	-0.741212
6	0	-3.315755	-0.685472	-0.949676
6	0	-3.315783	0.685323	-0.949682
1	0	-4.243277	-1.233809	-1.106004
1	0	-4.243326	1.233622	-1.106013
6	0	-2.113079	1.420952	-0.741224
6	0	-2.089917	2.845091	-0.725094
6	0	-0.923437	3.527743	-0.515815
1	0	-3.022613	3.383550	-0.883686
1	0	-0.918353	4.616321	-0.506137
6	0	0.304921	2.838130	-0.303643

6	0	1.531618	3.527465	-0.083985
6	0	2.698918	2.843083	0.118480
1	0	1.524548	4.616067	-0.079675
1	0	3.630161	3.381860	0.285373
6	0	2.726190	1.419086	0.118851
6	0	3.927719	0.684134	0.331294
6	0	3.927746	-0.683985	0.331298
1	0	4.853113	1.233374	0.496736
1	0	4.853162	-1.233187	0.496743
6	0	2.726247	-1.418986	0.118860
6	0	2.699032	-2.842984	0.118499
6	0	1.531759	-3.527415	-0.083961
1	0	3.630297	-3.381724	0.285394
1	0	1.524733	-4.616017	-0.079643
6	0	0.305034	-2.838130	-0.303623
6	0	-0.923296	-3.527793	-0.515789
6	0	-2.089804	-2.845189	-0.725072
1	0	-0.918168	-4.616372	-0.506104
1	0	-3.022478	-3.383686	-0.883661
1	0	-2.891462	-0.000022	1.458186
17	0	-2.633657	0.000001	2.728516

M06-2X/BSS-SA
Adduct F–H---π($C_{24}H_{12}$)
Symmetry C_{2v}: type ID_{Cor}
energy HF = -1022.1882186
Standard orientation

6	0	0.000000	1.422667	-0.193390
6	0	1.232054	0.711457	-0.193543
6	0	1.232054	-0.711457	-0.193543
6	0	0.000000	-1.422667	-0.193390
6	0	-1.232054	-0.711457	-0.193543
6	0	-1.232054	0.711457	-0.193543
6	0	0.000000	2.829530	-0.184049
6	0	1.242715	3.514427	-0.179096
6	0	2.421869	2.833820	-0.179077
1	0	1.236647	4.596975	-0.172217
1	0	3.362212	3.370401	-0.172160
6	0	2.450327	1.414896	-0.184160
6	0	3.664872	0.680765	-0.179244
6	0	3.664872	-0.680765	-0.179244
1	0	4.599443	1.227295	-0.172490
1	0	4.599443	-1.227295	-0.172490
6	0	2.450327	-1.414896	-0.184160
6	0	2.421869	-2.833820	-0.179077
6	0	1.242715	-3.514427	-0.179096
1	0	3.362212	-3.370401	-0.172160
1	0	1.236647	-4.596975	-0.172217
6	0	0.000000	-2.829530	-0.184049
6	0	-1.242715	-3.514427	-0.179096
6	0	-2.421869	-2.833820	-0.179077
1	0	-1.236647	-4.596975	-0.172217
1	0	-3.362212	-3.370401	-0.172160
6	0	-2.450327	-1.414896	-0.184160
6	0	-3.664872	-0.680765	-0.179244
6	0	-3.664872	0.680765	-0.179244
1	0	-4.599443	-1.227295	-0.172490
1	0	-4.599443	1.227295	-0.172490
6	0	-2.450327	1.414896	-0.184160

6	0	-2.421869	2.833820	-0.179077
6	0	-1.242715	3.514427	-0.179096
1	0	-3.362212	3.370401	-0.172160
1	0	-1.236647	4.596975	-0.172217
1	0	0.000000	0.000000	2.025986
9	0	0.000000	0.000000	2.948182

M06-2X/BSS-SA
Adduct Cl–H---π($C_{24}H_{12}$)
Symmetry C_{2v}: type ID_{Cor}
energy HF = -1382.5035303
Standard orientation

6	0	0.000000	1.422388	-0.377401
6	0	1.231739	0.711302	-0.377309
6	0	1.231739	-0.711302	-0.377309
6	0	0.000000	-1.422388	-0.377401
6	0	-1.231739	-0.711302	-0.377309
6	0	-1.231739	0.711302	-0.377309
6	0	0.000000	2.829108	-0.364251
6	0	1.242670	3.514315	-0.356311
6	0	2.421769	2.833706	-0.356224
1	0	1.236479	4.596874	-0.346784
1	0	3.362204	3.370149	-0.346598
6	0	2.449904	1.414667	-0.364185
6	0	3.664700	0.680742	-0.356274
6	0	3.664700	-0.680742	-0.356274
1	0	4.599215	1.227394	-0.346863
1	0	4.599215	-1.227394	-0.346863
6	0	2.449904	-1.414667	-0.364185
6	0	2.421769	-2.833706	-0.356224
6	0	1.242670	-3.514315	-0.356311
1	0	3.362204	-3.370149	-0.346598
1	0	1.236479	-4.596874	-0.346784
6	0	0.000000	-2.829108	-0.364251
6	0	-1.242670	-3.514315	-0.356311
6	0	-2.421769	-2.833706	-0.356224
1	0	-1.236479	-4.596874	-0.346784
1	0	-3.362204	-3.370149	-0.346598
6	0	-2.449904	-1.414667	-0.364185
6	0	-3.664700	-0.680742	-0.356274
6	0	-3.664700	0.680742	-0.356274
1	0	-4.599215	-1.227394	-0.346863
1	0	-4.599215	1.227394	-0.346863
6	0	-2.449904	1.414667	-0.364185
6	0	-2.421769	2.833706	-0.356224
6	0	-1.242670	3.514315	-0.356311
1	0	-3.362204	3.370149	-0.346598
1	0	-1.236479	4.596874	-0.346784
1	0	0.000000	0.000000	1.929129
17	0	0.000000	0.000000	3.210527

M06-2X/BSS-SA
Adduct Br–H---π($C_{24}H_{12}$)
Symmetry C_{2v}: type ID_{Cor}
energy HF = -3496.5355791
Standard orientation

6	0	0.000000	1.422324	-0.710761
6	0	1.231682	0.711270	-0.710699

6	0	1.231682	-0.711270	-0.710699
6	0	0.000000	-1.422324	-0.710761
6	0	-1.231682	-0.711270	-0.710699
6	0	-1.231682	0.711270	-0.710699
6	0	0.000000	2.828997	-0.696473
6	0	1.242660	3.514288	-0.687790
6	0	2.421753	2.833686	-0.687890
1	0	1.236438	4.596849	-0.677464
1	0	3.362205	3.370104	-0.677619
6	0	2.449813	1.414617	-0.696711
6	0	3.664675	0.680737	-0.688276
6	0	3.664675	-0.680737	-0.688276
1	0	4.599180	1.227415	-0.678323
1	0	4.599180	-1.227415	-0.678323
6	0	2.449813	-1.414617	-0.696711
6	0	2.421753	-2.833686	-0.687890
6	0	1.242660	-3.514288	-0.687790
1	0	3.362205	-3.370104	-0.677619
1	0	1.236438	-4.596849	-0.677464
6	0	0.000000	-2.828997	-0.696473
6	0	-1.242660	-3.514288	-0.687790
6	0	-2.421753	-2.833686	-0.687890
1	0	-1.236438	-4.596849	-0.677464
1	0	-3.362205	-3.370104	-0.677619
6	0	-2.449813	-1.414617	-0.696711
6	0	-3.664675	-0.680737	-0.688276
6	0	-3.664675	0.680737	-0.688276
1	0	-4.599180	-1.227415	-0.678323
1	0	-4.599180	1.227415	-0.678323
6	0	-2.449813	1.414617	-0.696711
6	0	-2.421753	2.833686	-0.687890
6	0	-1.242660	3.514288	-0.687790
1	0	-3.362205	3.370104	-0.677619
1	0	-1.236438	4.596849	-0.677464
1	0	0.000000	0.000000	1.625563
35	0	0.000000	0.000000	3.048790

M06-2X/BSS-SA
Adduct I–H---π($C_{24}H_{12}$)
Symmetry C_{2v}: type ID_{Cor}
energy HF = -7843.2522946
Standard orientation

6	0	0.000000	1.422184	-1.030506
6	0	1.231585	0.711204	-1.030676
6	0	1.231585	-0.711204	-1.030676
6	0	0.000000	-1.422184	-1.030506
6	0	-1.231585	-0.711204	-1.030676
6	0	-1.231585	0.711204	-1.030676
6	0	0.000000	2.828766	-1.013560
6	0	1.242634	3.514209	-1.003174
6	0	2.421722	2.833634	-1.003825
1	0	1.236316	4.596760	-0.990891
1	0	3.362208	3.369984	-0.992037
6	0	2.449664	1.414521	-1.014758
6	0	3.664647	0.680730	-1.005250
6	0	3.664647	-0.680730	-1.005250
1	0	4.599111	1.227474	-0.994059
1	0	4.599111	-1.227474	-0.994059
6	0	2.449664	-1.414521	-1.014758

6	0	2.421722	-2.833634	-1.003825
6	0	1.242634	-3.514209	-1.003174
1	0	3.362208	-3.369984	-0.992037
1	0	1.236316	-4.596760	-0.990891
6	0	0.000000	-2.828766	-1.013560
6	0	-1.242634	-3.514209	-1.003174
6	0	-2.421722	-2.833634	-1.003825
1	0	-1.236316	-4.596760	-0.990891
1	0	-3.362208	-3.369984	-0.992037
6	0	-2.449664	-1.414521	-1.014758
6	0	-3.664647	-0.680730	-1.005250
6	0	-3.664647	0.680730	-1.005250
1	0	-4.599111	-1.227474	-0.994059
1	0	-4.599111	1.227474	-0.994059
6	0	-2.449664	1.414521	-1.014758
6	0	-2.421722	2.833634	-1.003825
6	0	-1.242634	3.514209	-1.003174
1	0	-3.362208	3.369984	-0.992037
1	0	-1.236316	4.596760	-0.990891
1	0	0.000000	0.000000	1.334918
53	0	0.000000	0.000000	2.952571

M06-2X/BSS-SA
Adduct F–F---π($C_{24}H_{12}$)
Symmetry C_{2v}: type ID_{Cor}
energy HF = -1121.239786
Standard orientation

6	0	0.000000	1.421631	-0.375822
6	0	1.231199	0.710943	-0.376177
6	0	1.231199	-0.710943	-0.376177
6	0	0.000000	-1.421631	-0.375822
6	0	-1.231199	-0.710943	-0.376177
6	0	-1.231199	0.710943	-0.376177
6	0	0.000000	2.828103	-0.369664
6	0	1.242583	3.514081	-0.366203
6	0	2.421658	2.833552	-0.366532
1	0	1.236124	4.596775	-0.362246
1	0	3.362304	3.369900	-0.362829
6	0	2.449143	1.414205	-0.370365
6	0	3.664543	0.680714	-0.367161
6	0	3.664543	-0.680714	-0.367161
1	0	4.599055	1.227638	-0.363638
1	0	4.599055	-1.227638	-0.363638
6	0	2.449143	-1.414205	-0.370365
6	0	2.421658	-2.833552	-0.366532
6	0	1.242583	-3.514081	-0.366203
1	0	3.362304	-3.369900	-0.362829
1	0	1.236124	-4.596775	-0.362246
6	0	0.000000	-2.828103	-0.369664
6	0	-1.242583	-3.514081	-0.366203
6	0	-2.421658	-2.833552	-0.366532
1	0	-1.236124	-4.596775	-0.362246
1	0	-3.362304	-3.369900	-0.362829
6	0	-2.449143	-1.414205	-0.370365
6	0	-3.664543	-0.680714	-0.367161
6	0	-3.664543	0.680714	-0.367161
1	0	-4.599055	-1.227638	-0.363638
1	0	-4.599055	1.227638	-0.363638
6	0	-2.449143	1.414205	-0.370365

6	0	-2.421658	2.833552	-0.366532
6	0	-1.242583	3.514081	-0.366203
1	0	-3.362304	3.369900	-0.362829
1	0	-1.236124	4.596775	-0.362246
9	0	0.000000	0.000000	2.518092
9	0	0.000000	0.000000	3.883597

M06-2X/BSS-SA
Adduct Cl–Cl---π($C_{24}H_{12}$)
Symmetry C_{2v}: type ID_{Cor}
energy HF = -1842.0225332
Standard orientation

6	0	0.000000	1.421890	-0.745633
6	0	1.231394	0.711059	-0.746195
6	0	1.231394	-0.711059	-0.746195
6	0	0.000000	-1.421890	-0.745633
6	0	-1.231394	-0.711059	-0.746195
6	0	-1.231394	0.711059	-0.746195
6	0	0.000000	2.828213	-0.728965
6	0	1.242534	3.513973	-0.718590
6	0	2.421607	2.833463	-0.719424
1	0	1.236078	4.596579	-0.706834
1	0	3.362180	3.369773	-0.708240
6	0	2.449265	1.414281	-0.730528
6	0	3.664444	0.680707	-0.720773
6	0	3.664444	-0.680707	-0.720773
1	0	4.598906	1.227564	-0.710003
1	0	4.598906	-1.227564	-0.710003
6	0	2.449265	-1.414281	-0.730528
6	0	2.421607	-2.833463	-0.719424
6	0	1.242534	-3.513973	-0.718590
1	0	3.362180	-3.369773	-0.708240
1	0	1.236078	-4.596579	-0.706834
6	0	0.000000	-2.828213	-0.728965
6	0	-1.242534	-3.513973	-0.718590
6	0	-2.421607	-2.833463	-0.719424
1	0	-1.236078	-4.596579	-0.706834
1	0	-3.362180	-3.369773	-0.708240
6	0	-2.449265	-1.414281	-0.730528
6	0	-3.664444	-0.680707	-0.720773
6	0	-3.664444	0.680707	-0.720773
1	0	-4.598906	-1.227564	-0.710003
1	0	-4.598906	1.227564	-0.710003
6	0	-2.449265	1.414281	-0.730528
6	0	-2.421607	2.833463	-0.719424
6	0	-1.242534	3.513973	-0.718590
1	0	-3.362180	3.369773	-0.708240
1	0	-1.236078	4.596579	-0.706834
17	0	0.000000	0.000000	2.340898
17	0	0.000000	0.000000	4.332496

M06-2X/BSS-SA
Adduct Br–Br---π($C_{24}H_{12}$)
Symmetry C_{2v}: type ID_{Cor}
energy HF = -6070.1190755
Standard orientation

6	0	0.000000	1.421979	-1.356013
6	0	1.231568	0.711137	-1.357043

6	0	1.231568	-0.711137	-1.357043
6	0	0.000000	-1.421979	-1.356013
6	0	-1.231568	-0.711137	-1.357043
6	0	-1.231568	0.711137	-1.357043
6	0	0.000000	2.828249	-1.335731
6	0	1.242527	3.513919	-1.322846
6	0	2.421625	2.833468	-1.324376
1	0	1.236040	4.596469	-1.307993
1	0	3.362141	3.369779	-1.310599
6	0	2.449404	1.414357	-1.338459
6	0	3.664485	0.680704	-1.326840
6	0	3.664485	-0.680704	-1.326840
1	0	4.598923	1.227516	-1.313728
1	0	4.598923	-1.227516	-1.313728
6	0	2.449404	-1.414357	-1.338459
6	0	2.421625	-2.833468	-1.324376
6	0	1.242527	-3.513919	-1.322846
1	0	3.362141	-3.369779	-1.310599
1	0	1.236040	-4.596469	-1.307993
6	0	0.000000	-2.828249	-1.335731
6	0	-1.242527	-3.513919	-1.322846
6	0	-2.421625	-2.833468	-1.324376
1	0	-1.236040	-4.596469	-1.307993
1	0	-3.362141	-3.369779	-1.310599
6	0	-2.449404	-1.414357	-1.338459
6	0	-3.664485	-0.680704	-1.326840
6	0	-3.664485	0.680704	-1.326840
1	0	-4.598923	-1.227516	-1.313728
1	0	-4.598923	1.227516	-1.313728
6	0	-2.449404	1.414357	-1.338459
6	0	-2.421625	2.833468	-1.324376
6	0	-1.242527	3.513919	-1.322846
1	0	-3.362141	3.369779	-1.310599
1	0	-1.236040	4.596469	-1.307993
35	0	0.000000	0.000000	1.828746
35	0	0.000000	0.000000	4.116961

M06-2X/BSS-SA
Adduct I–I---π($C_{24}H_{12}$)
Symmetry C_{2v}: type ID_{Cor}
energy HF = -14763.5889633
Standard orientation

6	0	0.000000	1.421952	-1.912274
6	0	1.231631	0.711122	-1.913660
6	0	1.231631	-0.711122	-1.913660
6	0	0.000000	-1.421952	-1.912274
6	0	-1.231631	-0.711122	-1.913660
6	0	-1.231631	0.711122	-1.913660
6	0	0.000000	2.828184	-1.887237
6	0	1.242494	3.513788	-1.870362
6	0	2.421585	2.833372	-1.871998
1	0	1.235938	4.596256	-1.851488
1	0	3.362063	3.369591	-1.854138
6	0	2.449408	1.414342	-1.890055
6	0	3.664416	0.680689	-1.874569
6	0	3.664416	-0.680689	-1.874569
1	0	4.598768	1.227498	-1.857443
1	0	4.598768	-1.227498	-1.857443
6	0	2.449408	-1.414342	-1.890055

6	0	2.421585	-2.833372	-1.871998
6	0	1.242494	-3.513788	-1.870362
1	0	3.362063	-3.369591	-1.854138
1	0	1.235938	-4.596256	-1.851488
6	0	0.000000	-2.828184	-1.887237
6	0	-1.242494	-3.513788	-1.870362
6	0	-2.421585	-2.833372	-1.871998
1	0	-1.235938	-4.596256	-1.851488
1	0	-3.362063	-3.369591	-1.854138
6	0	-2.449408	-1.414342	-1.890055
6	0	-3.664416	-0.680689	-1.874569
6	0	-3.664416	0.680689	-1.874569
1	0	-4.598768	-1.227498	-1.857443
1	0	-4.598768	1.227498	-1.857443
6	0	-2.449408	1.414342	-1.890055
6	0	-2.421585	2.833372	-1.871998
6	0	-1.242494	3.513788	-1.870362
1	0	-3.362063	3.369591	-1.854138
1	0	-1.235938	4.596256	-1.851488
53	0	0.000000	0.000000	1.435708
53	0	0.000000	0.000000	4.110365

M06-2X/BSS-SA
Adduct F–Cl---π($C_{24}H_{12}$)
Symmetry C_{2v}: type ID_{Cor}
energy HF = -1481.65761
Standard orientation

6	0	0.000000	1.422121	-0.532956
6	0	1.231596	0.711182	-0.533564
6	0	1.231596	-0.711182	-0.533564
6	0	0.000000	-1.422121	-0.532956
6	0	-1.231596	-0.711182	-0.533564
6	0	-1.231596	0.711182	-0.533564
6	0	0.000000	2.828468	-0.514767
6	0	1.242565	3.514049	-0.503487
6	0	2.421658	2.833532	-0.504405
1	0	1.236184	4.596611	-0.490587
1	0	3.362158	3.369885	-0.492141
6	0	2.449489	1.414415	-0.516493
6	0	3.664529	0.680718	-0.505919
6	0	3.664529	-0.680718	-0.505919
1	0	4.598996	1.227483	-0.494112
1	0	4.598996	-1.227483	-0.494112
6	0	2.449489	-1.414415	-0.516493
6	0	2.421658	-2.833532	-0.504405
6	0	1.242565	-3.514049	-0.503487
1	0	3.362158	-3.369885	-0.492141
1	0	1.236184	-4.596611	-0.490587
6	0	0.000000	-2.828468	-0.514767
6	0	-1.242565	-3.514049	-0.503487
6	0	-2.421658	-2.833532	-0.504405
1	0	-1.236184	-4.596611	-0.490587
1	0	-3.362158	-3.369885	-0.492141
6	0	-2.449489	-1.414415	-0.516493
6	0	-3.664529	-0.680718	-0.505919
6	0	-3.664529	0.680718	-0.505919
1	0	-4.598996	-1.227483	-0.494112
1	0	-4.598996	1.227483	-0.494112
6	0	-2.449489	1.414415	-0.516493

6	0	-2.421658	2.833532	-0.504405
6	0	-1.242565	3.514049	-0.503487
1	0	-3.362158	3.369885	-0.492141
1	0	-1.236184	4.596611	-0.490587
17	0	0.000000	0.000000	2.515831
9	0	0.000000	0.000000	4.138196

M06-2X/BSS-SA
Adduct F–Br---π($C_{24}H_{12}$)
Symmetry C_{2v}: type ID_{Cor}
energy HF = -3595.7125296
Standard orientation

6	0	0.000000	1.422342	-0.788889
6	0	1.231893	0.711329	-0.790119
6	0	1.231893	-0.711329	-0.790119
6	0	0.000000	-1.422342	-0.788889
6	0	-1.231893	-0.711329	-0.790119
6	0	-1.231893	0.711329	-0.790119
6	0	0.000000	2.828623	-0.766550
6	0	1.242590	3.514046	-0.752474
6	0	2.421711	2.833591	-0.754253
1	0	1.236201	4.596529	-0.736060
1	0	3.362125	3.369956	-0.739099
6	0	2.449739	1.414552	-0.769737
6	0	3.664641	0.680714	-0.757143
6	0	3.664641	-0.680714	-0.757143
1	0	4.599083	1.227400	-0.742758
1	0	4.599083	-1.227400	-0.742758
6	0	2.449739	-1.414552	-0.769737
6	0	2.421711	-2.833591	-0.754253
6	0	1.242590	-3.514046	-0.752474
1	0	3.362125	-3.369956	-0.739099
1	0	1.236201	-4.596529	-0.736060
6	0	0.000000	-2.828623	-0.766550
6	0	-1.242590	-3.514046	-0.752474
6	0	-2.421711	-2.833591	-0.754253
1	0	-1.236201	-4.596529	-0.736060
1	0	-3.362125	-3.369956	-0.739099
6	0	-2.449739	-1.414552	-0.769737
6	0	-3.664641	-0.680714	-0.757143
6	0	-3.664641	0.680714	-0.757143
1	0	-4.599083	-1.227400	-0.742758
1	0	-4.599083	1.227400	-0.742758
6	0	-2.449739	1.414552	-0.769737
6	0	-2.421711	2.833591	-0.754253
6	0	-1.242590	3.514046	-0.752474
1	0	-3.362125	3.369956	-0.739099
1	0	-1.236201	4.596529	-0.736060
35	0	0.000000	0.000000	2.351971
9	0	0.000000	0.000000	4.109706

M06-2X/BSS-SA
Adduct F–I---π($C_{24}H_{12}$)
Symmetry C_{2v}: type ID_{Cor}
energy HF = -7942.4631379
Standard orientation

6	0	0.000000	1.422483	-1.034148
6	0	1.232055	0.711378	-1.035719

6	0	1.232055	-0.711378	-1.035719
6	0	0.000000	-1.422483	-1.034148
6	0	-1.232055	-0.711378	-1.035719
6	0	-1.232055	0.711378	-1.035719
6	0	0.000000	2.828682	-1.005727
6	0	1.242556	3.513926	-0.986710
6	0	2.421665	2.833486	-0.988566
1	0	1.236136	4.596289	-0.965240
1	0	3.362006	3.369745	-0.968274
6	0	2.449814	1.414586	-1.008980
6	0	3.664557	0.680700	-0.991508
6	0	3.664557	-0.680700	-0.991508
1	0	4.598897	1.227341	-0.972041
1	0	4.598897	-1.227341	-0.972041
6	0	2.449814	-1.414586	-1.008980
6	0	2.421665	-2.833486	-0.988566
6	0	1.242556	-3.513926	-0.986710
1	0	3.362006	-3.369745	-0.968274
1	0	1.236136	-4.596289	-0.965240
6	0	0.000000	-2.828682	-1.005727
6	0	-1.242556	-3.513926	-0.986710
6	0	-2.421665	-2.833486	-0.988566
1	0	-1.236136	-4.596289	-0.965240
1	0	-3.362006	-3.369745	-0.968274
6	0	-2.449814	-1.414586	-1.008980
6	0	-3.664557	-0.680700	-0.991508
6	0	-3.664557	0.680700	-0.991508
1	0	-4.598897	-1.227341	-0.972041
1	0	-4.598897	1.227341	-0.972041
6	0	-2.449814	1.414586	-1.008980
6	0	-2.421665	2.833486	-0.988566
6	0	-1.242556	3.513926	-0.986710
1	0	-3.362006	3.369745	-0.968274
1	0	-1.236136	4.596289	-0.965240
53	0	0.000000	0.000000	2.244261
9	0	0.000000	0.000000	4.158939
